# Identification and molecular typing of disulfidptosis-related biomarkers in anaplastic thyroid carcinoma

**DOI:** 10.1038/s41420-026-03089-9

**Published:** 2026-04-22

**Authors:** Weidong Teng, Yawen Guo, Lingling Ding, Aoni Zhou, Yehao Guo, Jiantong He, Lirong zhang, Haolan Yu, Zekai Tao, Jiafeng Wang, Jiajie Xu, Zhuo Tan, Liehao Jiang

**Affiliations:** 1 Otolaryngology & Head and Neck Center, Cancer Center, Department of Head and Neck Surgery, Zhejiang Provincial People’s Hospital (Affiliated People’s Hospital), Hangzhou Medical College, Hangzhou, China; 2 Hangzhou Normal University, Hangzhou, China; 3 Zhejiang Provincial Clinical Research Center for Head & Neck Cancer, Hangzhou, China; 4 Zhejiang Key Laboratory of Precision Medicine Research on Head & Neck Cancer, Hangzhou, China; 5 Hangzhou Medical College, Hangzhou, China; 6 Wenzhou Medical University, Wenzhou, China; 7 Zhejiang Chinese Medical University, Hangzhou, China

**Keywords:** Cancer, Cell death

## Abstract

Increasing evidence suggests that disulfidptosis plays a crucial role in tumorigenesis and progression. This study aimed to identify biomarkers closely associated with disulfidptosis in anaplastic thyroid carcinoma (ATC). Utilizing ATC-related datasets (GSE65144, GSE9115, GSE27155, and GSE53072) in conjunction with disulfide bond-related genes (DRGs) identified in the literature, differentially expressed genes (DEGs) were screened from the GSE65144 and GSE9115 datasets. A total of 113 common DEGs were identified through cross-sectional analysis. Weighted gene co-expression network analysis (WGCNA) was employed to screen genes related to disulfidptosis and ATC, and five biomarkers—ATP1B3, TFF3, LGALS1, ADAM12, and COL1A2—were identified using machine learning algorithms. A nomogram model constructed based on these markers demonstrated high accuracy. In vitro validation revealed that ATP1B3 knockdown significantly inhibited tumor growth, indicating its potential anti-ATC activity. Furthermore, laser confocal microscopy, flow cytometry, and other experimental methods suggested a correlation between ATP1B3 and disulfidptosis. These findings highlight ATP1B3, TFF3, LGALS1, ADAM12, and COL1A2 as potential disulfidptosis-related biomarkers in ATC. This study provides a theoretical foundation for understanding the role of disulfidptosis in ATC pathogenesis and suggests that ATP1B3 may serve as a promising therapeutic target.

## Introduction

Anaplastic thyroid carcinoma (ATC) is a highly aggressive malignancy originating from the epithelial cells of thyroid follicles. It is characterized by extensive local invasion, necrosis, and hemorrhage [1]. Clinically, ATC predominantly affects elderly women over 65 years old [2], and typically presents as a rapidly enlarging neck mass with associated symptoms such as neck pain, dysphagia, and airway compression. Approximately 50% of ATC patients succumb to airway obstruction or asphyxia, and nearly 40% present with lymph node or distant metastases at diagnosis [1]. Despite the availability of surgical intervention, radiotherapy, chemotherapy, and targeted therapy, only a minority of patients qualify for surgery, and radiotherapy provides limited efficacy. Single-modality treatment is often insufficient to control disease progression or improve prognosis, leading to poor survival outcomes [3–5]. Thus, there is an urgent need to identify novel biomarkers and elucidate potential mechanisms to enhance our understanding of ATC pathogenesis and develop targeted therapeutic strategies.

Disulfidptosis is a newly characterized mode of cell death mediated by SLC7A11-induced disulfide stress, triggered by excessive intracellular cystine accumulation. In cells with high SLC7A11 expression under glucose-deficient conditions, excessive cystine accumulation leads to the formation of aberrant disulfide bonds between actin cytoskeletal proteins, resulting in cytoskeletal contraction, detachment from the cell membrane, and eventual cell death. Current research on disulfidptosis primarily focuses on its prognostic implications and therapeutic relevance in malignancies such as lung adenocarcinoma and colorectal cancer [6, 7]. However, the involvement of disulfidptosis-related genes in ATC remains unclear.

This study aims to identify and characterize disulfidptosis-related biomarkers in ATC through bioinformatics analysis, thereby uncovering potential therapeutic targets involved in ATC-associated disulfidptosis.

## Results

### Common DEGs were obtained by intersection genes of DEGs

**Fig. 1:**
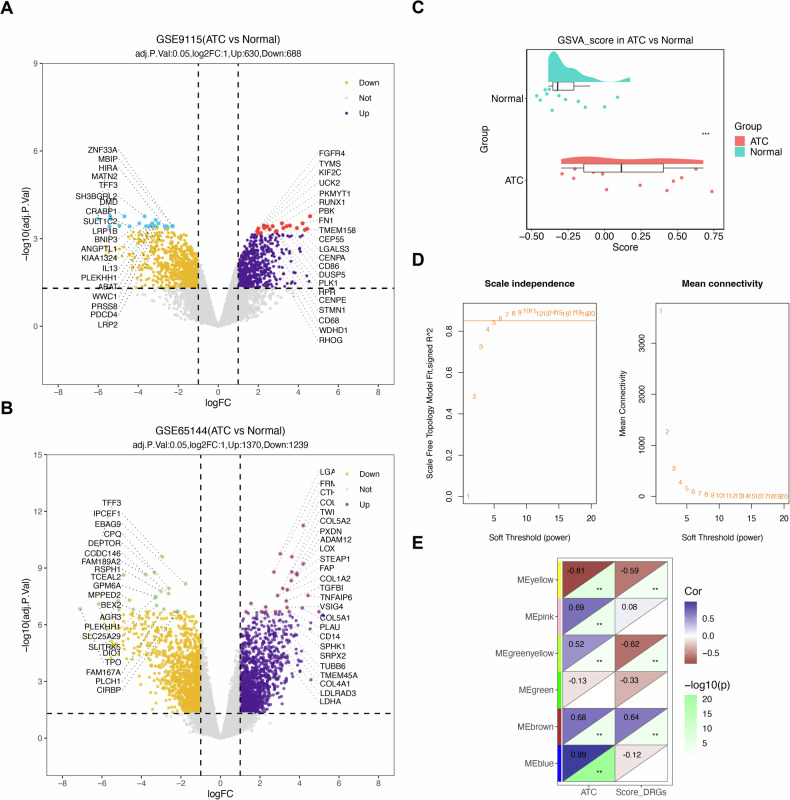
Differentially expressed genes and module genes obtained. **A** Volcano plot of differentially expressed genes in the GSE65144 dataset. Each point represented a gene, with yellow and purple points denoting significantly differentially expressed genes. Purple points indicated upregulated differential expression, yellow points indicated downregulated differential expression, and gray points signified no significant difference in these genes. The red and green points marked in the figure were hypervariable genes, with green representing down-regulation and red representing upregulation. **B** Volcano plot of differentially expressed genes in the GSE9115 dataset. **C** Difference in disulfidptosis scores between ATC and Normal groups. **D** Screening of the optimal soft threshold. The ordinate in the left panel was the scale-free fit index, namely signed R2, representing the square of the correlation coefficient between log(k) and log(p(k)) in the corresponding network multiplied by a direction vector. The ordinate in the right panel represented the mean value of the number of gene connections (i.e., the degree of nodes) in the corresponding network. **E** Correlation between modules and (disulfidptosis score, ATC, and Normal). Each row corresponded to a module ME value, each column corresponded to a trait, and each cell contained the corresponding correlation and *P* value. The bluer the module, the higher the positive correlation between the phenotypic trait and the genes in that module, while the browner the color, the higher the negative correlation. ** represented *P* < 0.01.

In the GSE9115 dataset, 1318 DEGs were detected, including 630 upregulated and 688 downregulated genes (Fig. 1A and Supplementary Fig. 1A). Similarly, in the GSE65144 dataset, a total of 2609 DEGs were identified between ATC and normal thyroid tissue samples, comprising 1370 upregulated and 1239 downregulated genes (Fig. 1B and Supplementary Fig. 1B). By intersecting the DEGs from both datasets, 113 common DEGs were identified (Table S2).

### Disulfidptosis and ATC-related genes were obtained through WGCNA

GSVA analysis using DRGs as the reference gene set demonstrated a significant difference in disulfidptosis scores between ATC and normal tissue samples (Fig. 1C). To identify genes associated with both disulfidptosis and ATC, WGCNA was performed. Clustering analysis of the samples confirmed the absence of outliers (Supplementary Fig. 1C). The optimal soft threshold was determined as 6 (*R*² = 0.861) for network topology construction (Fig. 1D). Gene modules were generated using the dynamic tree-cutting method with a clipping height of 0.4 (Supplementary Fig. 1D, E). Among these, the brown module exhibited the strongest correlation with disulfidptosis scores and ATC (|cor | > 0.6, *P* < 0.05) and was designated as the key module. A total of 2990 genes from this module were identified as disulfidptosis- and ATC-related genes for further analysis (Fig. 1E).

### Candidate genes were identified by intersection genes of common DEGs and disulfidptosis- and ATC-related genes

A total of 32 candidate genes were identified by intersecting common DEGs with disulfidptosis- and ATC-related genes (Supplementary Fig. 2A). Chromosomal localization analysis indicated that these genes were primarily located on chromosomes 1, 3, 7, 10, 20, and 22 (Supplementary Fig. 2B). The PPI network analysis revealed a network comprising 20 nodes and 68 edges (Supplementary Fig. 2C), with ZWINT, CENPA, and PRC1 emerging as core target genes.

Functional enrichment analysis was conducted on 32 candidate genes to determine their potential biological roles, identifying a total of 422 GO terms and 86 KEGG pathways. GO analysis revealed significant enrichment in biological processes such as nucleoside monophosphate biosynthesis, mitotic nuclear division, and microtubule cytoskeleton organization during mitosis (Supplementary Fig. 2D). KEGG pathway analysis indicated that candidate genes were primarily involved in nucleotide metabolism, axon guidance, and the p53 signaling pathway (Supplementary Fig. 2E).

### Biomarkers were obtained by machine learning algorithms

**Fig. 2:**
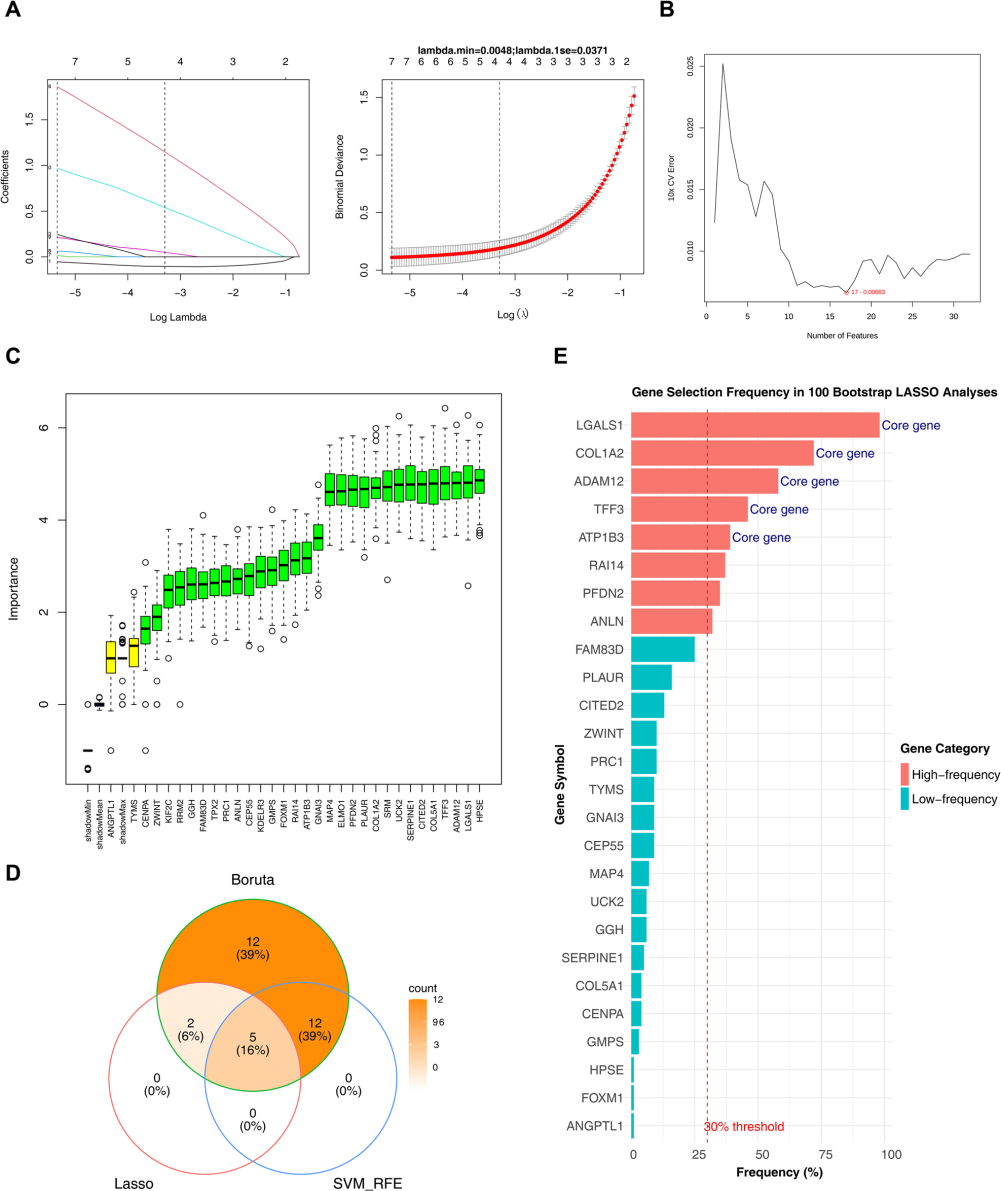
Machine learning for biomarker screening. **A** Feature genes screened by LASSO regression analysis. Left panel: The abscissa was log(Lambda), and the ordinate was the coefficient of genes. Right panel: The abscissa was log(Lambda), and the ordinate represented the cross-validation error. **B** Graph of generalization error versus the number of features. The minimum error value was in red font. **C** Gene interpretation diagram of the Boruta model. **D** Venn diagram of the intersection of three machine learning algorithms. **E** Gene selection frequency in 100 bootstrap LASSO analyses.

To identify signature genes, candidate genes were analyzed using multiple machine learning algorithms. The LASSO regression model results are presented in Fig. 2A. The SVM-RFE algorithm identified 17 signature genes with high classification accuracy, including LGALS1, ADAM12, PFDN2, GNAI3, ELMO1, COL1A2, FOXM1, ATP1B3, MAP4, RAI14, UCK2, COL5A1, CITED2, GMPS, HPSE, TFF3, and PLAUR (Fig. 2B). The Boruta algorithm identified 31 signature genes (Fig. 2C). By intersecting the results of these machine learning approaches, five biomarkers—*ATP1B3, TFF3, LGALS1, ADAM12*, and *COL1A2*—were selected (Fig. 2D). To validate the robustness of feature selection, we performed 100 bootstrap LASSO resamplings on the candidate genes. The results showed that eight genes exceeded the 30% selection frequency threshold. Importantly, all five of our identified core biomarkers—*LGALS1* (98%), *COL1A2* (72%), *ADAM12* (58%), *TFF3* (46%), and *ATP1B3* (39%)—ranked among the top five high-frequency genes, with selection frequencies significantly higher than those of other candidate genes (e.g., RAI14: 37%) (Fig. 2E). These findings fully demonstrate the importance and stability of the five core biomarkers in the model.

### The expression analysis and clinical sample validation of biomarkers

**Fig. 3:**
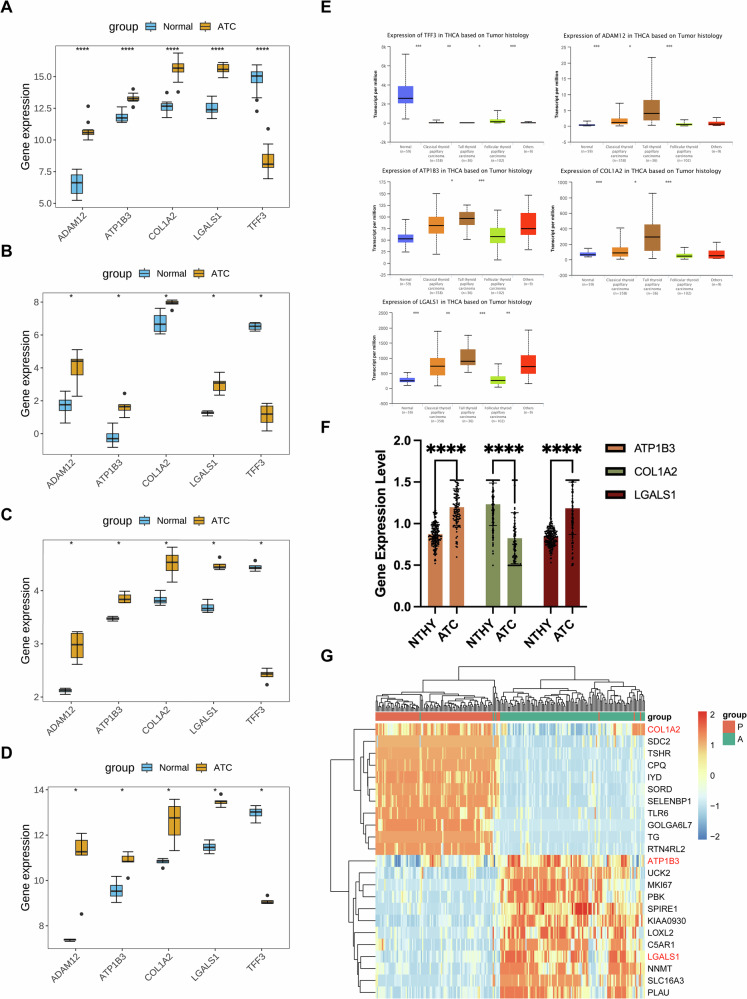
Expression analysis and validation of biomarkers. **A** The expression analysis of biomarkers in the GSE65144 dataset. **** represented *P* < 0.001. **B** The expression analysis of biomarkers in the GSE9115 dataset. * represented *P* < 0.05. **C** The expression analysis of biomarkers in the GSE27155 dataset. * represented *P* < 0.05. **D** The expression analysis of biomarkers in the GSE53072 dataset. * represented *P* < 0.05. **E** The expression levels of biomarkers in other THCA samples. * represented *P* < 0.05, ** represented *P* < 0.01, and *** represented *P* < 0.001. **F**, **G** Changes in expression compared to paraneoplastic tissue. *** represented *P* < 0.001.

The expression patterns of these biomarkers were analyzed in the GSE65144 and GSE9115 datasets, demonstrating upregulation of *ATP1B3, LGALS1, ADAM12*, and *COL1A2* in ATC, whereas *TFF3* was downregulated (Fig. 3A, B). Validation in the GSE27155 and GSE53072 datasets confirmed that biomarker expression trends were consistent with findings from the training datasets, with significant differences observed between groups (Fig. 3C, D). Further analysis using the UALCAN database indicated significant expression differences in other thyroid cancers (Fig. 3E).

After completing these experiments, we analysed the available clinical samples (The data of proteomics have been uploaded in the ProteomeXchange Consortium as reported previously (iProX database, project ID: IPX0008941000))(including 99 ATC paracancerous tissues and 115 ATC tissue samples), and analysed three genes (ATP1B3, COL1A2 and LGALS1) with certain significance. Therefore, we carried out the next step of expression measurement for the three genes (Fig. 3F), in which it is obvious that ATP1B3 and LGALS1 were significantly elevated in ATC compared to normal paracancerous thyroid tissues (NTHY), and the trend of ATP1B3 was more obvious, but the trend of COL1A2 was indeed a downward trend. The heatmap also reflects a view of the appeal (Fig. 3G).

### Nomogram model had high prediction accuracy for ATC

**Fig. 4:**
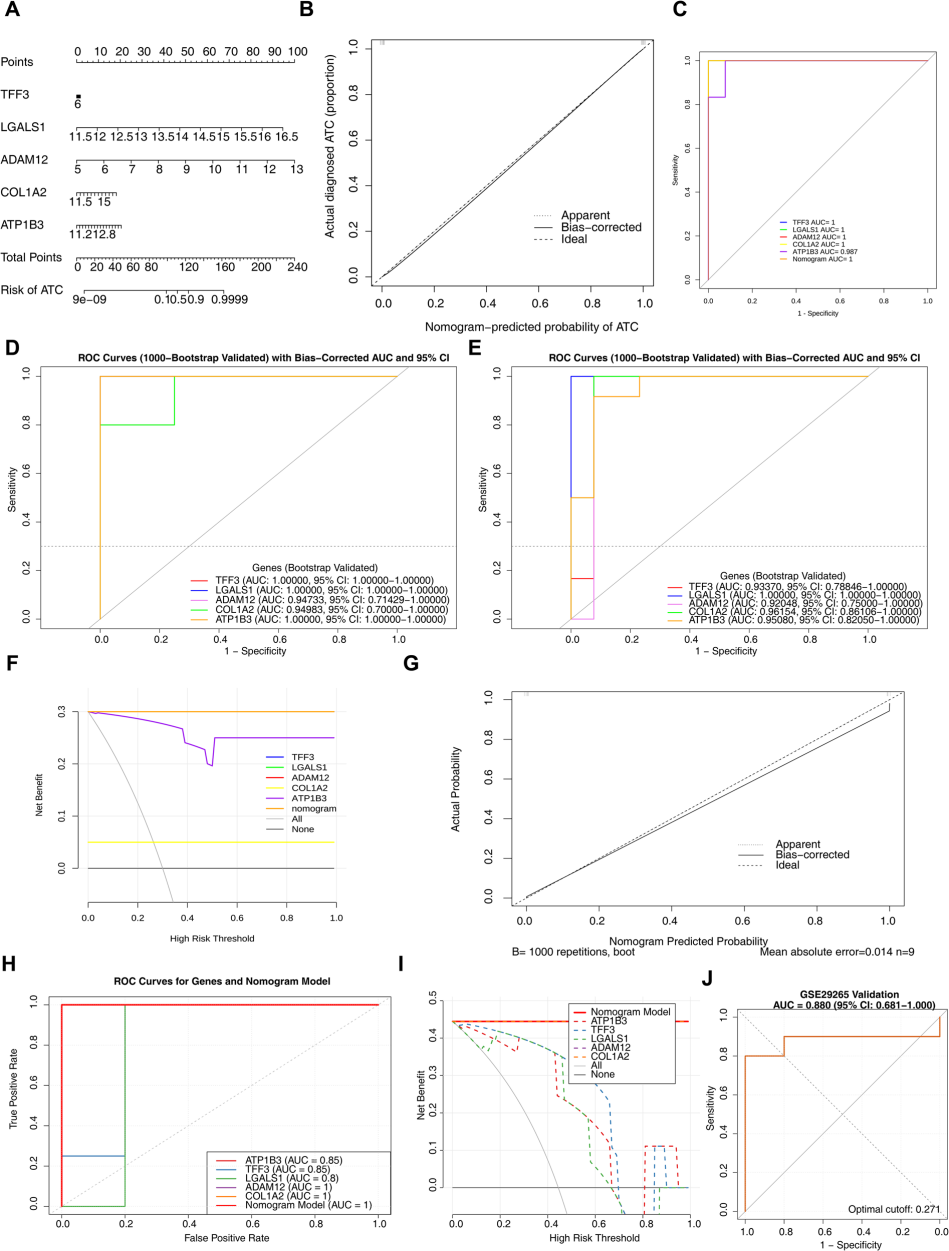
Construction and validation of the nomogram. **A** Construction of the nomogram model. **B** Calibration curve of the nomogram. **C** ROC curves of the nomogram and biomarkers. **D** Bootstrap validation of the GSE9115 dataset. **E** Bootstrap validation of the GSE65144 dataset. **F** Decision curve analysis of the nomogram and biomarkers. **G** Calibration curve in the GSE9115 dataset. **H** ROC curves of the nomogram and biomarkers in the GSE9115 dataset. **I** Decision curve analysis in the GSE9115 dataset. **J** The ROC curve of the GSE29265 dataset.

A nomogram model incorporating ATP1B3, TFF3, LGALS1, ADAM12, and COL1A2 was constructed to predict ATC occurrence (Fig. 4A). Calibration curves indicated excellent predictive accuracy of the model (Fig. 4B). The ROC curve analysis revealed an AUC of 1.0 for the nomogram model, an AUC of 1.0 for four biomarkers (TFF3, LGALS1, ADAM12, and COL1A2), and an AUC of 0.987 for ATP1B3 (Fig. 4C). To rigorously address potential concerns of overfitting and to evaluate the model’s generalizability, we performed an internal validation using the bootstrap method with 1000 resamples on both the GSE65144 and GSE9115 datasets. The results demonstrated that in the GSE9115 dataset, the bias-corrected AUCs for TFF3, LGALS1, and ATP1B3 were all 1.000 (95% CI: 1.000–1.000), with effective sampling rates all exceeding 0.99, confirming that their perfect classification performance was not attributable to small-sample artifacts. The corrected AUCs for ADAM12 and COL1A2 also remained stable in the range of 0.947–0.949 (Fig. 4D). In the GSE65144 dataset, LGALS1 similarly maintained an AUC of 1.000, while the corrected AUCs for the other biomarkers (ranging from 0.921 to 0.962) were highly consistent with the original values (Fig. 4E). Decision curve analysis demonstrated a strong likelihood of clinical benefit for both individual biomarkers and the nomogram model, with overlapping curves for TFF3, LGALS1, ADAM12, and the overall nomogram model (Fig. 4F). The nomogram model was validated in the GSE9115 dataset (Fig. 4G–I), and the corrected C-index was 0.95, indicating strong predictive ability of the nomogram. The ROC curves of each biomarker and the nomogram model all exceeded 0.8, suggesting high diagnostic accuracy. The decision curve analysis showed that both single genes and the overall model offered substantial clinical benefits, with the curves of COL1A2, ADAM12, and the Nomogram Model overlapping. Furthermore, the external validation conducted using the GSE29265 dataset demonstrated that the AUC value derived from the ROC curve analysis indicated its excellent discriminatory ability (Fig. 4J).

### Functional enrichment of candidate genes and biomarkers

To further explore the functional relevance of the biomarkers, GSEA was performed. Single-gene GO enrichment analysis demonstrated that most of the five biomarkers were significantly associated with biological processes such as collagen fibril organization, chromosome segregation, and sister chromatid segregation. KEGG analysis identified the most significantly enriched pathways as ECM-receptor interaction, cell cycle regulation, and focal adhesion. The top five GO terms and KEGG pathways are displayed in Supplementary Fig. 3A–J.

### Biomarkers were associated with infiltrating cells

**Fig. 5:**
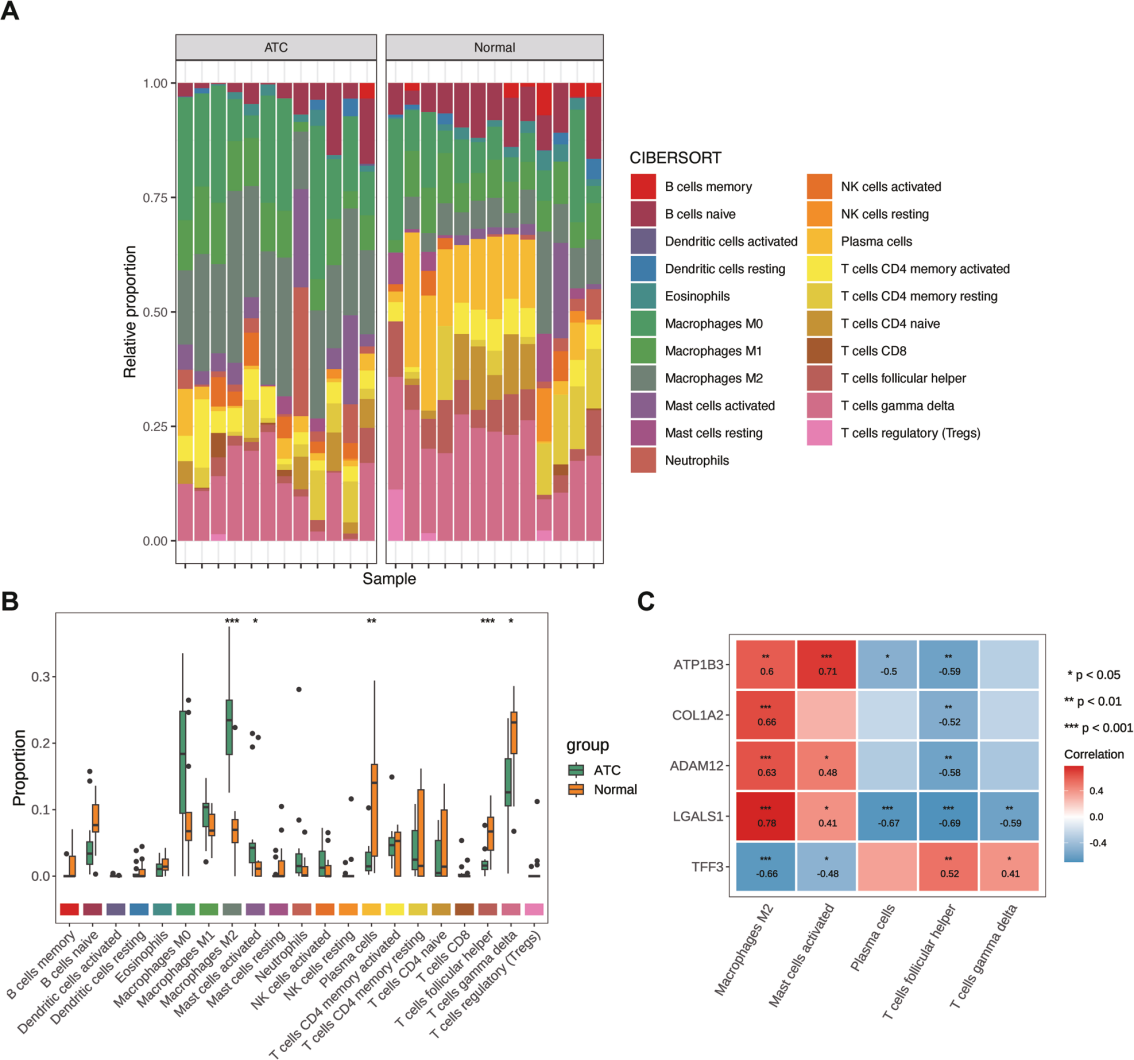
Immune infiltration analysis. **A** Distribution map of immune cell types. **B** Boxplot of differentially abundant immune cells between disease and control groups. * indicated *P* < 0.05, ** indicated *P* < 0.01, and *** indicated *P* < 0.001. **C** Correlation between biomarkers and differentially abundant immune cells. Red represented positive correlation, blue represented negative correlation, and darker colors indicated stronger correlation; * indicated *P* < 0.05, ** indicated *P* < 0.01, and *** indicated *P* < 0.001.

Using the CIBERSORT algorithm, the distribution of 22 immune cell types in normal and ATC tissue samples from the GSE65144 dataset was analyzed (Fig. 5A). Five immune cell types—follicular helper T cells, M2 macrophages, plasma cells, activated mast cells, and gamma delta T cells—exhibited significant differences in abundance between ATC and normal tissues (Fig. 5B), suggesting a potential role in ATC progression. Correlation analysis revealed strong associations between differentially abundant immune cells and biomarkers (Fig. 5C). M2 macrophages exhibited positive correlations with ATP1B3, LGALS1, ADAM12, and COL1A2 but were negatively correlated with TFF3. Conversely, follicular helper T cells demonstrated a negative correlation with ATP1B3, LGALS1, ADAM12, and COL1A2 while being positively correlated with TFF3.

### Two subtypes were identified through consensus clustering

**Fig. 6:**
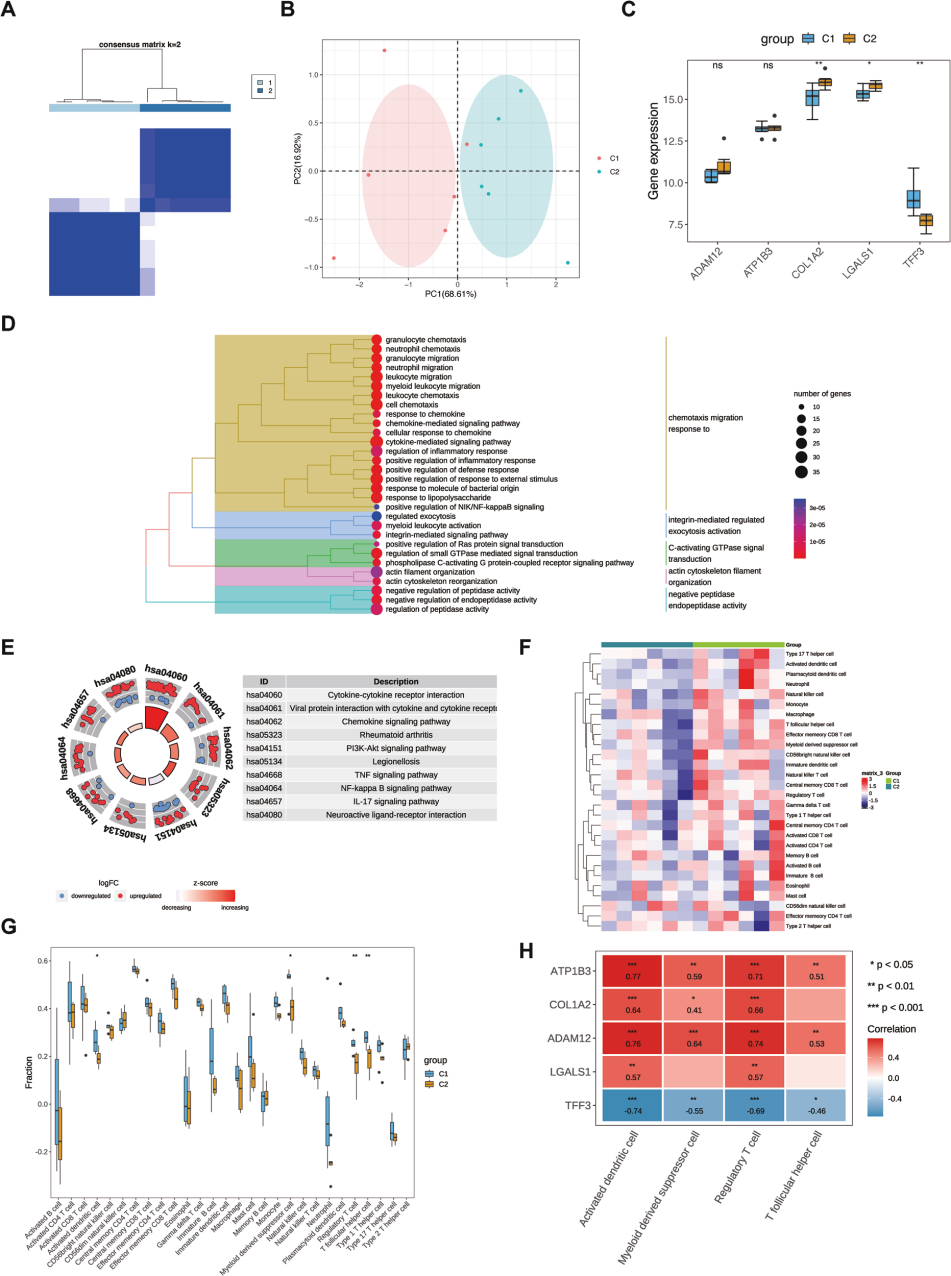
Subtype analysis of cancer samples in ATC. **A** The cumulative distribution function (CDF) curve of consensus clustering. The x-axis represents the consensus index, which reflects the clustering consistency between samples, while the y-axis represents the CDF value, which reflects the stability of the clustering results. Different colored curves corresponded to different numbers of clusters. **B** The PCA plot of different subtypes. Subtype 1 was colored red, and subtype 2 was green. **C** Expression differences of biomarkers between subtypes. ns indicated no significance, * represented *P* < 0.05, and ** represented *P* < 0.01. **D** GO enrichment results of differentially expressed genes. **E** KEGG enrichment results of differentially expressed genes. **F** Heatmap of immune cell contents. **G** Boxplots of immune cell differences between groups. * represented *P* < 0.05, and ** represented *P* < 0.01. **H** Correlation between biomarkers and immune cell contents. Red indicated positive correlation, blue indicated negative correlation, and darker colors represented stronger correlation. * represented *P* < 0.05, ** represented *P* < 0.01, and *** represented *P* < 0.001.

Consensus clustering analysis indicated that the CDF plot stabilized when the 12 ATC samples from the GSE65144 dataset were categorized into two distinct subtypes (Fig. 6A). PCA confirmed that the two subtypes (C1 and C2) were well separated (Fig. 6B). Differential expression analysis of biomarkers between the subtypes revealed significant differences in LGALS1, TFF3, and COL1A2 expression, whereas ATP1B3 and ADAM12 showed no significant variation (Fig. 6C).

A total of 626 DEGs were identified between the two subtypes, consisting of 365 upregulated and 261 downregulated genes. GO and KEGG enrichment analyses demonstrated that these DEGs were predominantly involved in neutrophil migration, granulocyte chemotaxis, and myeloid leukocyte migration (Fig. 6D), as well as classical signaling pathways such as NF-kappa B and PI3K-Akt (Fig. 6E). Analysis of 28 immune cell types in the C1 and C2 subtypes of the GSE65144 dataset revealed significant differences in four immune cell types: activated dendritic cells, follicular helper T cells, regulatory T cells, and myeloid-derived suppressor cells (Fig. 6F). The Wilcoxon test indicated statistically significant differences in these immune cell populations between subtypes C1 and C2 (Fig. 6G). Correlation analysis further confirmed that ATP1B3, LGALS1, ADAM12, and COL1A2 were positively associated with activated dendritic cells and regulatory T cells, whereas TFF3 was negatively correlated with these immune cells (Fig. 6H).

### ATP1B3 knockdown may inhibit malignant behaviour of ATC cells by triggering disulfidptosis

**Fig. 7:**
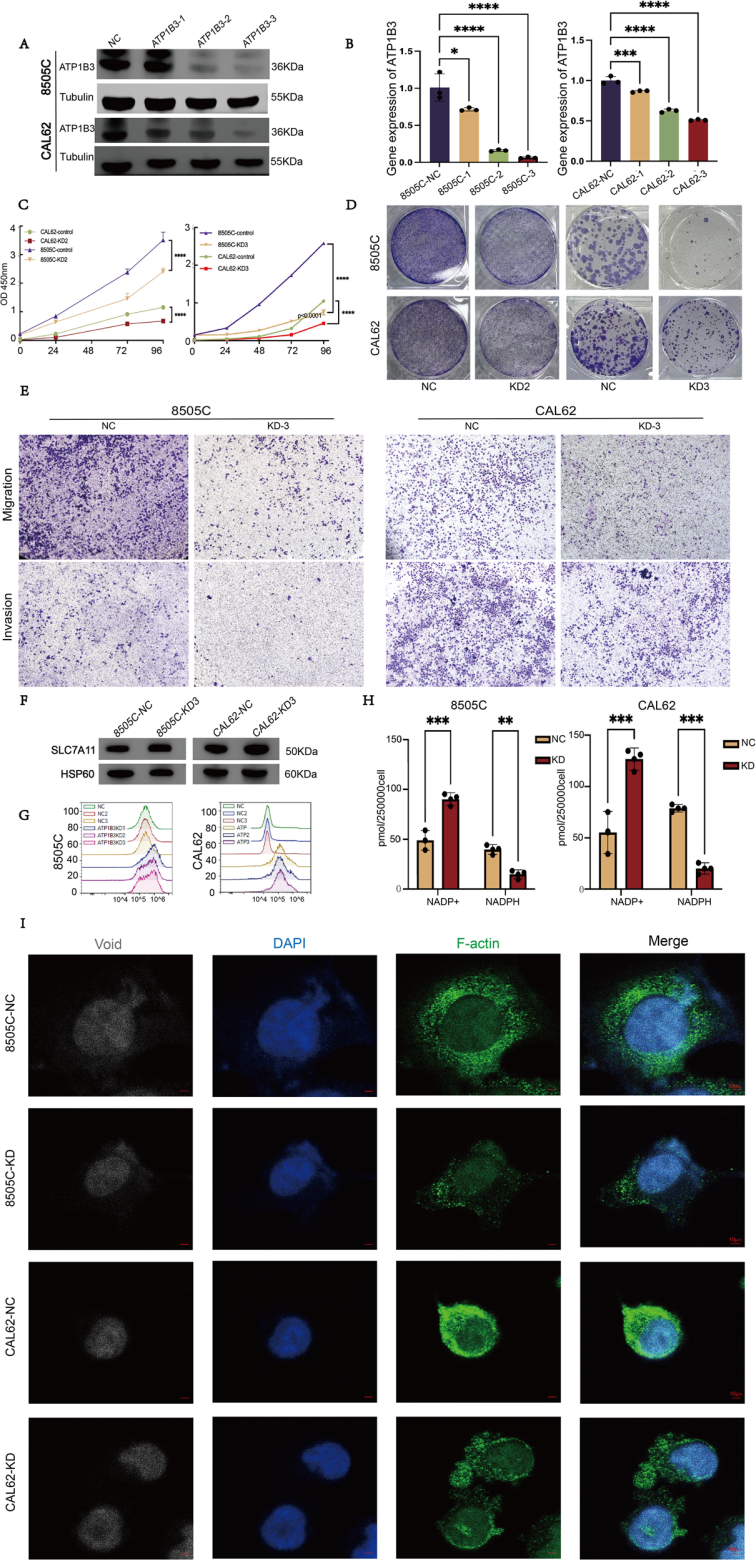
ATP1B3 knockdown may inhibit malignant behaviour of ATC cells by triggering disulfidptosis. **A**, **B** WB and PCR validation of knockdown efficiency. **C** Value-added curves of the two cell lines. **D** Colony formation ability. **E** Changes in migratory invasive ability of the two cell lines. **F** Expression of SLC7A11 in the control and the knockdown groups. **G** Flow cytometry to verify the degree of cell death. **H** Changes in NADP + /NADPH content. **I** Observation of the cell morphology by laser confocal microscopy changes.

As a key gene in the diagnostic model, ATP1B3 was selected for further validation through cellular and animal experiments. Two ATC cell lines, 8505C and CAL62, were used for these studies. Stable ATP1B3 knockdown was achieved via viral transfection, and knockdown efficiency was confirmed by Western blot (WB) and PCR (Fig. 7A, B). Sequences 2 and 3 demonstrated higher knockdown efficiency and were therefore selected for subsequent preliminary functional experiments.

**Fig. 8:**
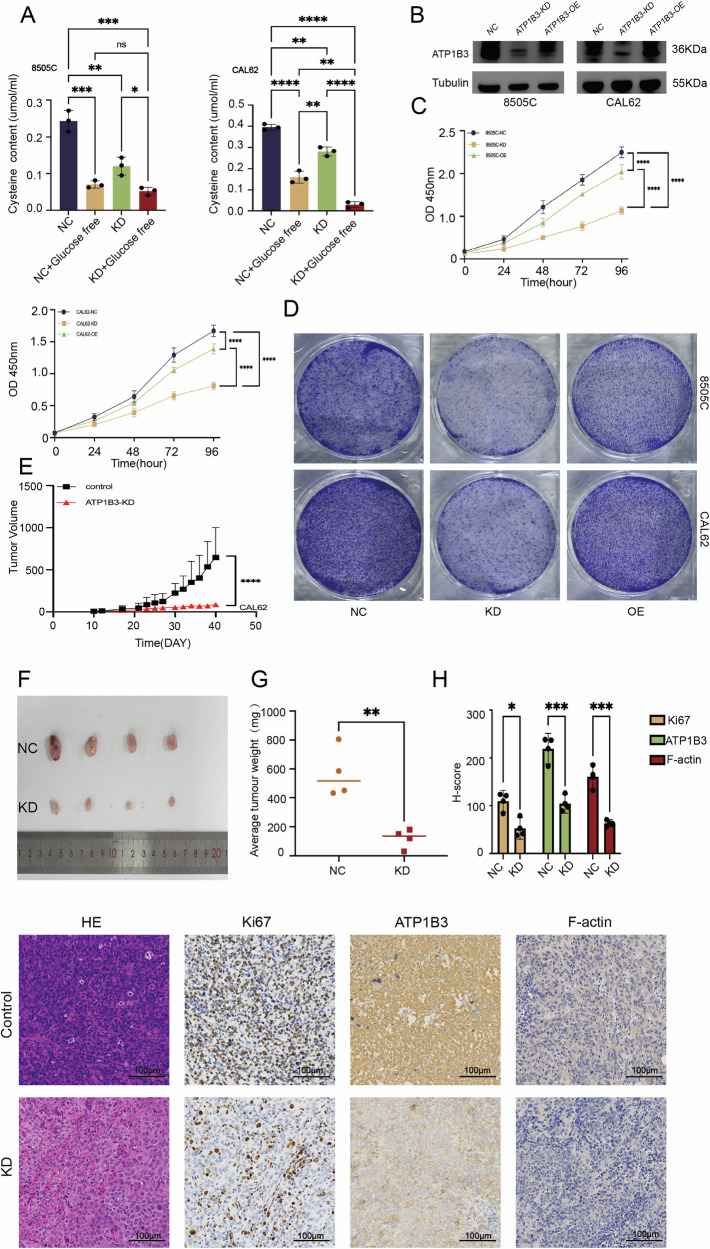
Consistent in vivo tumour growth after ATP1B3 knockdown. **A** Changes in cystine content. **B** Overexpression Efficiency Validation. **C** Proliferation curves of two cell groups. **D** Ability to form colonies. **E**, **F** Tumour growth was significantly inhibited after knockdown. **G** Tumour weight was also significantly reduced in the knockdown group. **H** The results of immunohistochemistry also further verified the inhibitory effect on tumours after ATP1B3 gene knockdown. Concurrently, H-score was performed on the sections.

Functional assays revealed that ATP1B3 knockdown significantly suppressed proliferation and colony formation (Fig. 7C, D). Sequence 3 demonstrated a more pronounced inhibitory effect than Sequence 2; consequently, all subsequent experiments employed Sequence 3 for further investigation. Moreover, migration and invasion capabilities were markedly reduced in the ATP1B3 knockdown group compared to the control group (Fig. 7E), suggesting that ATP1B3 plays a critical role in maintaining ATC cell viability. Given that disulfidptosis is often associated with elevated expression of SLC7A11 (XCT), SLC7A11 expression was examined in both cell lines. WB analysis confirmed an increase in SLC7A11 expression in ATP1B3 knockdown cells compared to controls (Fig. 7F). Flow cytometry demonstrated a significant increase in cell death following ATP1B3 knockdown (Fig. 7G). Furthermore, NADP + /NADPH assay results revealed significantly increased NADPH consumption in the knockdown group. For the 8505 C cell line, the average NADPH concentration in the control group was 39.640 pmol/250,000 cells, whereas the knockdown group exhibited an average concentration of 14.763 pmol/250,000 cells. In the CAL62 cell line, the average NADPH concentration in the NC group was 78.703 pmol/250,000 cells, while the average NADPH concentration in the knockdown group was 20.108 pmol/250,000 cells (Fig. 7H), consistent with previous research findings. Subsequently, we employed confocal microscopy to observe that F-actin exhibited marked contraction in ATP1B3-knockdown cells, further corroborating its role in disulfide-bond apoptosis (Fig. 7I). Finally, using a cysteine assay kit, we observed a marked reduction in cellular cysteine levels following ATP1B3 gene knockdown compared to the control group. The decrease in NADPH content prevented the reduction of cystine to cysteine within the cells (Fig. 8A).

### Rescue trial

Simultaneously, we procured overexpression plasmids for the target gene. Cells exhibiting favourable post-transfection morphology were selected for plating. Following complete cell confluence, further transfection experiments were conducted using Lipofectamine 3000 as the primary transfection reagent. Cultures were maintained in 1640 medium supplemented with double FBS. Upon reaching the requisite cell proliferation levels, Western blot (Fig. 8B) assays were promptly performed to validate overexpression efficiency. Having confirmed overexpression efficiency, we proceeded with selected phenotypic assays: CCK8 proliferation assays (5000 cells per well) (Fig. 8C) and colony formation assays (10,000 cells per well, fixed and stained immediately upon reaching the required proliferation level) (Fig. 8D). These tests further corroborated the impact of ATP1B3 knockdown on cellular growth and proliferation.

### Consistent in vivo tumor growth after ATP1B3 knockdown

To confirm the in vitro findings, a xenograft mouse model was established to evaluate the effect of ATP1B3 knockdown on tumor growth. Tumor growth was significantly suppressed in the ATP1B3 knockdown group compared to the control group (Fig. 8E, F). Consistent with tumor volume measurements, ATP1B3 knockdown also resulted in a significant reduction in tumor weight (Fig. 8G), highlighting its strong anti-tumorigenic effect. Immunohistochemistry analysis further confirmed decreased expression levels of HE, Ki67, and ATP1B3 in ATP1B3 knockdown tumors, reinforcing its potential role in ATC suppression. The reduction of F-actin also confirms the existence of a greater or lesser relationship with disulfide death in a sideways manner (Fig. 8H). ATP1B3 expression was significantly reduced in the ATP1B3 knockout group (*P* < 0.01), as was Ki67 expression (*P* < 0.05), further confirming the inhibitory effect of ATP1B3 knockout on tumour growth (Fig. 8H).

## Discussion

ATC is a rare but highly aggressive malignancy, accounting for only 1–2% of all thyroid cancers but contributing to the majority of thyroid cancer-related deaths. The five-year survival rate is a mere 7%, with a median survival time of approximately five months. Despite extensive research efforts, no significant advancements have been made in improving the overall survival of ATC over the past three decades.

Disulfidptosis, a novel mode of cell death first described in 2023 by Prof. Gan Poyi et al., is triggered by SLC7A11-mediated disulfide stress due to intracellular cystine accumulation. This form of cell death is characterized by excessive disulfide bond formation between actin cytoskeletal proteins under glucose deprivation in cells with high SLC7A11 expression. These aberrant disulfide bonds cause actin filament contraction, detachment from the plasma membrane, cytoskeletal collapse, and ultimately cell death. By intersecting differentially expressed genes with those related to both disulfidptosis and ATC, five key genes were identified and used to construct a molecular diagnostic model that demonstrated high predictive accuracy for ATC [6].

Among the identified biomarkers, ATP1B3 was previously reported to be significantly upregulated in hepatocellular carcinoma (HCC) and associated with immune cell infiltration and immune-related cytokine expression [8]. In estrogen receptor (ER)-positive breast cancer, ATP1B3 overexpression has also been linked to increased cisplatin sensitivity, suggesting its value as both a prognostic biomarker and a potential therapeutic target in HCC and ER-positive breast cancer [9, 10]. In this study, ATP1B3 knockdown in ATC cells significantly inhibited proliferation, and its high expression correlated with poorer prognosis, highlighting its potential as a biomarker for prognosis assessment and therapeutic intervention in ATC.

LGALS1 (Galectin-1), a β-galactoside-binding lectin, is overexpressed in non-small cell lung cancer (NSCLC) and promotes NSCLC cell proliferation, migration, and invasion through the NCAPG/LGALS1/SPARC axis [11, 12]. High expression of LGALS1 has also been associated with adverse outcomes in ovarian and oral cancers [13, 14]. Although its exact role in ATC remains undefined, its known pro-metastatic and immune-regulatory functions suggest that LGALS1 may enhance ATC cell invasiveness and facilitate immune evasion, thereby contributing to tumor progression. Further investigation is warranted to elucidate its precise mechanisms in ATC and assess its potential as a therapeutic target.

TFF3 (Trefoil factor 3), a member of the trefoil factor family, is upregulated in various malignancies, including breast, gastric, colorectal, thyroid, and cervical cancers. Its oncogenic effects are thought to be mediated through the MAPK/ERK pathway, promoting tumor proliferation and metastasis [15]. Notably, TFF3 polymorphisms have been linked to susceptibility to papillary thyroid carcinoma [16]. Consistent with our findings, Li et al. also identified TFF3 as a diagnostic biomarker for ATC with robust diagnostic performance [17]. These data suggest that TFF3 may play a critical role in ATC pathogenesis and serve as a promising diagnostic and therapeutic target.

COL1A2, which encodes type I collagen, has been implicated in tumor differentiation. Its inhibition suppresses glioma cell proliferation and invasion [18]. Interestingly, while COL1A2 is upregulated in gastric and epithelial ovarian cancers [19, 20], it exhibits tumor-suppressive functions in bladder cancer [21] and head and neck cancers [22]. This dual role suggests a context-dependent function, and further investigation is needed to clarify its mechanistic role in ATC and assess its therapeutic potential.

ADAM12, a member of the ADAM (a disintegrin and metalloproteinase) family, contributes to tumor development and progression in renal clear cell carcinoma [23], breast cancer [24], and hepatocellular carcinoma [25]. It promotes renal carcinoma progression via the EGFR/ERK signaling pathway [23] and has been proposed as a diagnostic biomarker for hepatocellular carcinoma [25]. In breast cancer, ADAM12 (that is regulated by TWIST1) enhances invasion and metastasis through pseudopodia and focal adhesion formation [26]. These findings support the hypothesis that ADAM12 may play a similar role in ATC, particularly in promoting metastasis.

To further investigate the functions of these biomarkers, enrichment analysis indicated significant associations with biological processes such as nucleoside monophosphate biosynthesis during mitosis, mitotic nuclear division, and microtubule cytoskeleton organization. KEGG pathway analysis revealed enrichment in nucleotide metabolism, axon guidance, and the p53 signaling pathway, consistent with previous studies and offering insights for future exploration. To experimentally validate these findings, ATP1B3 was selected for in vitro functional studies. Western blot and PCR analyses confirmed that ATP1B3 knockdown significantly inhibited ATC cell proliferation, colony formation, migration, and invasion. Moreover, Western blot showed increased XCT expression in ATP1B3-knockdown cells. Flow cytometry using PI staining revealed a significantly elevated apoptosis rate in the knockdown group. Confocal microscopy identified morphological alterations, including reduced cell size and nuclear wrinkling, indicating that ATP1B3 may be mechanistically linked to disulfidptosis in ATC cells.

While this study is the first to identify disulfidptosis-associated biomarkers in ATC, several limitations should be acknowledged. First, the marked heterogeneity of ATC may contribute to variability in gene expression profiles, and the relatively small sample size may limit statistical power and generalizability. Second, this study focused on a single ATC subtype, and its relevance to other thyroid cancer subtypes remains to be validated. Additionally, the absence of survival and clinical outcome data limits the ability to fully assess the translational potential of these biomarkers.To overcome these limitations, we shall expand our sample size and incorporate additional subtypes in future studies. By integrating multi-omics analyses with cellular and animal model experiments, we aim to systematically validate the functional mechanisms of these biomarkers. Specifically, we plan to conduct further rescue experiments in ATP1B3 knockdown models to clarify its causal role in ATC progression and disulfidptosis. Furthermore, quantitative assessment of total disulfide bond levels via DTNB assay will furnish the most direct biochemical evidence for ATP1B3’s regulation of disulfidptosis, ultimately elucidating its molecular mechanisms in ATC. Concurrently, extending observation periods in animal models and conducting long-term clinical follow-up will facilitate evaluation of biomarker-guided therapy efficacy and safety, thereby establishing robust preclinical and translational foundations for personalised ATC treatment.

## Materials and methods

### Data source

The four ATC-related datasets (GSE65144, GSE9115, GSE27155, and GSE53072) were obtained from the Gene Expression Omnibus (GEO) database (http://www.ncbi.nlm.nih.gov/geo/). The GSE65144 dataset contained 13 normal and 12 ATC thyroid tissue samples, while GSE9115 included 4 normal and 5 ATC thyroid tissue samples. Similarly, GSE27155 comprised 4 normal and 4 ATC thyroid tissue samples, and GSE53072 contained 4 normal and 5 ATC thyroid tissue samples. In addition, the GSE29265 dataset was also included for external validation, which contained 20 normal thyroid tissue samples and 29 ATC tissue samples. Additionally, 16 disulfidptosis-related genes (DRGs; Table S1) were retrieved from previously published literature [27].

### Differential expression analysis

Differential expression analysis was conducted to separately identify differentially expressed genes (DEGs) between ATC and normal samples in GSE65144 and GSE9115 using the ‘limma’ R package (version 3.52.4) [28], with the thresholds set at |Log_2_FC | > 1 and adj. *P* value < 0.05. Common DEGs were then determined using the Robust Rank Aggregation (RRA) method. The expression patterns of common DEGs were visualized using volcano and heat maps generated with the ggplot2 R package (version 3.3.2) [29] and the Pheatmap R package (version 1.0.12) [30].

### Identification of disulfidptosis- and ATC-related genes using Weighted Gene Co-expression Network Analysis (WGCNA)

Single-sample Gene Set Enrichment Analysis (GSVA) was performed to calculate the disulfidptosis score for each sample based on DRGs using the GSVA R package [28]. Co-expression networks were constructed and analyzed in the GSE65144 and GSE9115 datasets using the WGCNA R package. First, clustering analysis was conducted to identify potential outliers. A suitable soft threshold was selected based on the nearly scale-free topology criterion. The topological matrix was then clustered using differences between genes, and neighborhood and dissimilarity coefficients were calculated. Modules were identified using a dynamic tree-cutting method (minimum module size: 100 genes). Correlation analysis between modules and phenotypes (disulfidptosis score and ATC) was conducted, and modules with |cor | > 0.6 and *P* < 0.05 were designated as key modules. Genes within these modules were identified as disulfidptosis- and ATC-related genes.

### Identification of candidate genes

Common DEGs overlapping with disulfidptosis- and ATC-related genes were considered candidate genes. Chromosomal localization of these candidate genes was visualized using the RCircos R package (version 1.2.2) [31]. A protein-protein interaction (PPI) network was constructed using the STRING database (https://string-db.org/). Additionally, correlation analysis of candidate genes was performed using the ‘igraph’ R package (version 14.1), with a threshold of |R | > 0.7.

### Machine learning algorithms

Biomarkers were identified using three machine learning algorithms: least absolute shrinkage and selection operator (LASSO), support vector machine recursive feature elimination (SVM-RFE), and Boruta. Candidate genes were analyzed using the glmnet (Version 4.1-4) [32], e1071 (Version 1.7-13), and Boruta (Version 8.0.0) [33] packages, respectively. The feature genes identified by these algorithms were overlapped using the VennDiagram R package (Version 1.7.3) [34] to determine biomarkers.

The expression levels of biomarkers were evaluated in the training sets (GSE65144 and GSE9115) and validation sets (GSE27155 and GSE53072). Additionally, the expression levels of biomarkers in other thyroid cancers (THCA) were analyzed using the UALCAN database (http://ualcan.path.uab.edu). Finally, the prognostic value of biomarkers in THCA was assessed using survival analysis in the GEPIA database (http://gepia.cancer-pku.cn/). To evaluate the stability of the identified biomarkers, we performed bootstrap LASSO regression with 100 resamples on the candidate genes. The frequency of each gene being selected across all bootstrap samples was calculated.

### Construction of a nomogram model

To predict ATC occurrence, a nomogram model was constructed based on the identified biomarkers using the rms R package (Version 6.6-0) [32]. Calibration curves were plotted to evaluate the predictive accuracy of the nomogram model. Receiver Operating Characteristic (ROC) curves were generated for both the biomarkers and the nomogram model to assess diagnostic performance. Additionally, Decision Curve Analysis (DCA) was performed to determine the clinical utility of the nomogram model. Subsequently, the nomogram model was validated in the GSE9115 dataset. To assess the robustness and potential overfitting of the nomogram model, we performed internal validation using the Bootstrap method with 1000 resamples. The bias-corrected 95% confidence intervals (CIs) for the area under the receiver operating characteristic curve (AUC) were calculated to evaluate the model’s discriminative ability. To further validate the robustness and generalizability of the nomogram model, external validation was performed using the GSE29265 dataset.

### Enrichment analysis

To explore the biological functions of candidate genes and biomarkers, enrichment analysis was conducted. Gene Ontology (GO) and Kyoto Encyclopedia of Genes and Genomes (KEGG) pathway enrichment analyses were performed using the clusterProfiler R package (version 4.4.4) [35]. The adjusted *P* value < 0.05 was set as the cut-off criteri [36, 37]. Furthermore, Gene Set Enrichment Analysis (GSEA) was conducted to investigate the functional roles of biomarkers, with thresholds set at |NES | > 1 and *P* < 0.05.

### Immune infiltration analysis

Immune cell infiltration analysis was performed using the CIBERSORT algorithm [38] on ATC and normal tissue gene expression data from the GSE65144 dataset. Significantly different immune cell populations between the two groups were identified using the Wilcoxon test. To investigate the relationship between differential immune cells and biomarkers, Spearman correlation analysis was conducted between significantly different immune cells and biomarker expression levels.

### Consensus clustering analysis

Consensus clustering is a widely applied unsupervised method for subtype classification. In this study, unsupervised clustering was performed on 12 ATC samples from the GSE65144 dataset based on the identified biomarkers using the ConsensusClusterPlus R package (Version 1.60.0) [39]. The optimal number of subtypes was determined by evaluating the cumulative distribution function (CDF) curves. To validate the classification accuracy, principal component analysis (PCA) was conducted to assess sample distribution across different subtypes. Subsequently, differential expression analysis of biomarkers between subtypes was performed, and DEGs were identified using the thresholds of *P* < 0.05 and |log_2_FC | > 1. Functional enrichment analysis of DEGs, including GO and KEGG pathway analysis, was conducted using the ClusterProfiler package. The extent of immune cell infiltration between subtypes in the GSE65144 dataset was quantified using the GSVA algorithm, and significant differences were determined using the Wilcoxon test. Finally, correlations between biomarkers and immune cells were analyzed using Spearman’s correlation test.

### Statistical analysis

All statistical analyses were conducted using R software. The Wilcoxon test was applied to compare scores between two groups, while correlation analysis was performed using Spearman’s test. A *P* value < 0.05 was considered statistically significant.

### Clinical sample selection

The procedures used in this study comply with the ethical standards of the Ethics Committee of Zhejiang Provincial People’s Hospital. Thyroid cancer samples and adjacent thyroid tissue were collected from Zhejiang Provincial People’s Hospital, Zhejiang Cancer Hospital, and Shaoyifu Hospital (January 2010 to January 2022). Each patient signed a written informed consent form agreeing to have their samples and clinical information used for research purposes, and the study protocol was approved by the Ethics Committee of Zhejiang Provincial People’s Hospital(Approval No: 202510151505000115513).

### Cell culture

ATC cell lines 8505c, and CAL62 were obtained from Procell (Wuhan, China), and the Shanghai Cell Bank of the Chinese Academy of Sciences, respectively. Cells were cultured in RPMI 1640 medium (Hyclone) supplemented with 10% fetal bovine serum and 1% penicillin-streptomycin under standard conditions (37 °C, 5% CO_2_). Once a stable state was achieved, cells were transfected with either negative control (NC) or ATP1B3 knockdown lentiviruses (Jikai Genetics, China). After 24 h of transfection, cells were transferred to 10-cm culture dishes for further incubation.

### Western blotting

Following transfection, cells were lysed in protein lysis buffer (Cell lysis buffer for Western and IP; Beyotime, China) containing protease inhibitor PMSF. Equal amounts of protein were separated via SDS-PAGE and transferred to a PVDF membrane. Protein expression was visualized using an enhanced chemiluminescence (ECL) detection kit (Fude Biological, China). The primary antibodies used were ATP1B3 (67554-1-Ig, Proteintech Group, USA), β-Tubulin pAb (Ac008, Abcam, USA), SLC7A11 (26846-1-AP, Proteintech Group, USA), and HSP60 (15282-1-AP, Proteintech Group, USA).

### Reverse transcription-polymerase chain reaction (RT-PCR)

Following transfection, RNA was extracted from both control and knockdown cell lines using an RNA Rapid Extraction Kit. Reverse transcription was performed using the PrimeScript RT Master Mix (Takara, Japan), and gene expression was analyzed via quantitative PCR (qPCR) using the Hieff^®^ qPCR SYBR Green Master Mix (Yeasen Biotechnology, China). 18S was used as an internal control, and gene expression changes were quantified using the 2^-ΔΔCt^ method. Primer sequences used for PCR are listed in Table 1.

**Table 1:** qRT-PCR primer sequences.

Primer	Sequences (5’ to 3’)
ATP1B3-F	CCAAAATACCGTGACCAGATTCC
ATP1B3-R	ACGAAGTTGGATCAGACCTACTG
18S-F	AGGCCCTGTAATTGGAATGAGTC
18S-R	GCTCCCAAGATCCAACTACGAG

### CCK8

The CCK-8 assay was employed to evaluate the proliferative capacity of ATC cells. Following validation via PCR and Western blot analysis, the more efficient knockdown sequences 2 and 3 were selected for subsequent experiments. 8505c and CAL62 cells were seeded at a density of 5000 cells per well in 96-well plates. CCK-8 reagent was added every 24 h, and cell viability was assessed by measuring the optical density (OD) at 450 nm. Each experiment was performed in triplicate.

### Cloning formation

Cell lines used for colony formation assays were the same as those used in previous experiments. Control and experimental groups were seeded in six-well plates at a density of 1000 cells per well. Cells were incubated until colony formation reached an optimal density. Colonies were fixed with 1 mL of paraformaldehyde for 20 min at room temperature, followed by staining with 500 µL of crystal violet for 15 min. After three washes with PBS, plates were air-dried and imaged.

### Transwell migration and invasion assay

Cell migration and invasion were assessed using Transwell plates (LABSELECT, Hefei, Anhui, 6.5 mm). ATC cell lines (8505c, CAL62) were seeded into the upper chamber at a density of 2–4 × 10⁴ cells per well in serum-free medium (200 μL), while 800 μL of medium containing serum was added to the lower chamber. After 24 or 48 h of incubation, cells that had migrated or invaded were fixed with 0.1% crystal violet for 30 min. The number of migrated cells was quantified under an M7000 microscope by selecting representative fields of view. The average number of migrated cells was calculated from five random fields across three independent experiments.

### Flow cytometry analysis

Apoptosis analysis was performed using flow cytometry. Control and experimental groups of 8505c and CAL62 cells were seeded in six-well plates at a density of 1 × 10⁵ cells per well. After reaching adherence, cells were detached using trypsin, centrifuged, and washed 2–3 times with PBS. Cells were stained with apoptosis detection kits using single propidium iodide (PI) staining. Samples were analyzed using a flow cytometer (Beckman Colter, Inc., Ireland), and data were processed using FlowJo software.

### Confocal laser scanning microscope

For confocal imaging, 8505c (30,000 cells/well) and CAL62 (50,000 cells/well) were seeded in 35 mm glass-bottom dishes with quadruple compartments. After cell adherence, cells were washed three times with PBS (5 min per wash). Cells were then fixed with 4% paraformaldehyde for 1 h, followed by incubation with 200 µL of primary antibody dilution for 40 min. After three PBS washes, 80 µL of F-actin (ab205, 1:1000) was applied overnight at 4 °C in a humidified, dark chamber. Subsequently, cells were washed with PBS and incubated with 200 µL of Alexa Fluor® 488-conjugated AffiniPure Goat Anti-Rabbit IgG H&L (1:1000) for 1 h in the dark. DAPI staining (200 µL) was applied for 5 min. Fluorescence images were captured using a TCS SP8 confocal microscope.

### NADP + /NADPH

NADP + /NADPH levels were measured in control and ATP1B3 knockdown groups. One million cell precipitates were collected from both groups for analysis. NADP + /NADPH levels were quantified using the Biyunfei NADP + /NADPH Assay Kit (WST-8 method) according to the manufacturer’s instructions. In accordance with the experimental protocol, a blank control group, a standard sample group, and an experimental group were established. A standard curve was plotted using the standard sample group. The absorbance values measured at 450 nm for the experimental group were substituted into the curve to calculate the corresponding NADPH concentrations. Data analysis and graphing were performed using Prism 10 software.

### Cysteine testing

Establish four groups: (NC group, NC group supplemented with glucose-free medium, ATP1B3 knockdown group, knockdown group supplemented with glucose-free medium). Following cell processing the previous day, seed 300,000 cells into each well of a 6-well plate. The following day, after cells have adhered, replace with the corresponding medium. Continue culture for 6–8 h before harvesting the cell pellet. Cysteine was subsequently extracted using the Cysteine Content Assay Kit (boxbio, Cat. No.: AKAM004M). Absorbance was measured at 600 nm using a microplate reader, and a curve was plotted against the generated standard curve to determine cysteine content. Data were analysed and graphically represented using Prism 10 software.

### Overexpression rescue assay

Following completion of the aforementioned experiments, cells exhibiting favourable condition were selected for plating at a density of 200,000 cells per well. After allowing the cells to adhere overnight, transfection with the overexpression plasmid (Shanghai Genechem Co., Ltd.) was performed. Once cells reached the desired proliferation level, they were promptly processed for Western blot analysis to validate gene overexpression efficiency. Upon confirmation of overexpression efficiency, baseline phenotypic assays were immediately conducted, including the CCK8 proliferation assay and colony formation assay.

### In vivo xenograft tumor model

Female nude mice (BALB/c, 3–5 weeks old) were purchased from Hangzhou Qizhen Experimental Animal Technology (Hangzhou, China) and maintained at the laboratory animal center at Zhejiang Provincial People’s Hospital (Approval No. IACUCA20230309001) according to the requirements of feeding. All mice were randomly assigned to different groups for the test. One week later, CAL62 control and ATP1B3 knockdown cell suspensions were injected subcutaneously into the right abdominal cavity of nude mice. Moreover, the volume (0.5 × (shortest diameter)2 × (longest diameter)) and body weight were recorded every other day. Once tumors reached the required experimental volume, mice were euthanized humanely, and tumors were excised, weighed, and fixed in formalin for further analysis.

### Immunohistochemistry

Paraffin-embedded tumor tissues were sectioned into 4-μm thick slices and subjected to deparaffinization and rehydration. Antigen retrieval was performed using citrate buffer (pH 6.0), followed by blocking with 3% bovine serum albumin for 30 min. Sections were incubated with primary antibodies at 25 °C for 1 h, followed by HRP-labeled secondary antibodies at room temperature for 30 min. Nuclei were counterstained with hematoxylin, and positive 3,3’-diaminobenzidine (DAB) staining was visualized as a brownish-yellow coloration. Images were captured using a fluorescence microscope (Konfoong Biotech, Ningbo, China). Concurrently, we performed further H-Score staining on immunohistochemical sections using Visiopharm software. The entire tissue area was selected for H-Score analysis of the target object. H-Score = ∑(pi × i) = (percentage of weakly stained cells × 1) + (percentage of moderately stained cells × 2) + (percentage of strongly stained cells × 3), where i denotes the positivity grade classification: negative (no staining) = 0 points; weakly positive (pale yellow) = 1 point; moderate positivity: brownish-yellow, scored 2 points; strong positivity: dark brown, scored 3 points. pi denotes the percentage of positive cells. The H-score ranges from 0 to 300, with higher values indicating greater overall positive intensity.

## Supplementary information

Supplementary Date

Supplementary Data

## Data Availability

The datasets used and/or analysis during the current study are available from the corresponding author on reasonable request.
