# Supplementary figures and images for: Identification and molecular typing of disulfidptosis-related biomarkers in anaplastic thyroid carcinoma
Source: Cell Death Discov. 2026 Apr 22;12:268. doi: 10.1038/s41420-026-03089-9 (PMC13237300; doi:10.1038/s41420-026-03089-9)

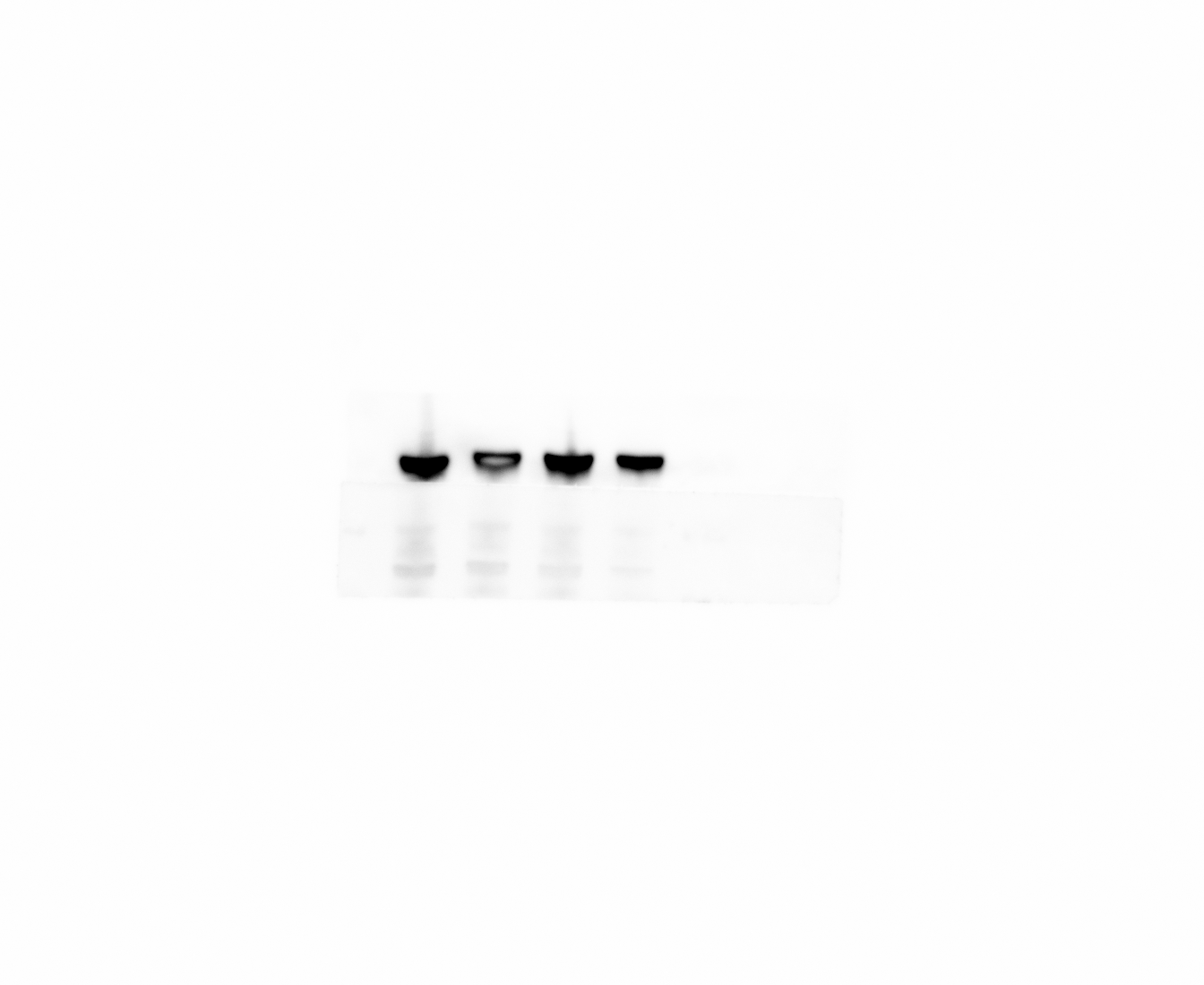

Supplement: Supplementary file 2 — Supplementary Data [file 41420_2026_3089_MOESM2_ESM.zip › Original Data File/Figure7-A Original Data/162-he_chemi.tif]

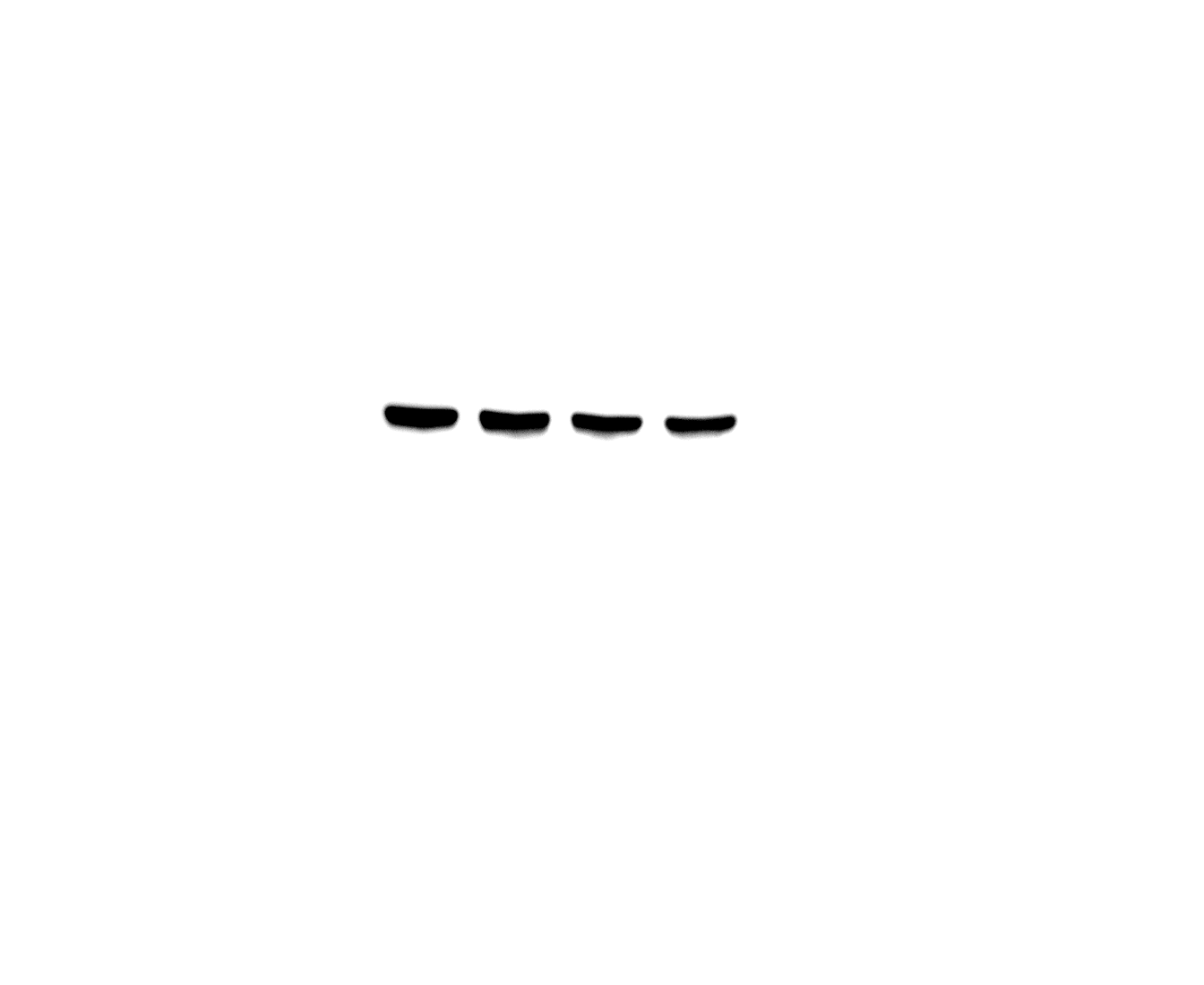

Supplement: Supplementary file 2 — Supplementary Data [file 41420_2026_3089_MOESM2_ESM.zip › Original Data File/Figure7-A Original Data/85-TUB_chemi.tif]

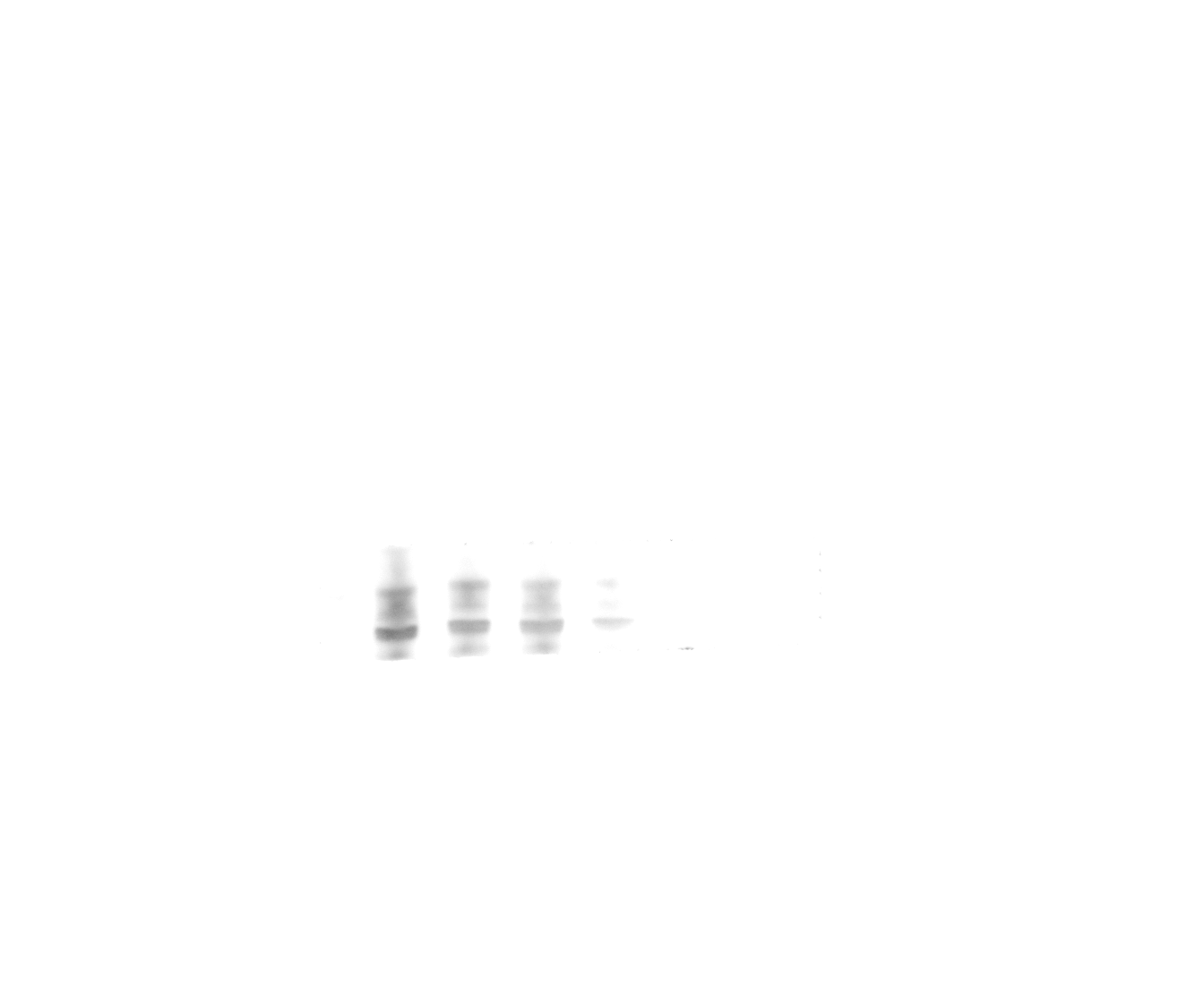

Supplement: Supplementary file 2 — Supplementary Data [file 41420_2026_3089_MOESM2_ESM.zip › Original Data File/Figure7-A Original Data/162-ATP_chemi.tif]

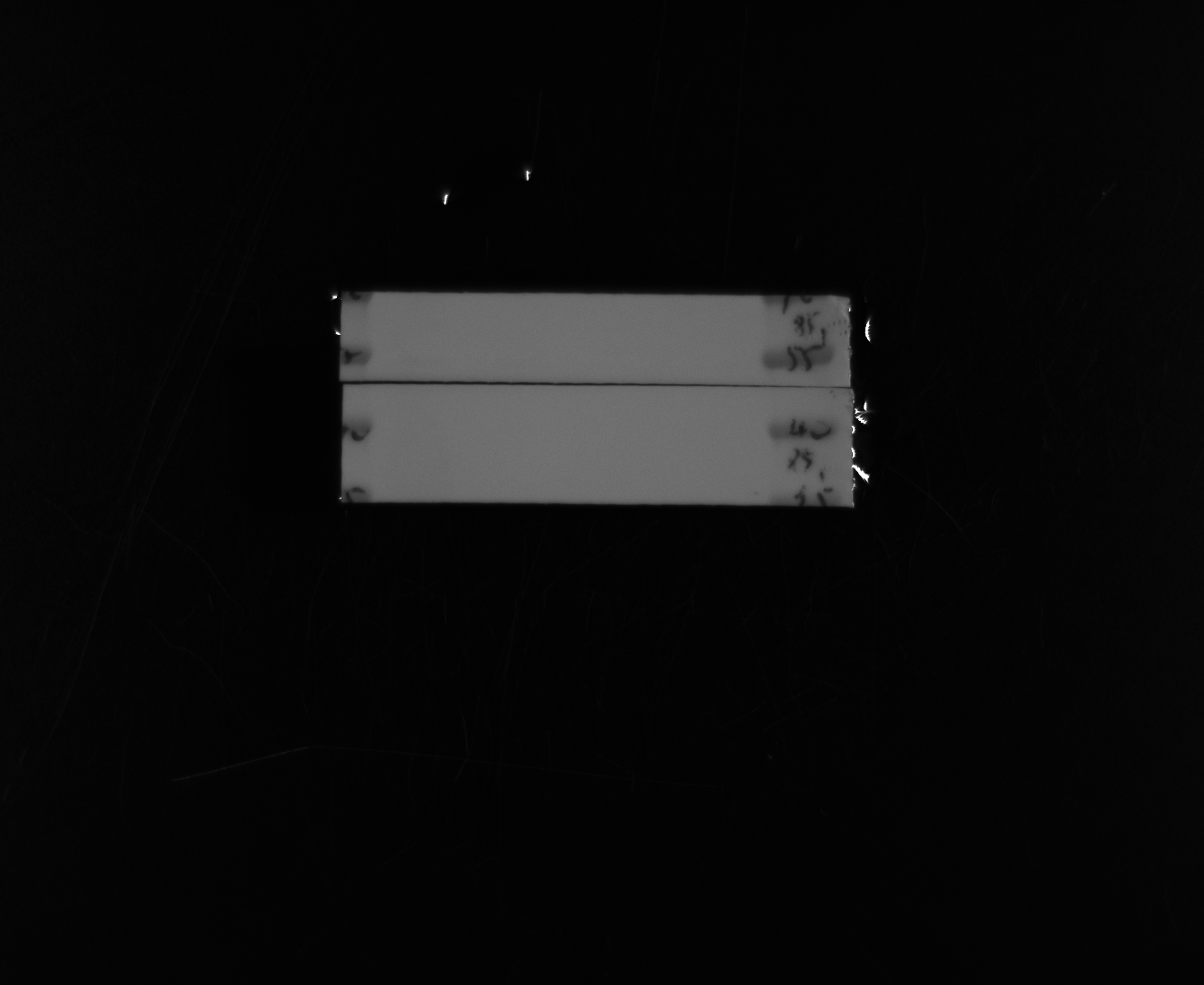

Supplement: Supplementary file 2 — Supplementary Data [file 41420_2026_3089_MOESM2_ESM.zip › Original Data File/Figure7-A Original Data/85-HE_marker.tif]

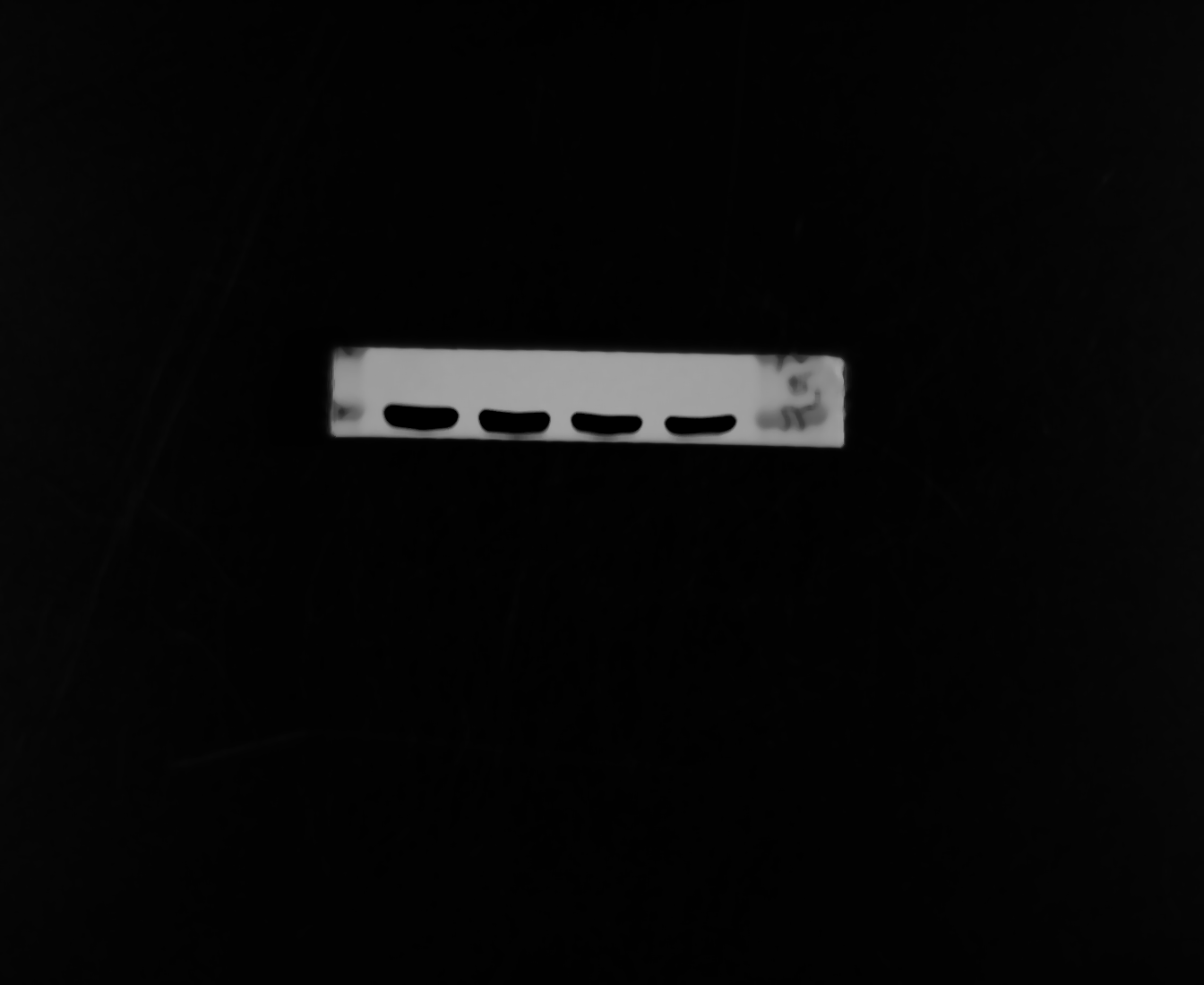

Supplement: Supplementary file 2 — Supplementary Data [file 41420_2026_3089_MOESM2_ESM.zip › Original Data File/Figure7-A Original Data/85-TUB.tif]

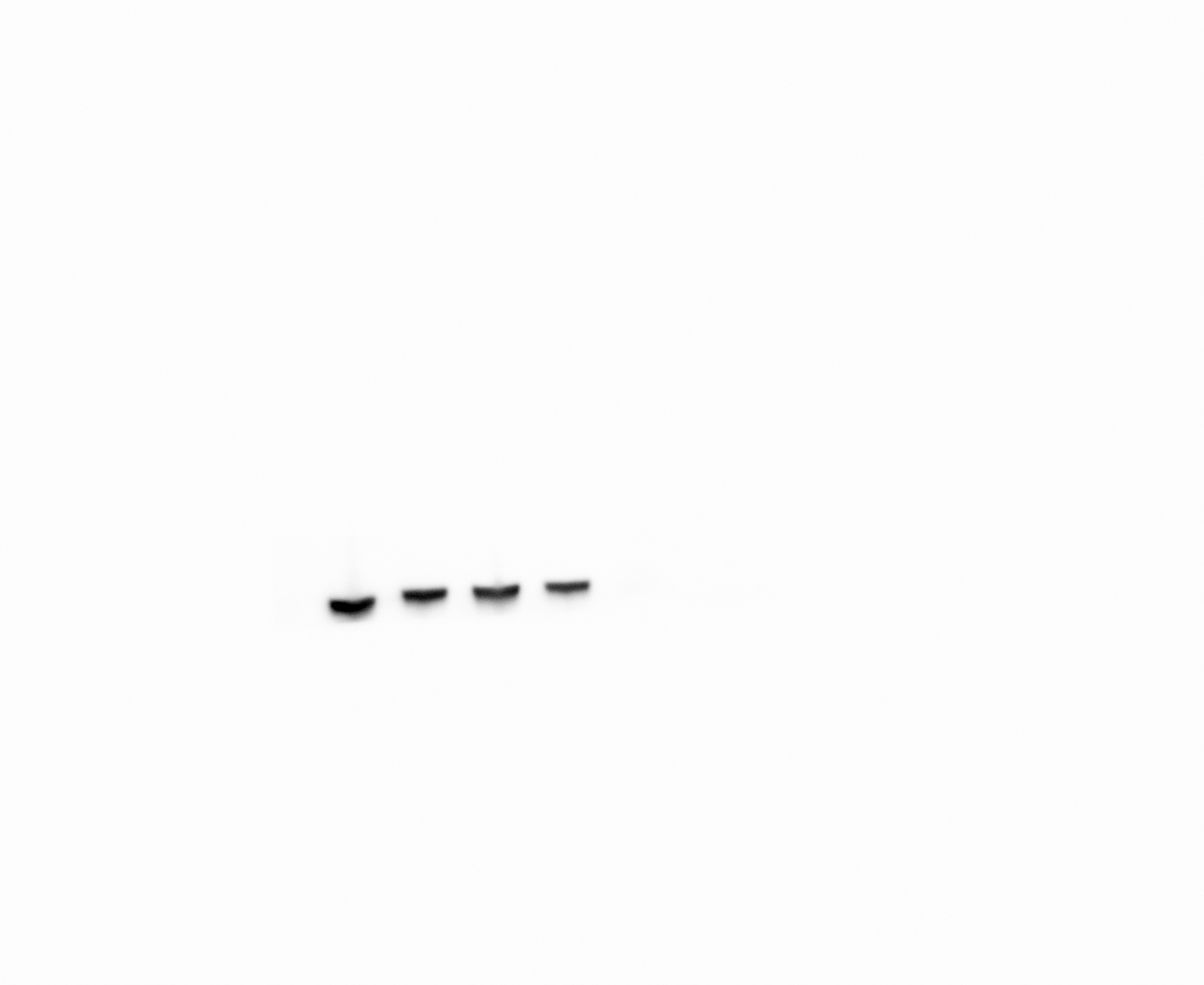

Supplement: Supplementary file 2 — Supplementary Data [file 41420_2026_3089_MOESM2_ESM.zip › Original Data File/Figure7-A Original Data/162-2-TUB_chemi.tif]

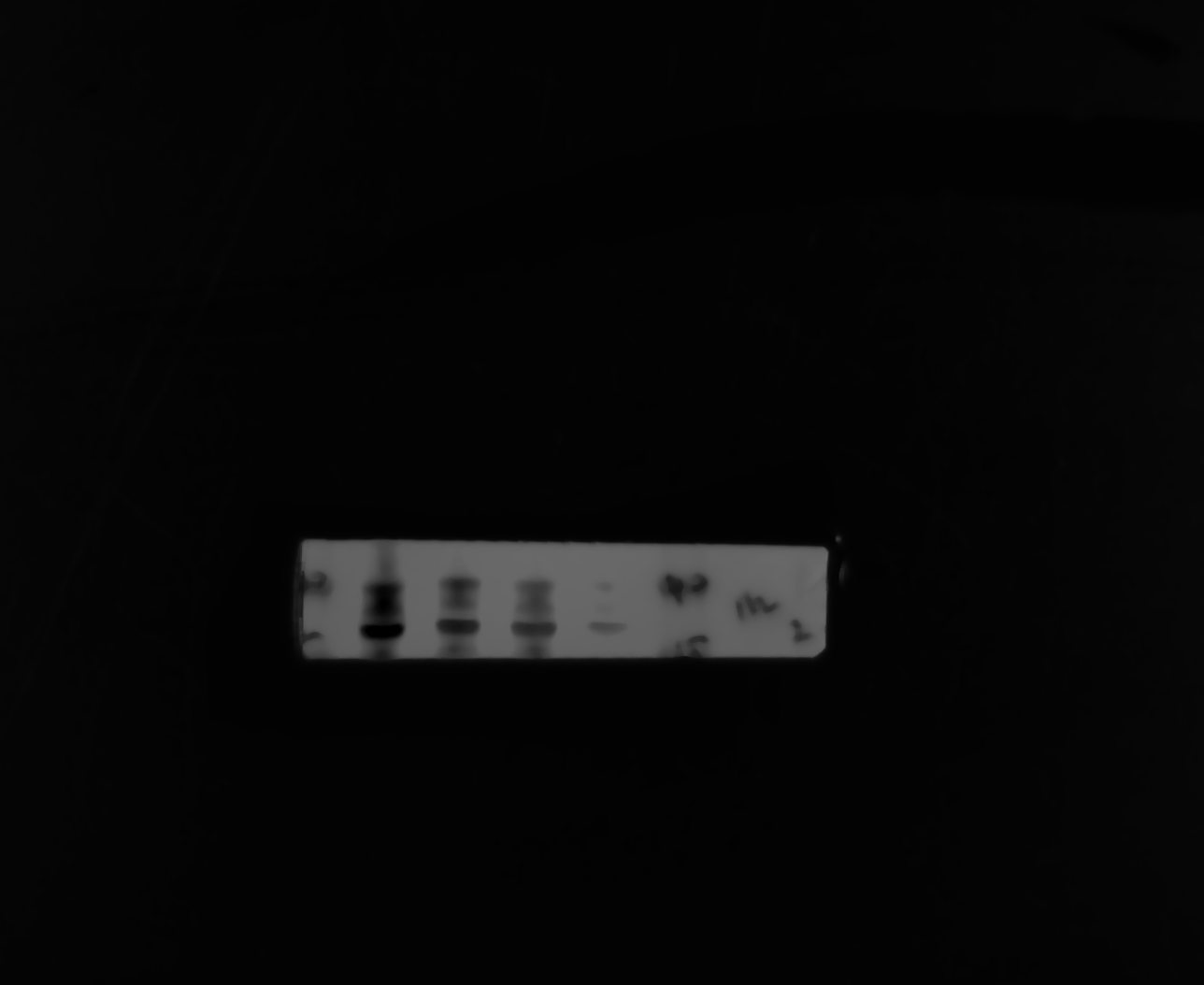

Supplement: Supplementary file 2 — Supplementary Data [file 41420_2026_3089_MOESM2_ESM.zip › Original Data File/Figure7-A Original Data/162-ATP.tif]

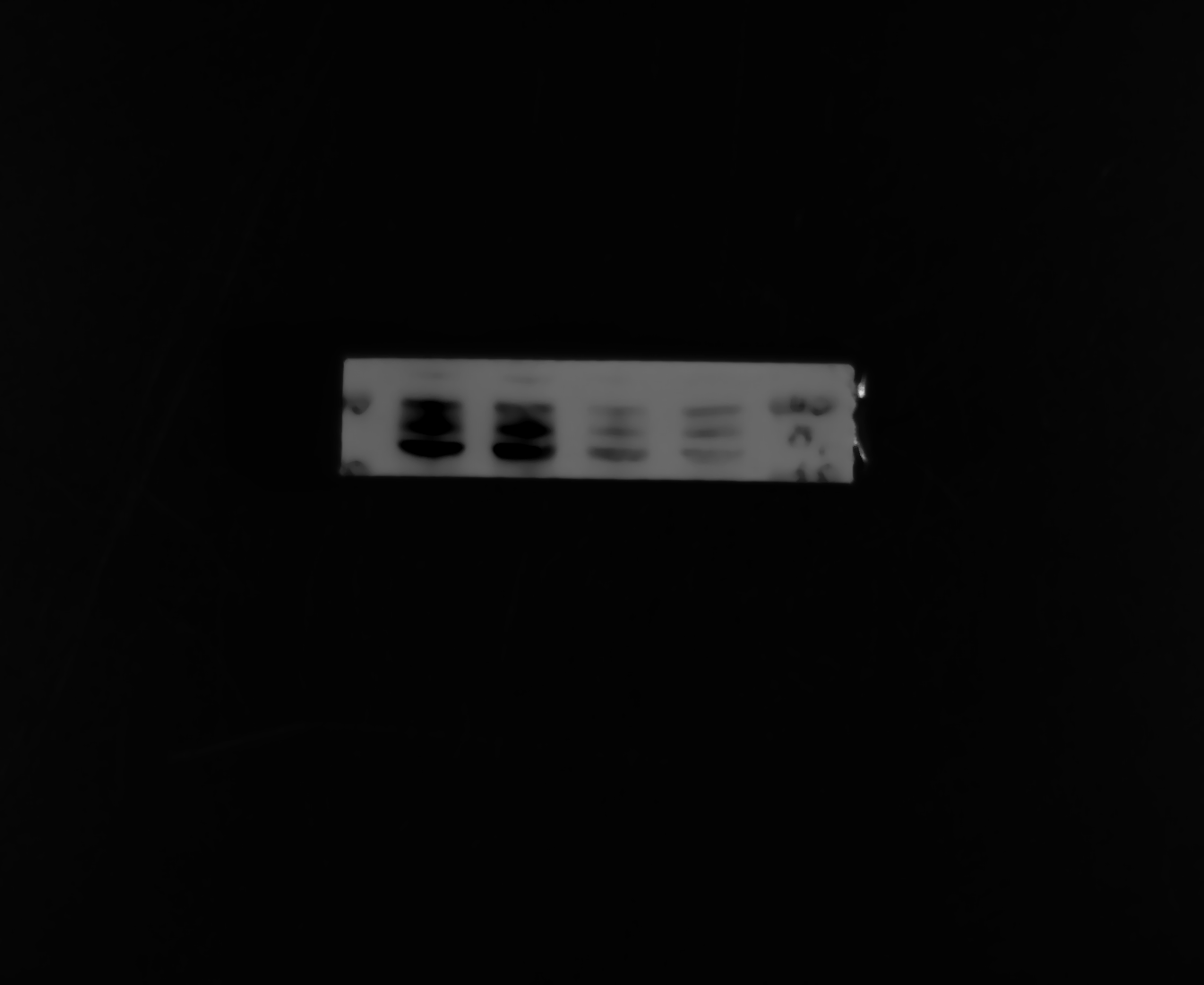

Supplement: Supplementary file 2 — Supplementary Data [file 41420_2026_3089_MOESM2_ESM.zip › Original Data File/Figure7-A Original Data/85-ATP.tif]

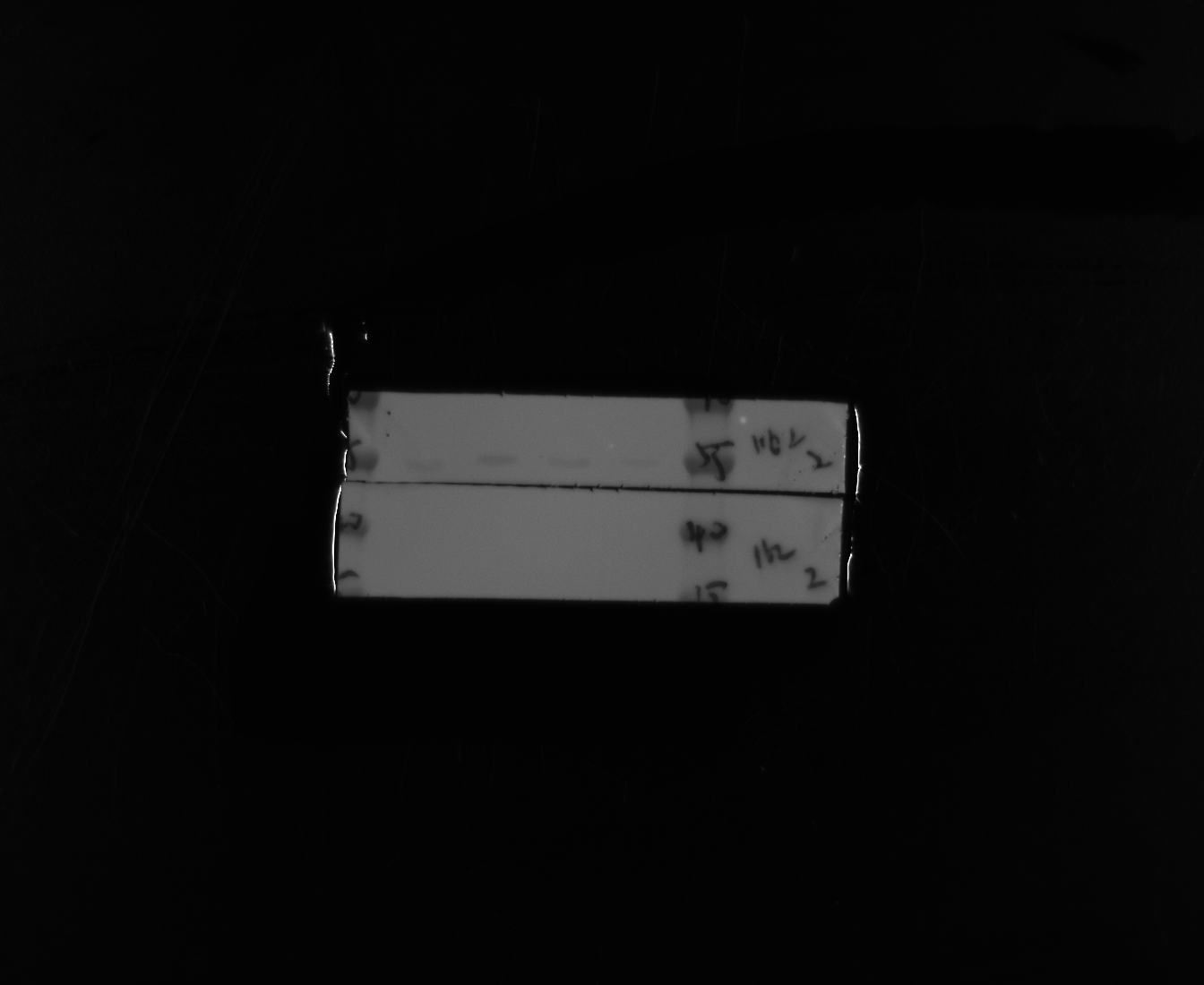

Supplement: Supplementary file 2 — Supplementary Data [file 41420_2026_3089_MOESM2_ESM.zip › Original Data File/Figure7-A Original Data/162-he_marker.tif]

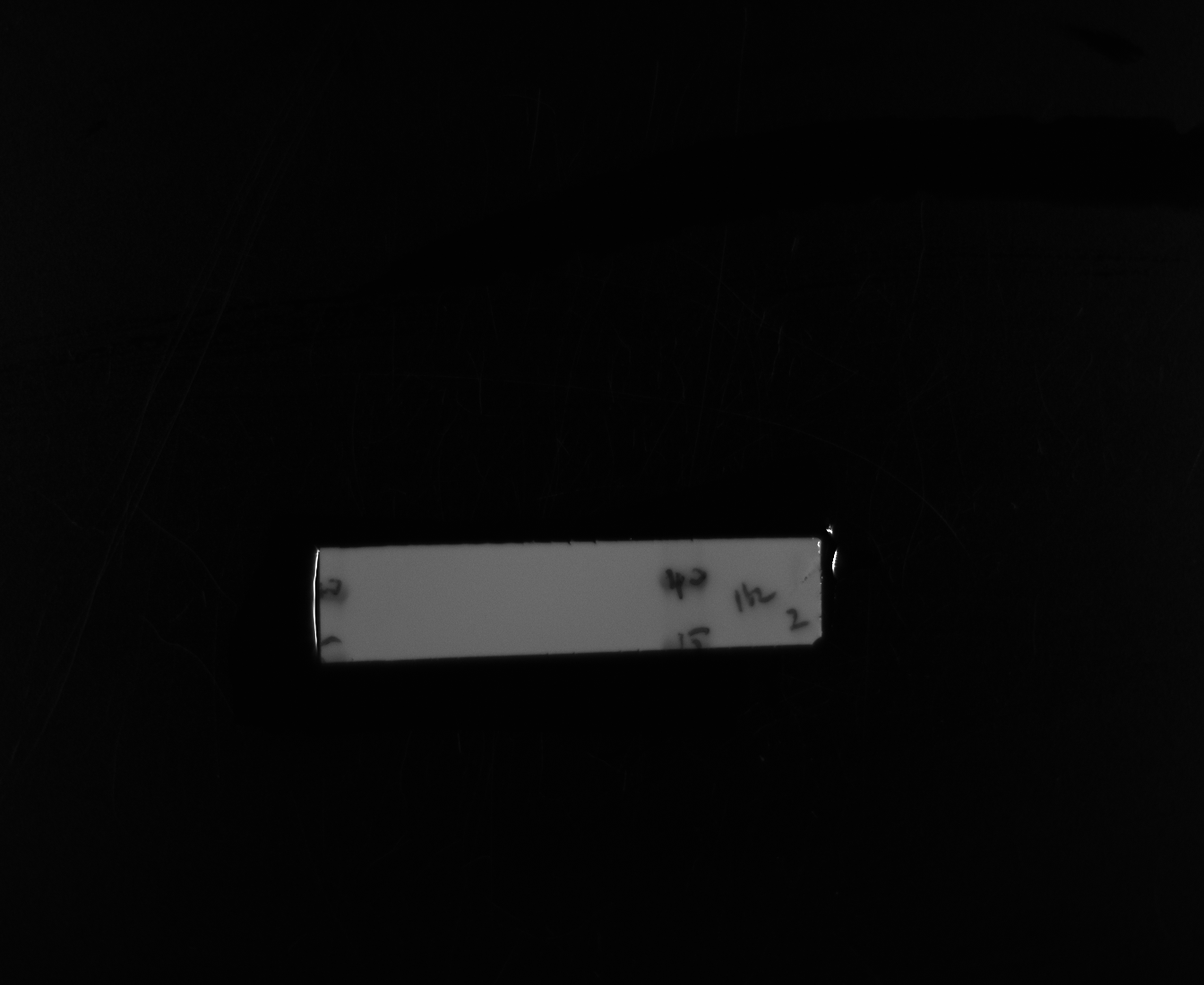

Supplement: Supplementary file 2 — Supplementary Data [file 41420_2026_3089_MOESM2_ESM.zip › Original Data File/Figure7-A Original Data/162-ATP_marker.tif]

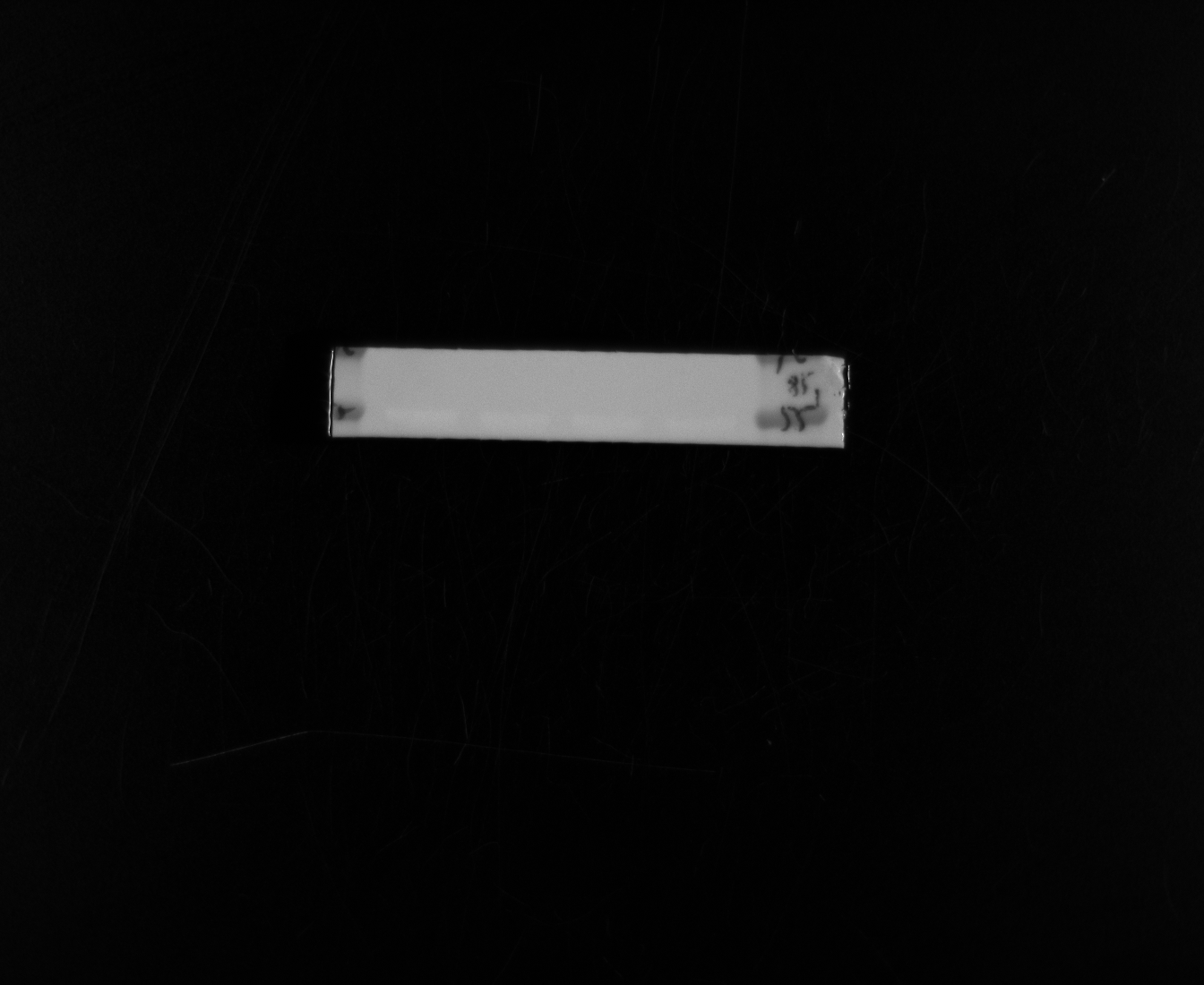

Supplement: Supplementary file 2 — Supplementary Data [file 41420_2026_3089_MOESM2_ESM.zip › Original Data File/Figure7-A Original Data/85-TUB_marker.tif]

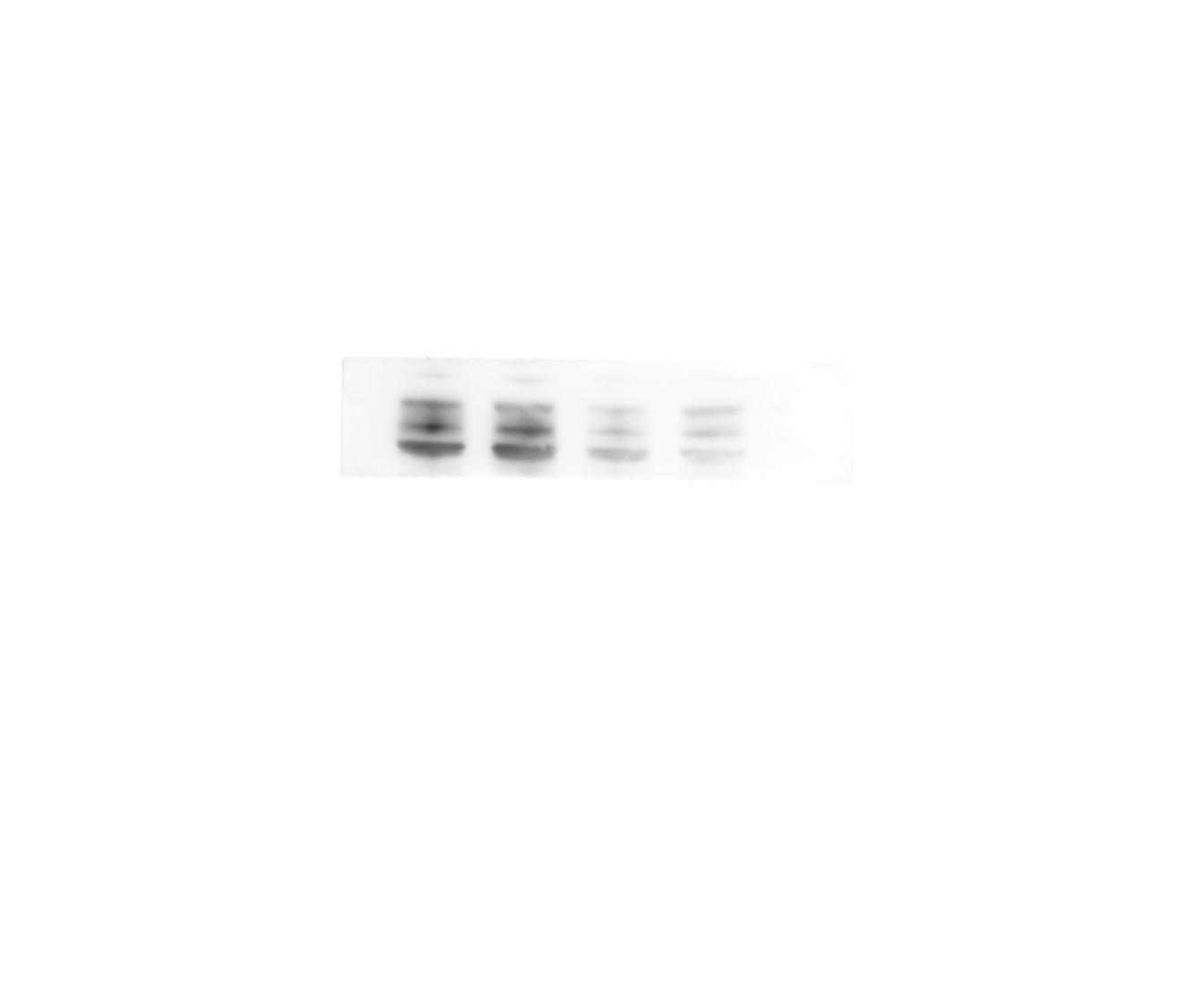

Supplement: Supplementary file 2 — Supplementary Data [file 41420_2026_3089_MOESM2_ESM.zip › Original Data File/Figure7-A Original Data/85-ATP_chemi.tif]

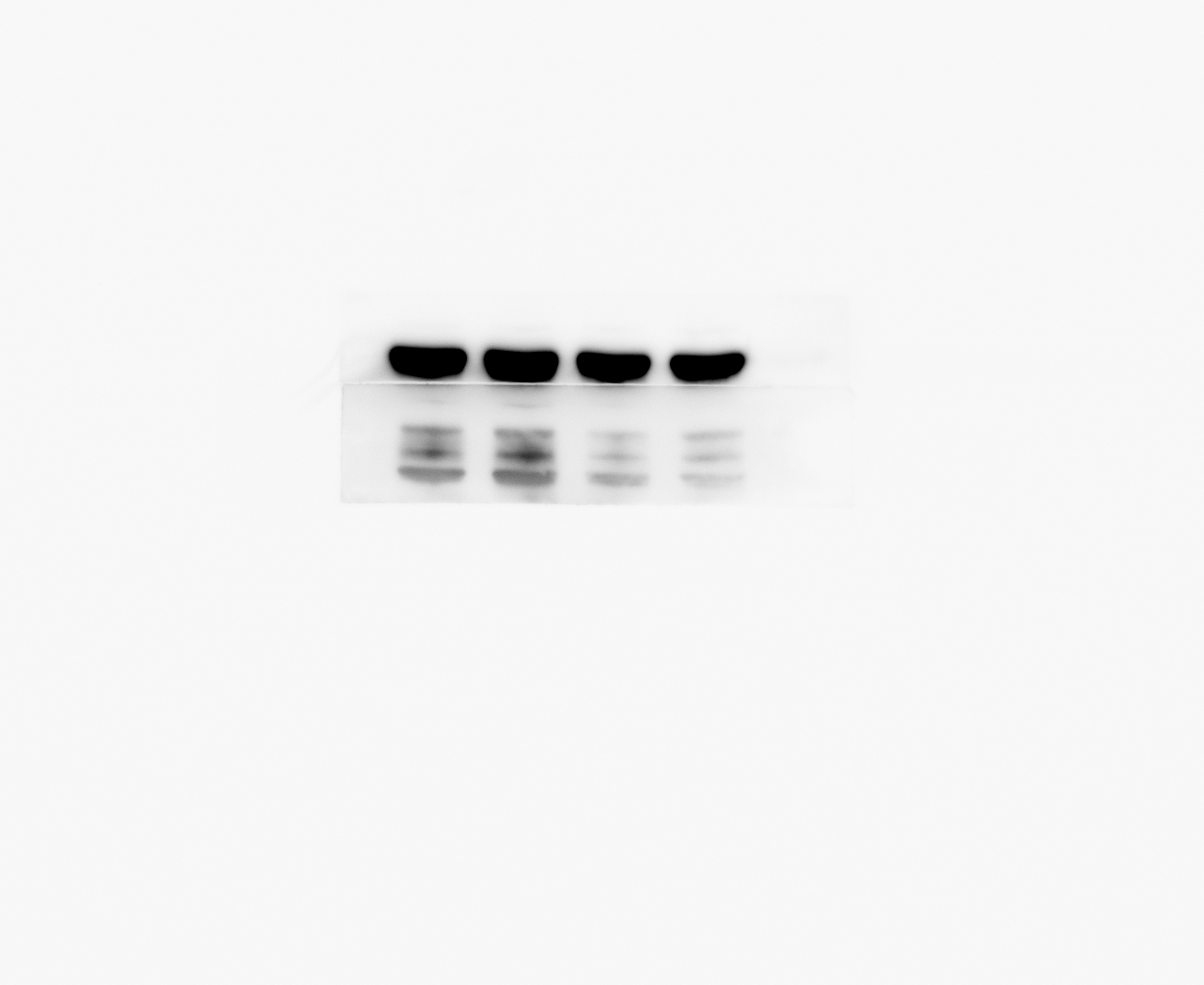

Supplement: Supplementary file 2 — Supplementary Data [file 41420_2026_3089_MOESM2_ESM.zip › Original Data File/Figure7-A Original Data/85-HE_chemi.tif]

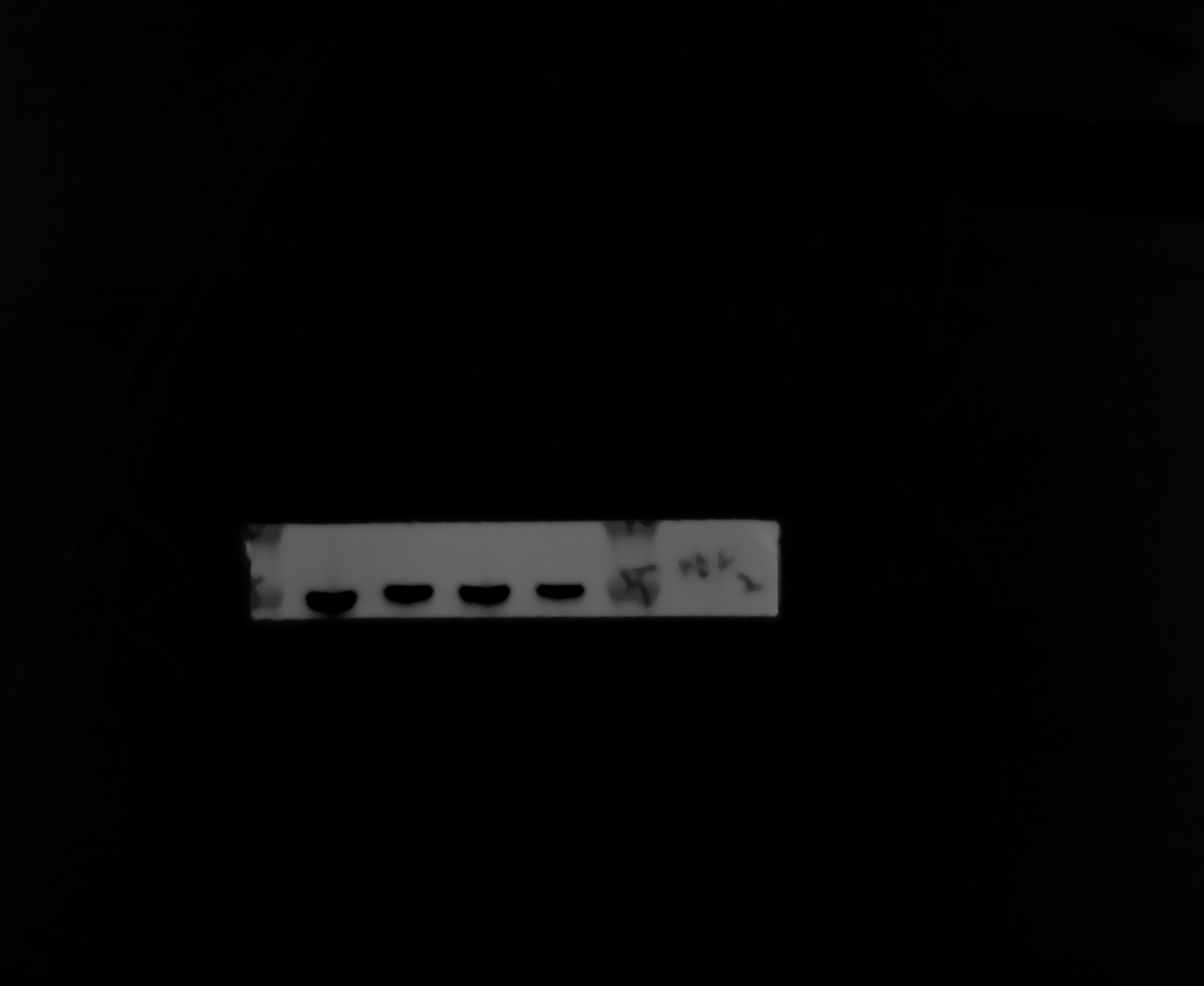

Supplement: Supplementary file 2 — Supplementary Data [file 41420_2026_3089_MOESM2_ESM.zip › Original Data File/Figure7-A Original Data/162-2-TUB.tif]

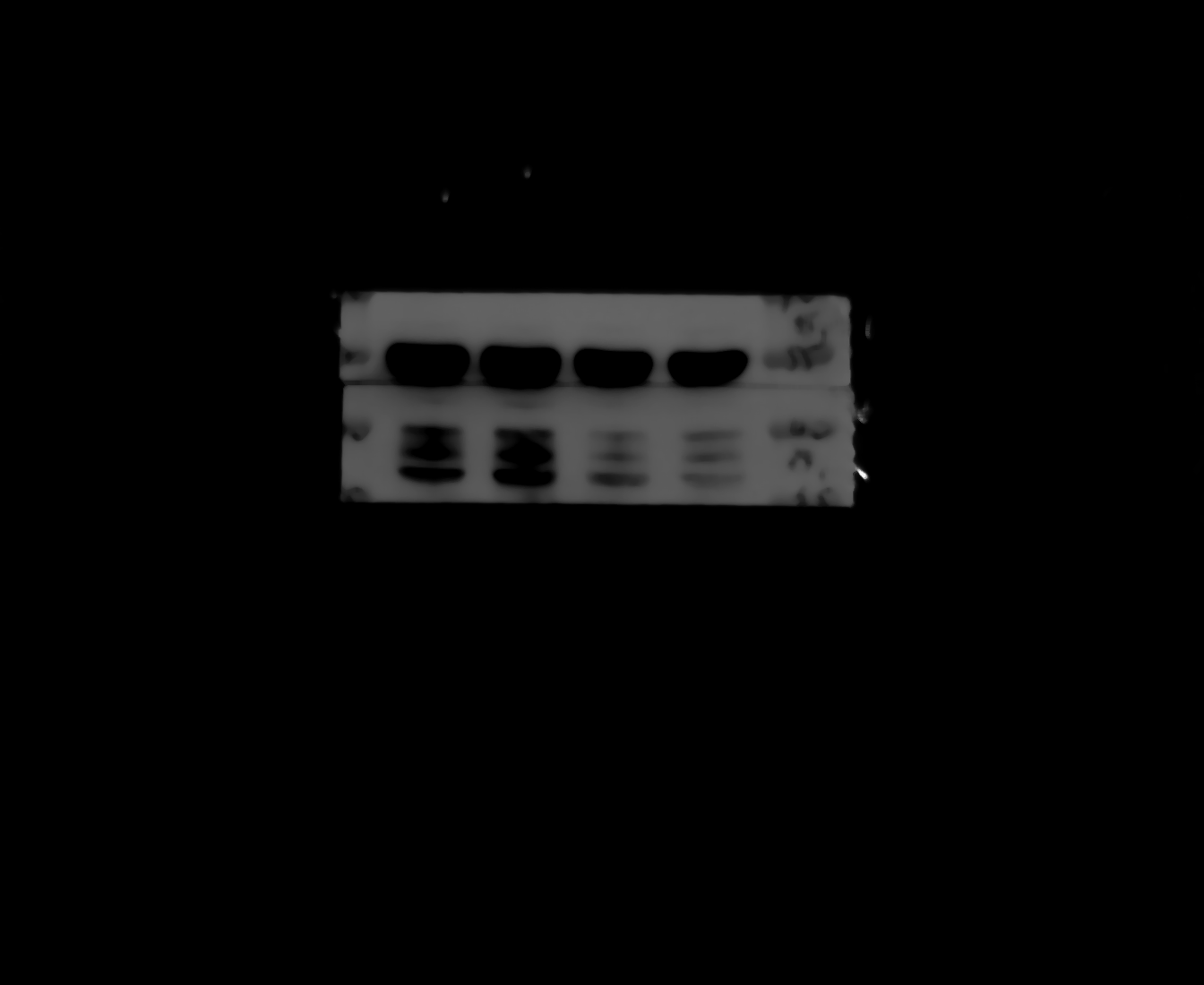

Supplement: Supplementary file 2 — Supplementary Data [file 41420_2026_3089_MOESM2_ESM.zip › Original Data File/Figure7-A Original Data/85-HE.tif]

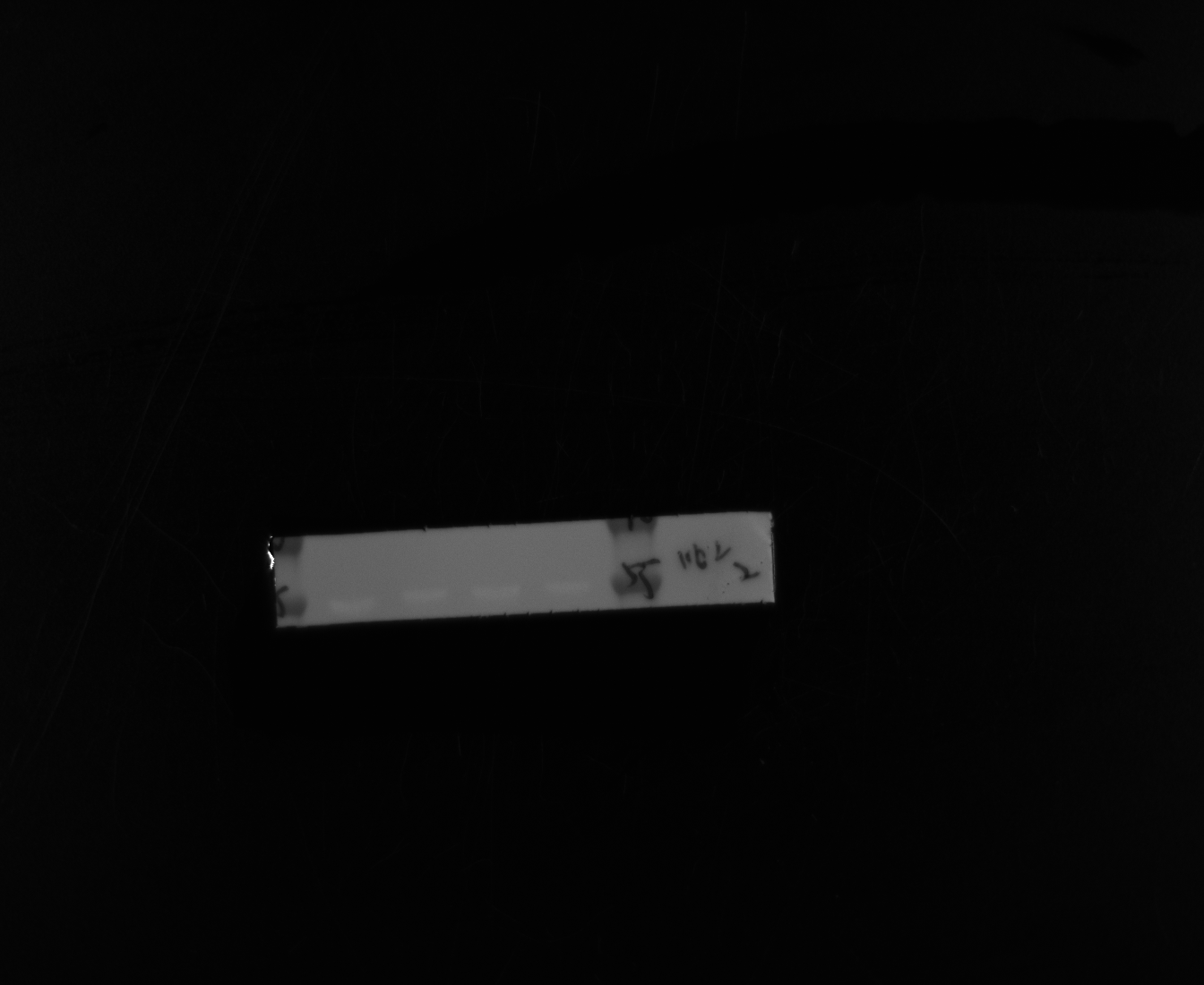

Supplement: Supplementary file 2 — Supplementary Data [file 41420_2026_3089_MOESM2_ESM.zip › Original Data File/Figure7-A Original Data/162-2-TUB_marker.tif]

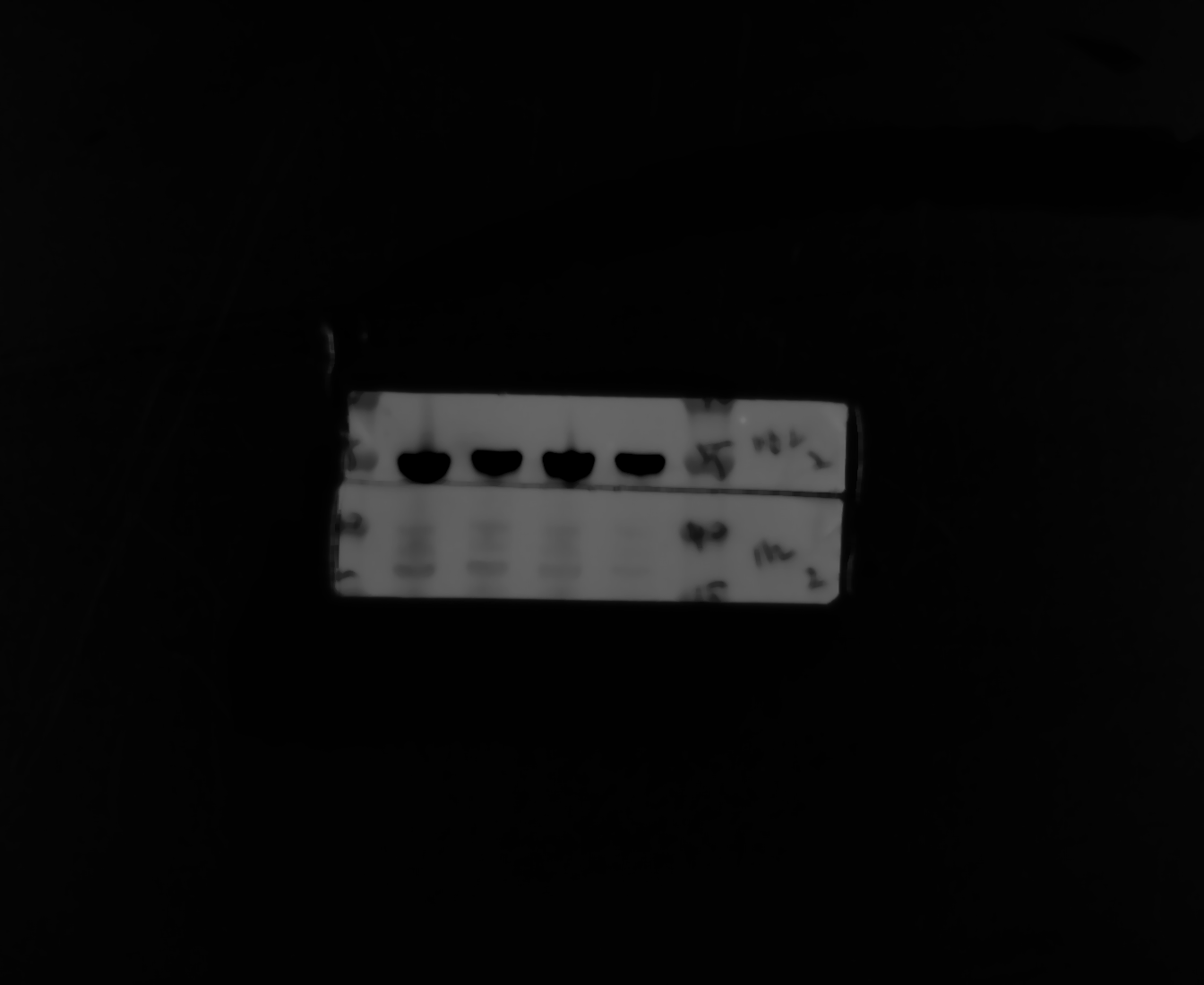

Supplement: Supplementary file 2 — Supplementary Data [file 41420_2026_3089_MOESM2_ESM.zip › Original Data File/Figure7-A Original Data/162-he.tif]

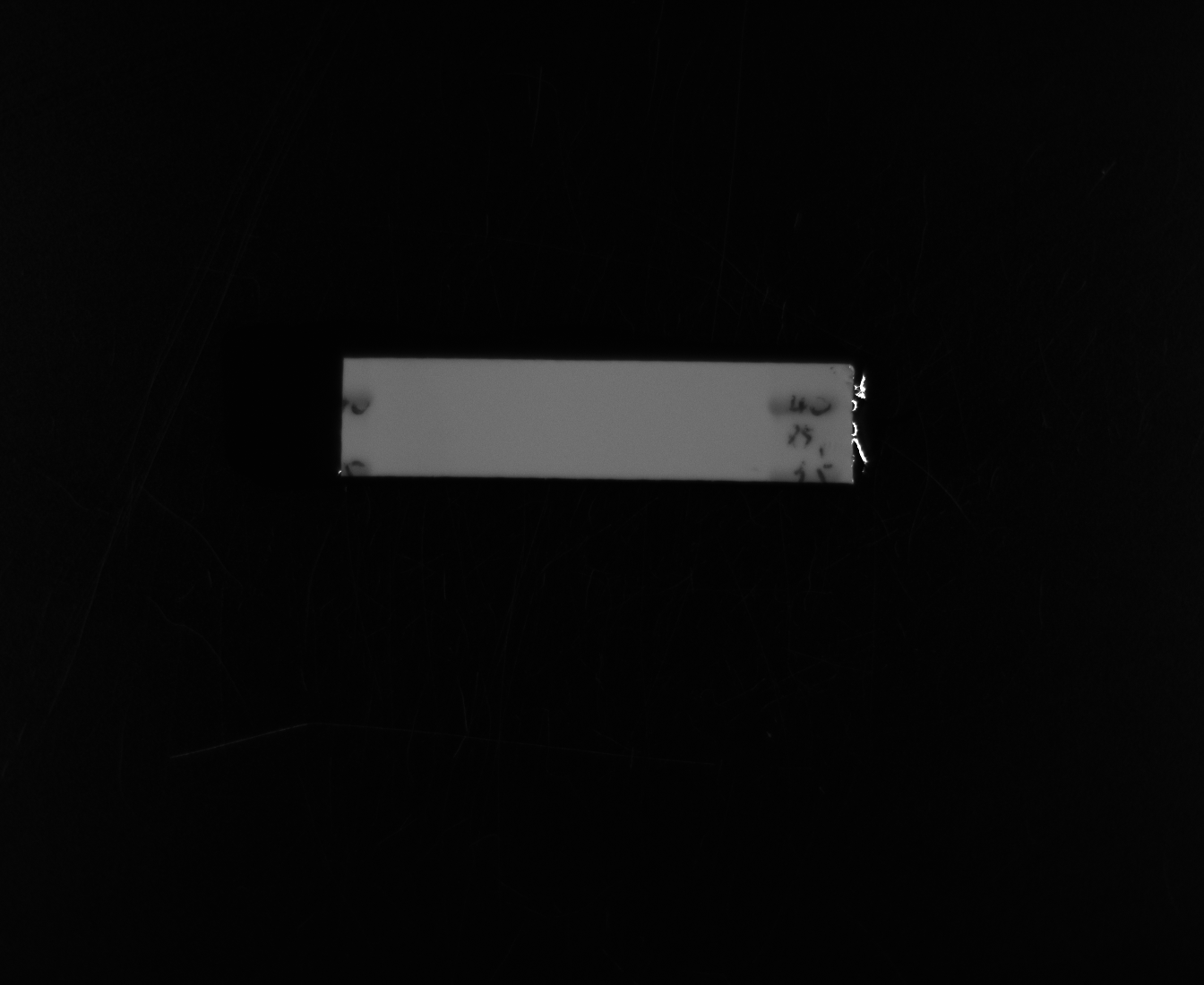

Supplement: Supplementary file 2 — Supplementary Data [file 41420_2026_3089_MOESM2_ESM.zip › Original Data File/Figure7-A Original Data/85-ATP_marker.tif]

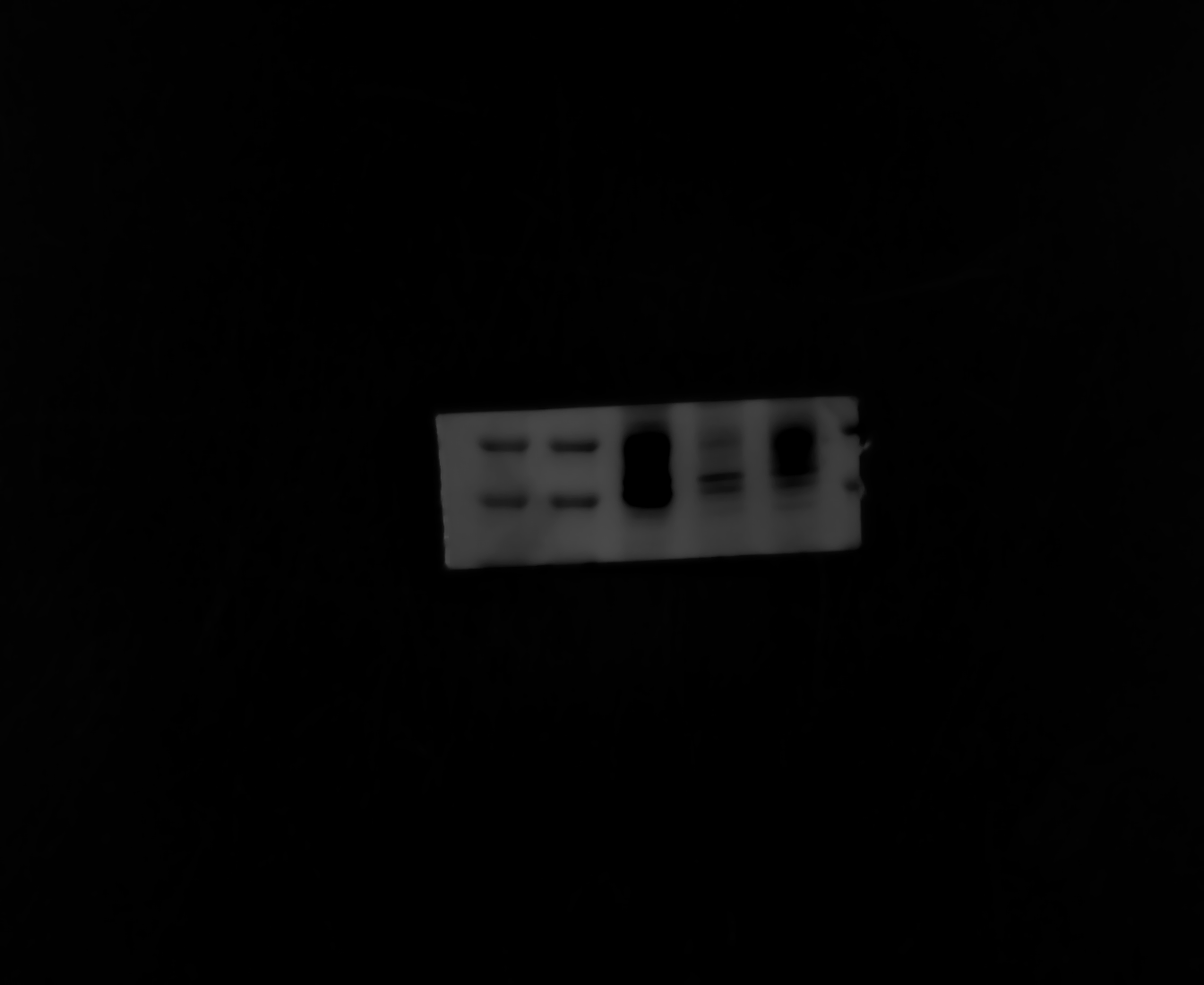

Supplement: Supplementary file 2 — Supplementary Data [file 41420_2026_3089_MOESM2_ESM.zip › Original Data File/Figure8-B Original Data/Fig8B 85-oe.tif]

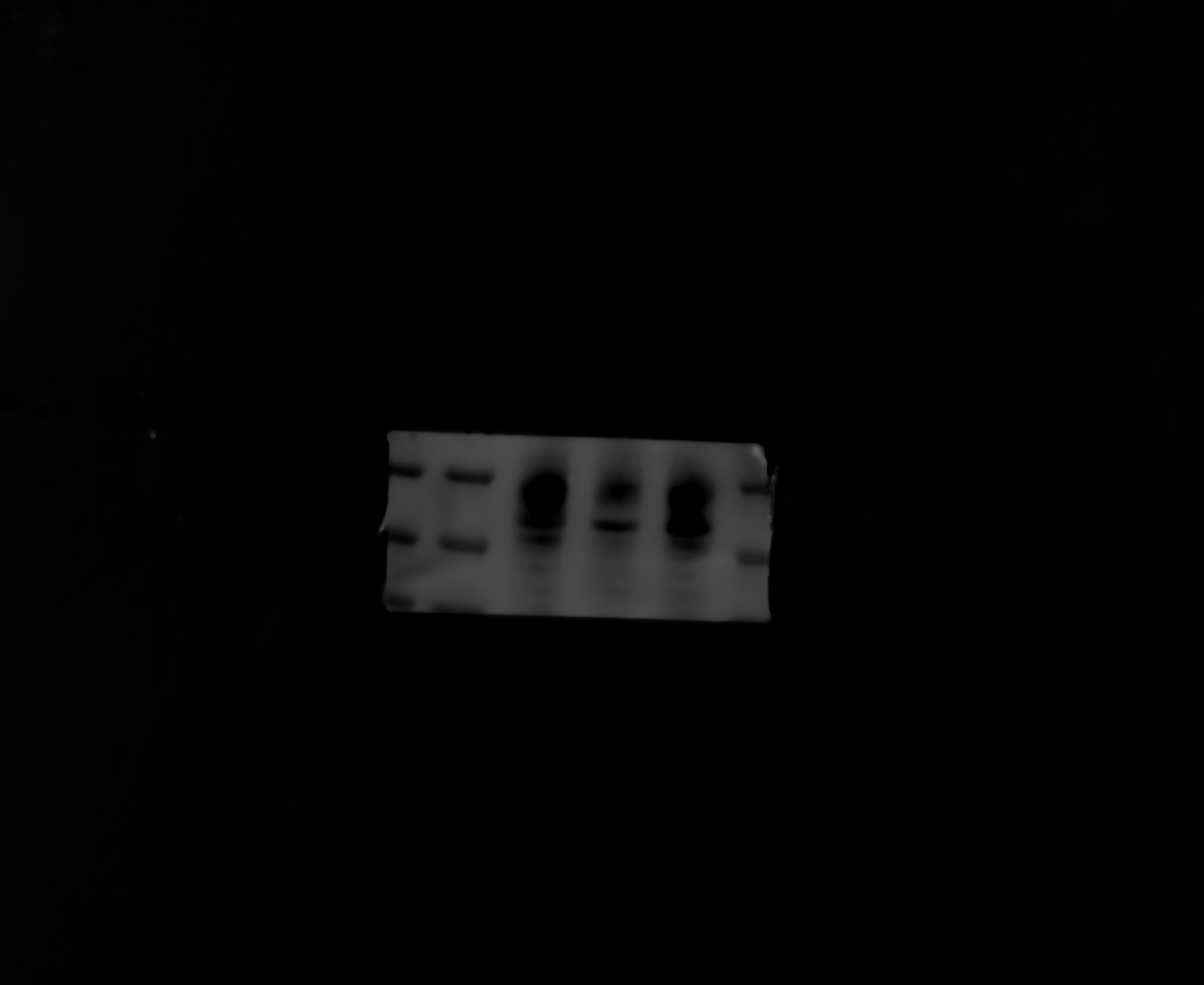

Supplement: Supplementary file 2 — Supplementary Data [file 41420_2026_3089_MOESM2_ESM.zip › Original Data File/Figure8-B Original Data/Fig8BCAL62-OE.tif]

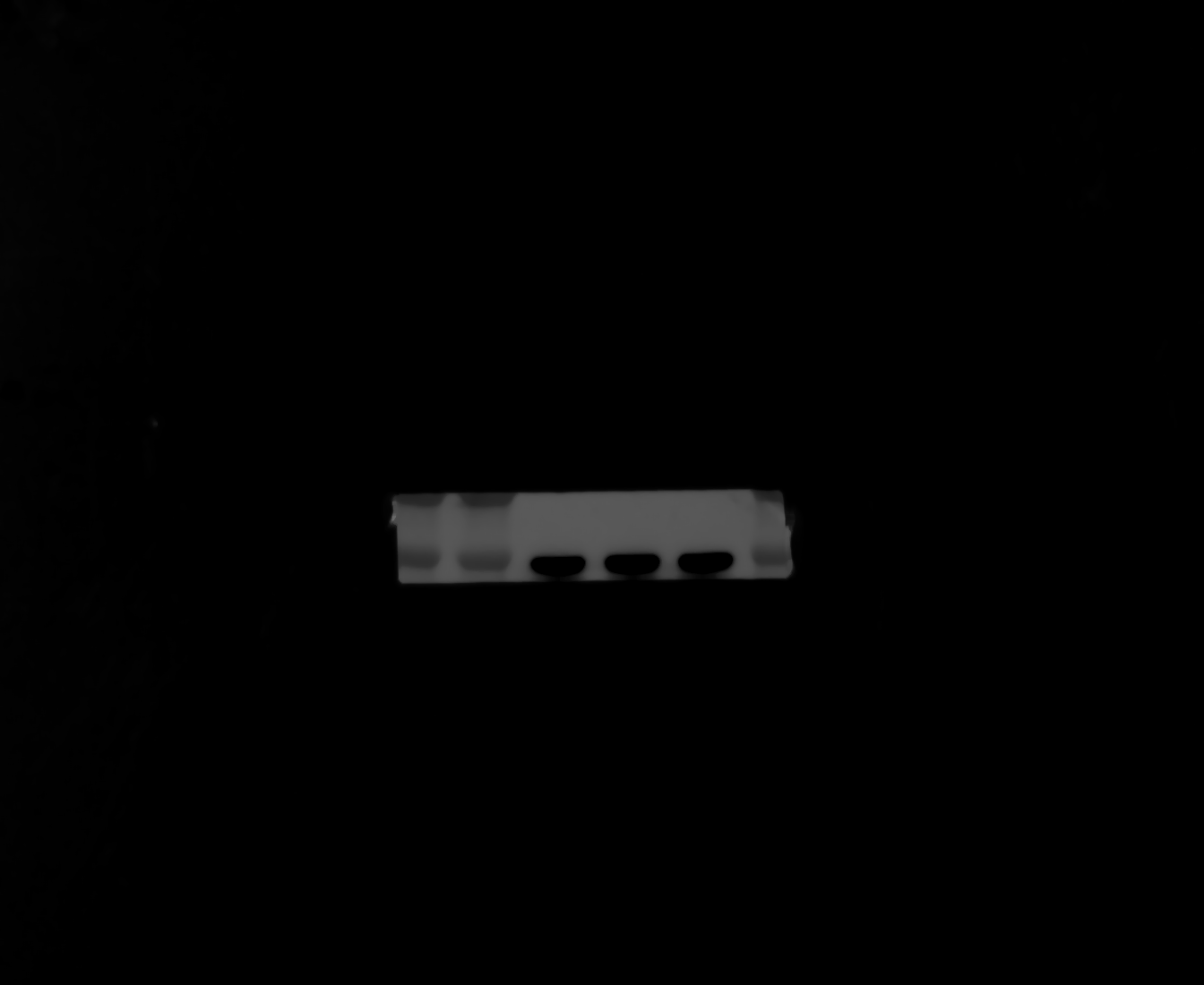

Supplement: Supplementary file 2 — Supplementary Data [file 41420_2026_3089_MOESM2_ESM.zip › Original Data File/Figure8-B Original Data/Fig 8B CAL62-tub.tif]

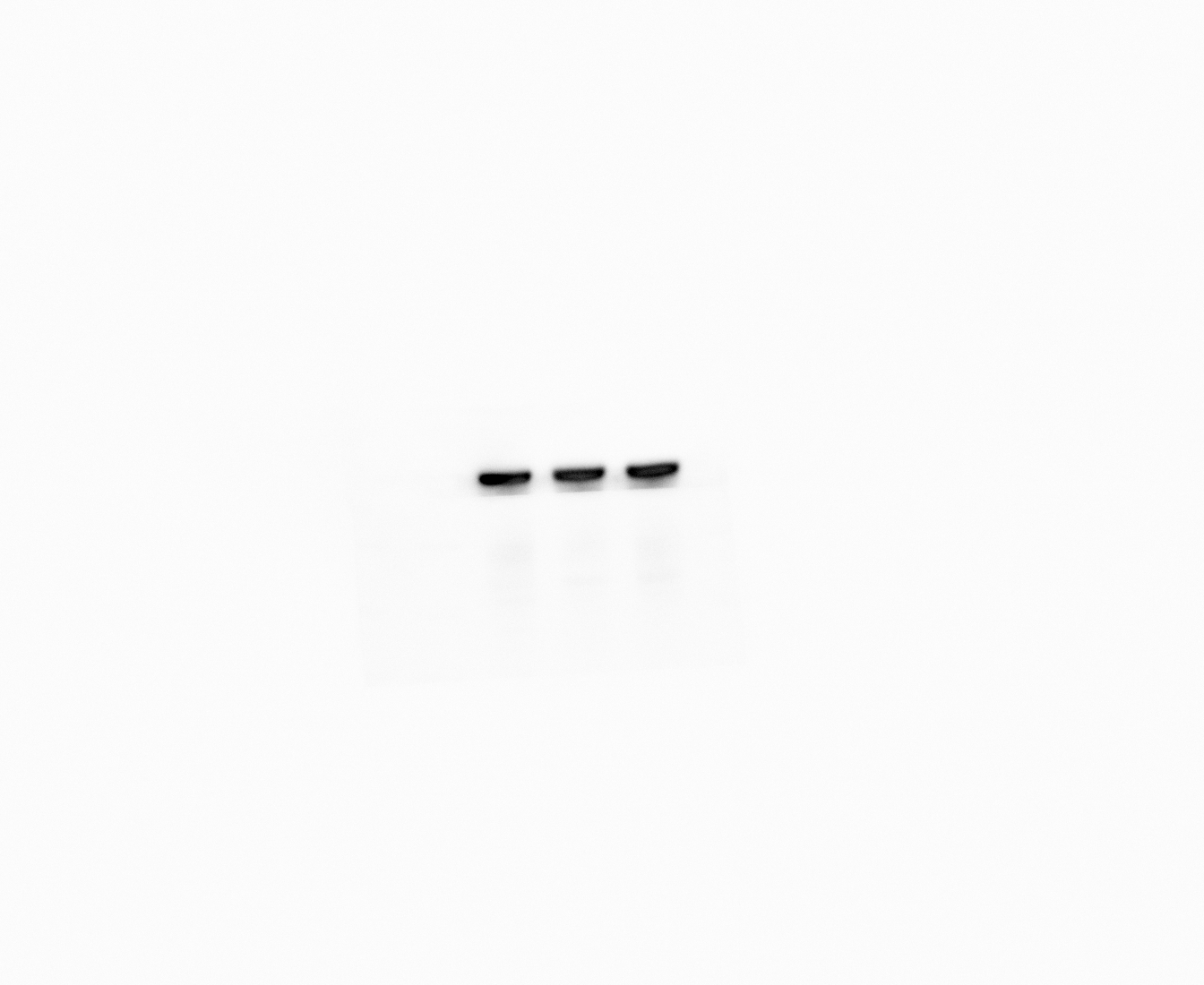

Supplement: Supplementary file 2 — Supplementary Data [file 41420_2026_3089_MOESM2_ESM.zip › Original Data File/Figure8-B Original Data/CAL62-ALL_chemi.tif]

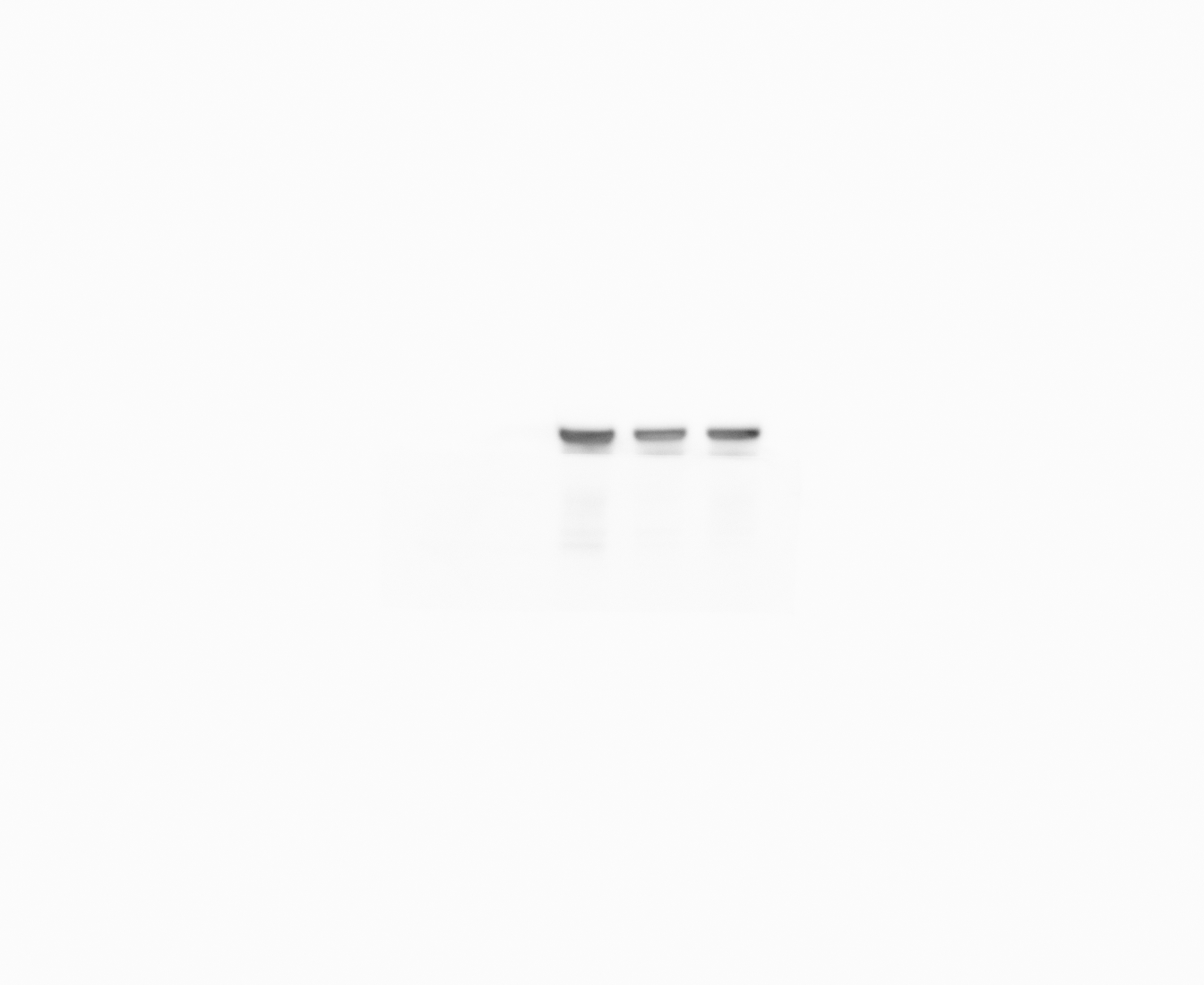

Supplement: Supplementary file 2 — Supplementary Data [file 41420_2026_3089_MOESM2_ESM.zip › Original Data File/Figure8-B Original Data/85-all-2_chemi.tif]

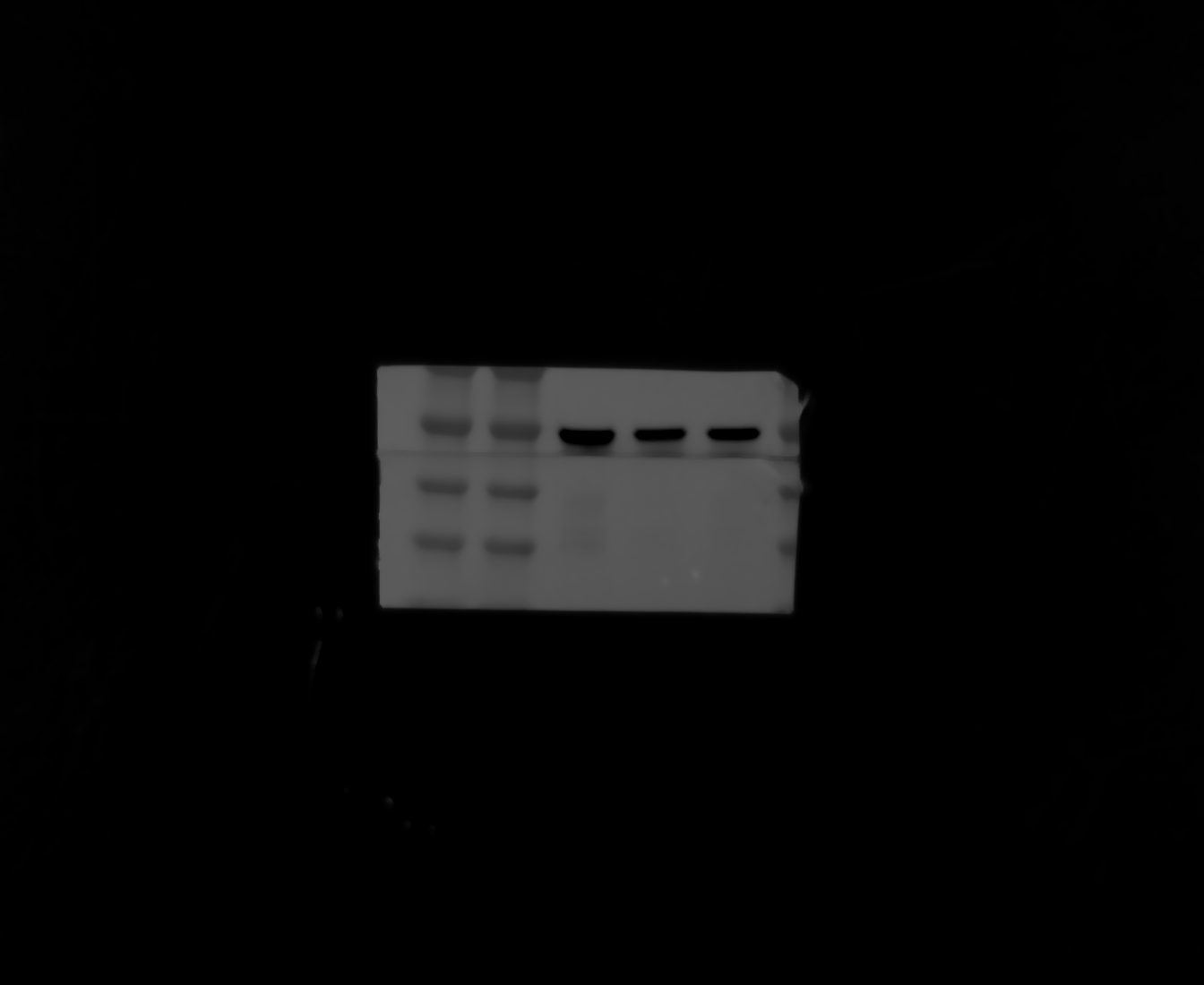

Supplement: Supplementary file 2 — Supplementary Data [file 41420_2026_3089_MOESM2_ESM.zip › Original Data File/Figure8-B Original Data/Fig8B 85-all.tif]

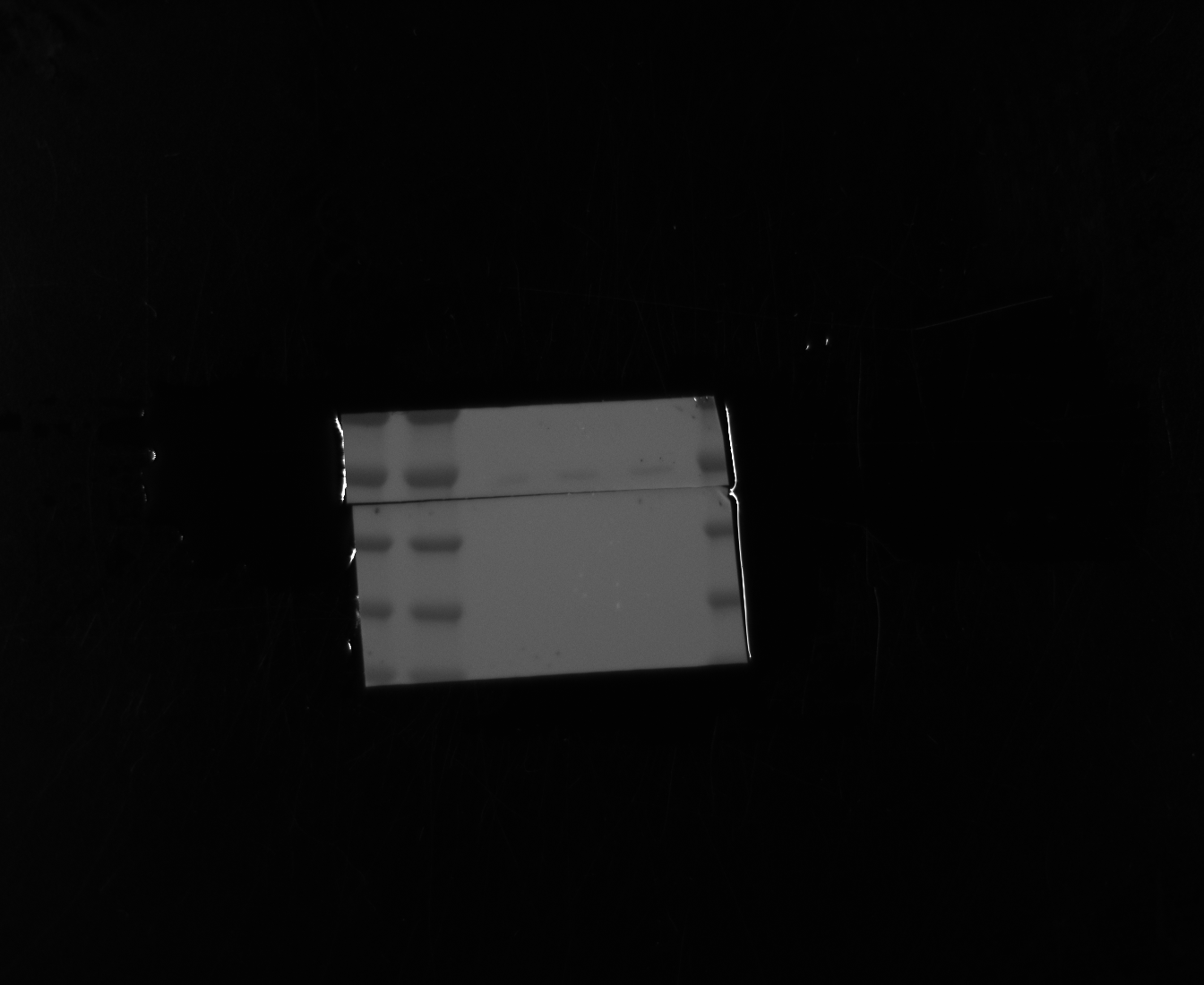

Supplement: Supplementary file 2 — Supplementary Data [file 41420_2026_3089_MOESM2_ESM.zip › Original Data File/Figure8-B Original Data/CAL62-ALL_marker.tif]

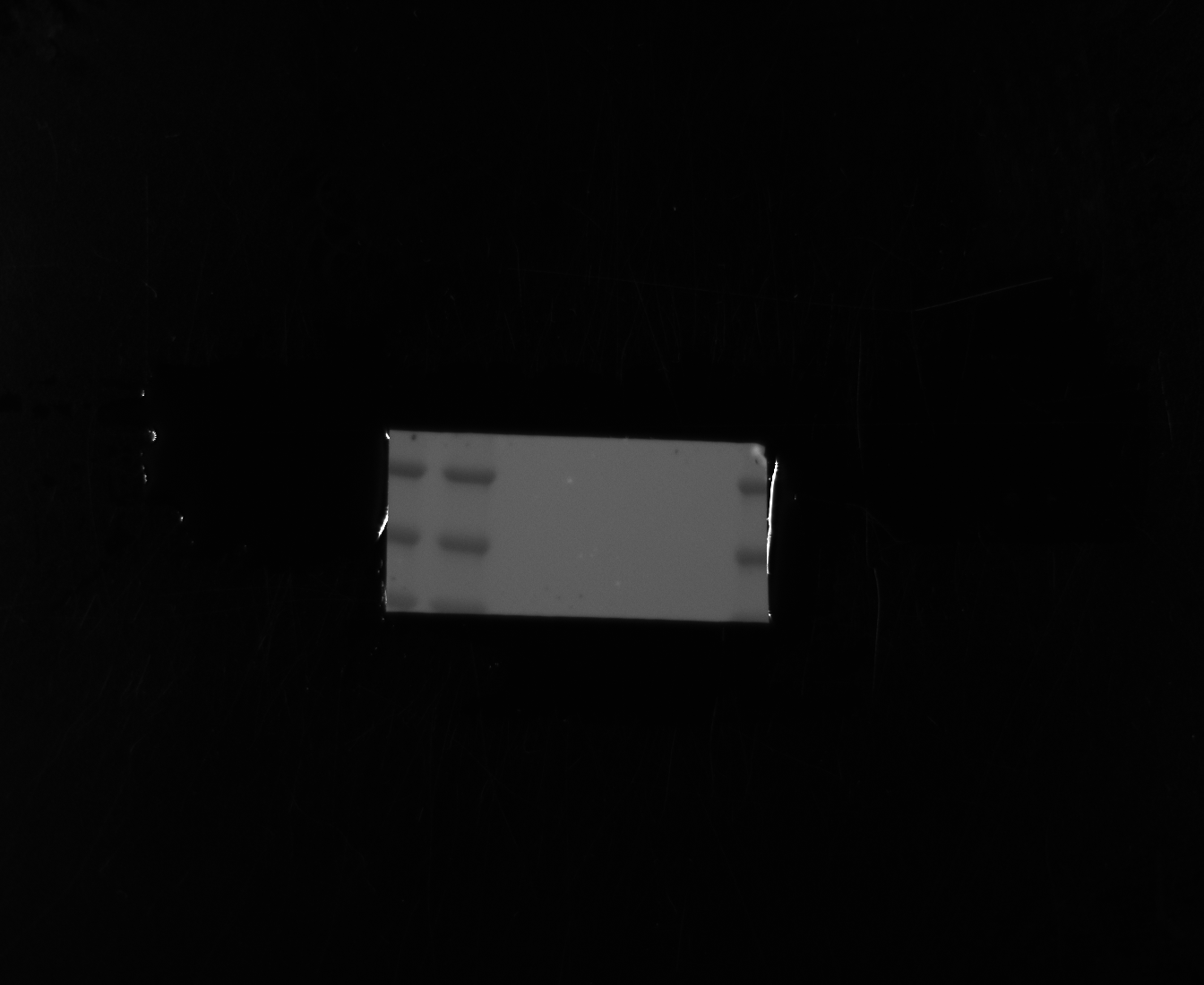

Supplement: Supplementary file 2 — Supplementary Data [file 41420_2026_3089_MOESM2_ESM.zip › Original Data File/Figure8-B Original Data/CAL62-OE_marker.tif]

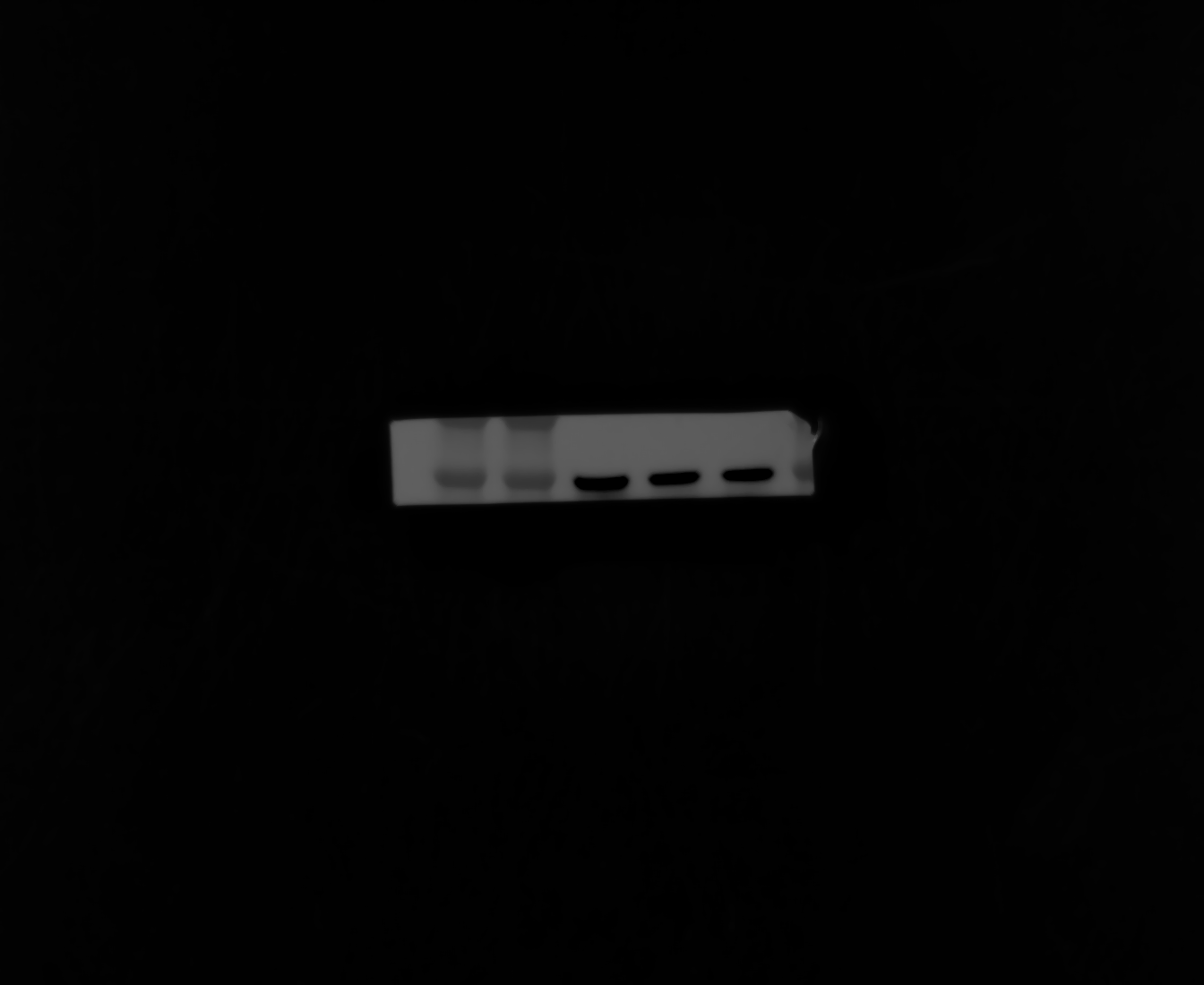

Supplement: Supplementary file 2 — Supplementary Data [file 41420_2026_3089_MOESM2_ESM.zip › Original Data File/Figure8-B Original Data/Fig8B 85-tub.tif]

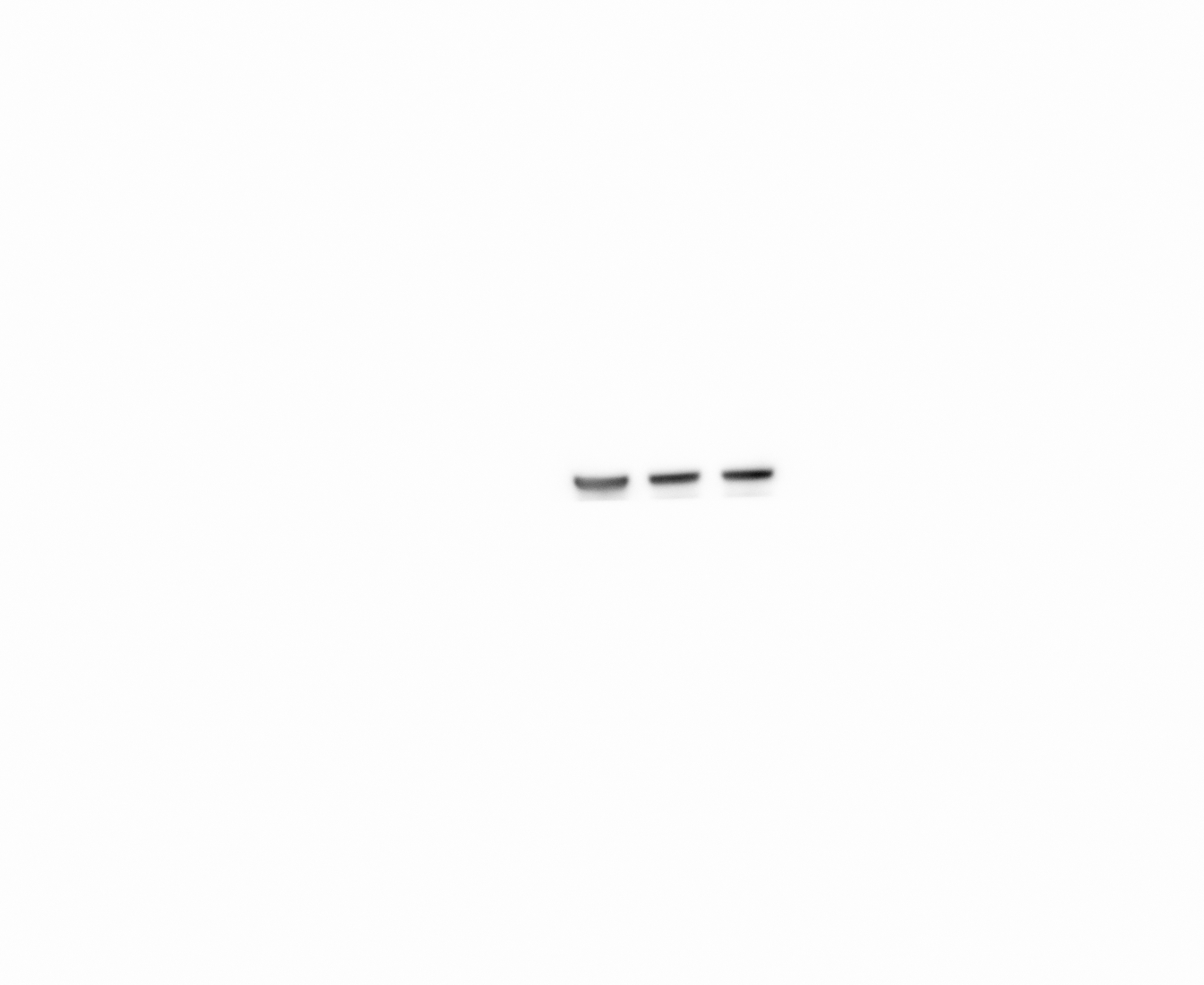

Supplement: Supplementary file 2 — Supplementary Data [file 41420_2026_3089_MOESM2_ESM.zip › Original Data File/Figure8-B Original Data/85-tub-oe_chemi.tif]

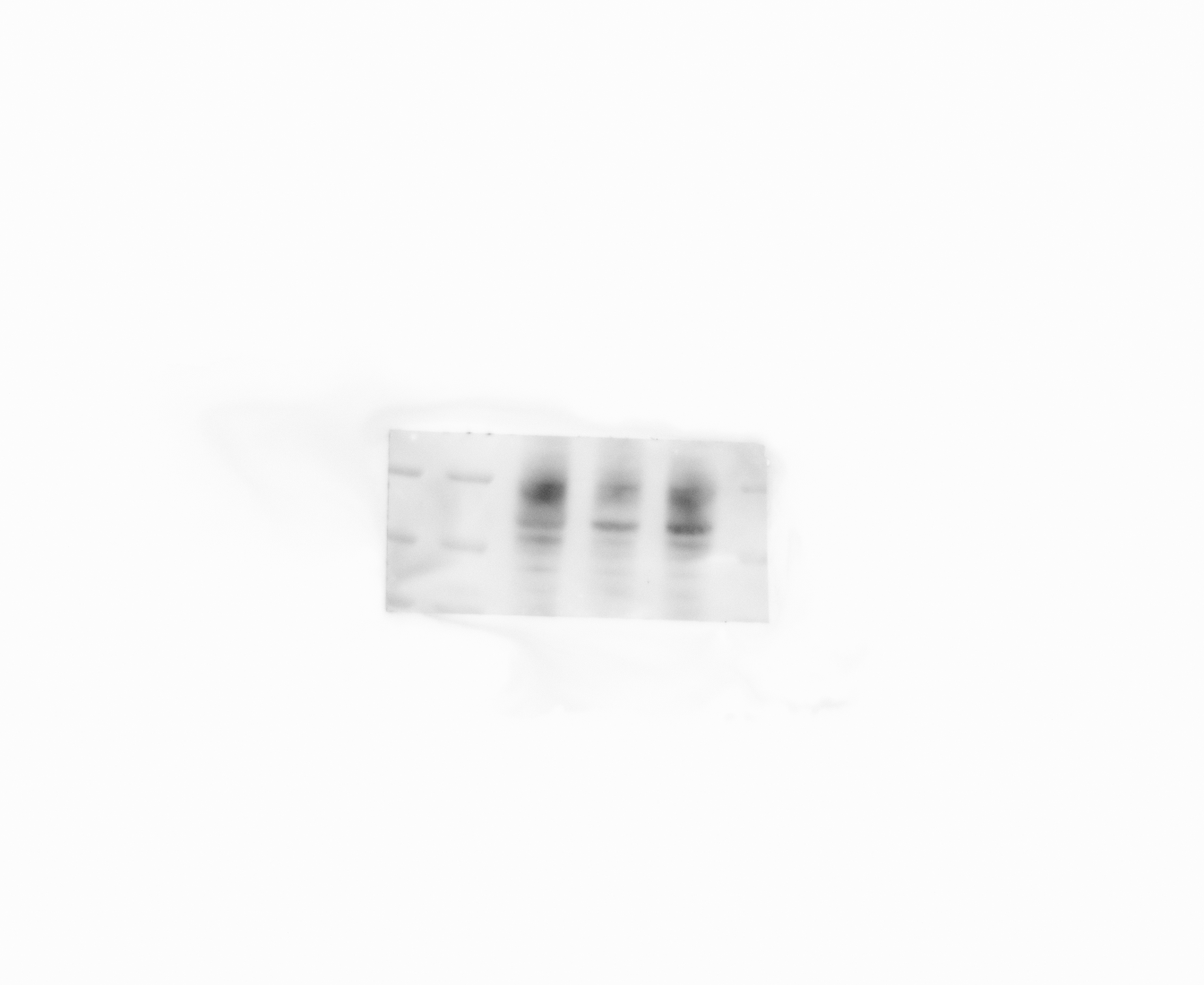

Supplement: Supplementary file 2 — Supplementary Data [file 41420_2026_3089_MOESM2_ESM.zip › Original Data File/Figure8-B Original Data/CAL62-OE_chemi.tif]

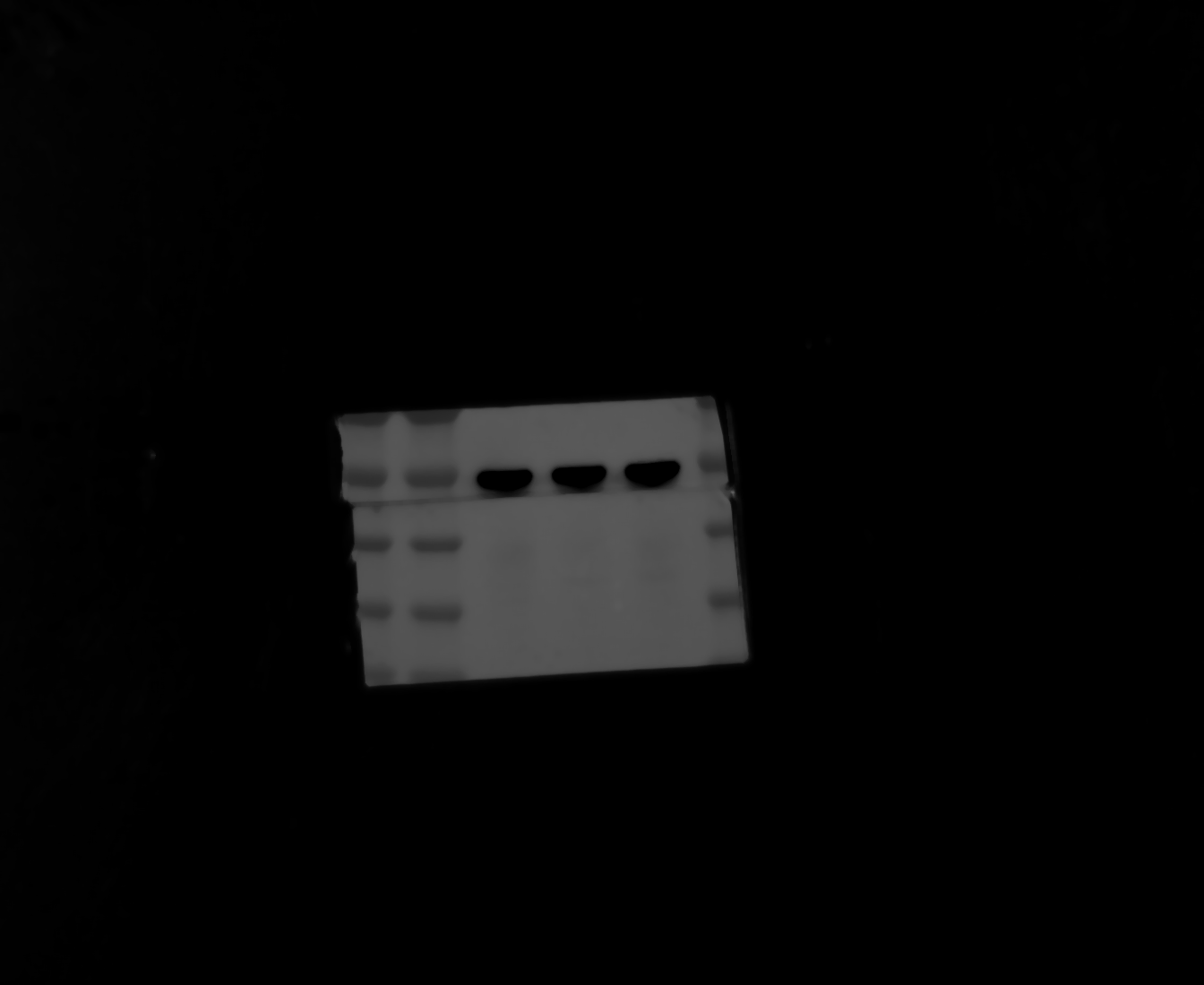

Supplement: Supplementary file 2 — Supplementary Data [file 41420_2026_3089_MOESM2_ESM.zip › Original Data File/Figure8-B Original Data/Fig 8BCAL62 ALL .tif]

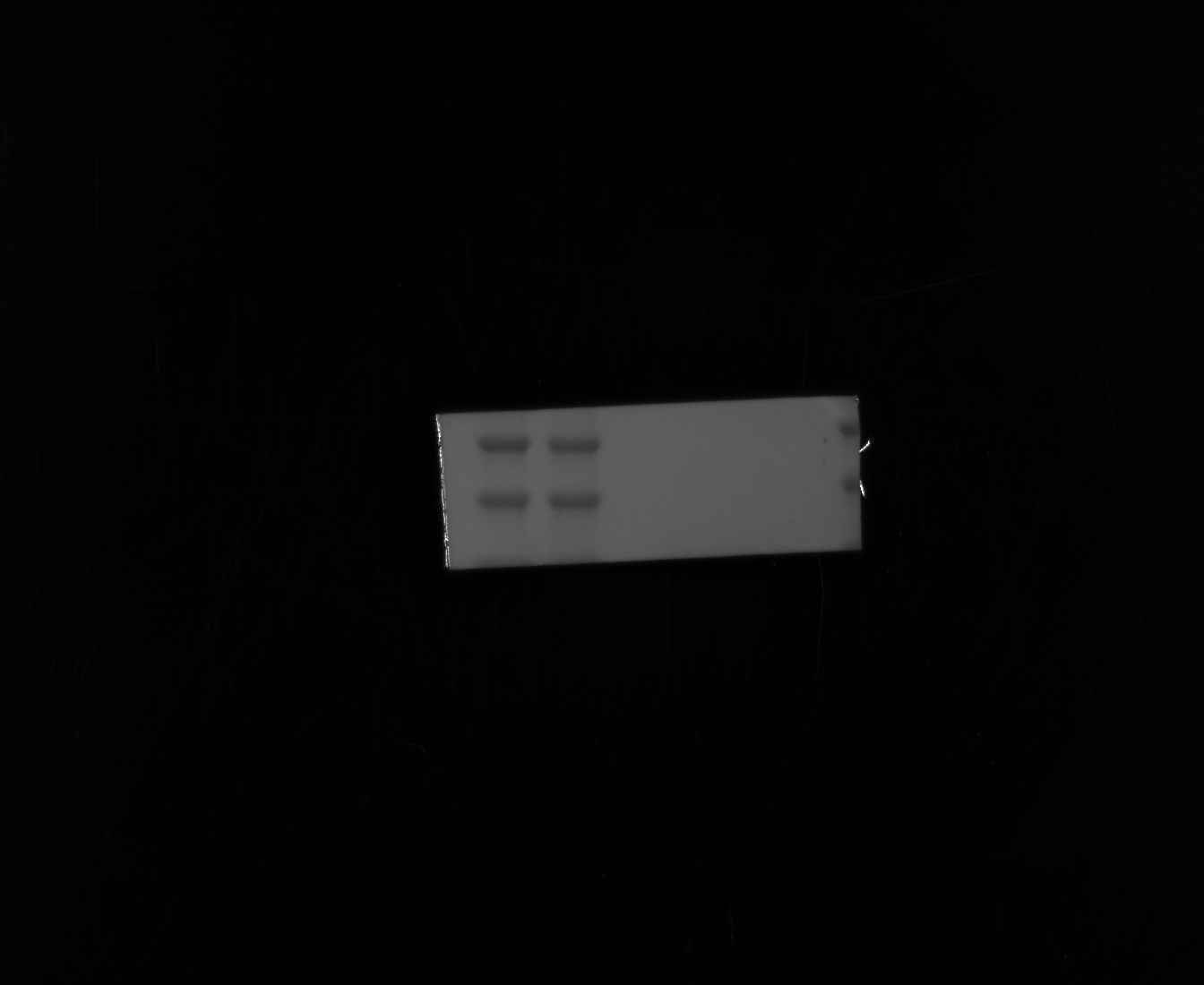

Supplement: Supplementary file 2 — Supplementary Data [file 41420_2026_3089_MOESM2_ESM.zip › Original Data File/Figure8-B Original Data/85-oe_marker.tif]

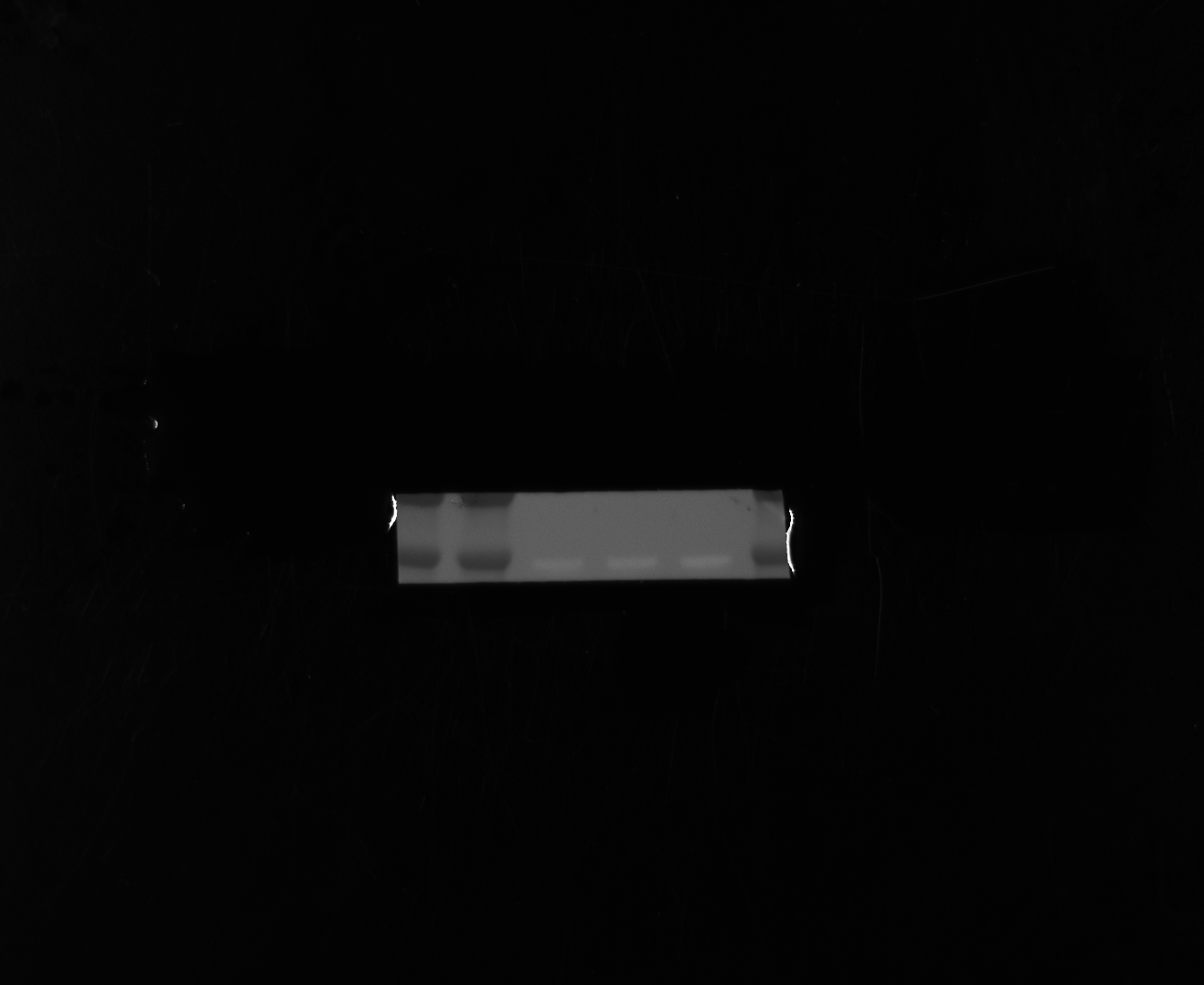

Supplement: Supplementary file 2 — Supplementary Data [file 41420_2026_3089_MOESM2_ESM.zip › Original Data File/Figure8-B Original Data/CAL62-TUB_marker.tif]

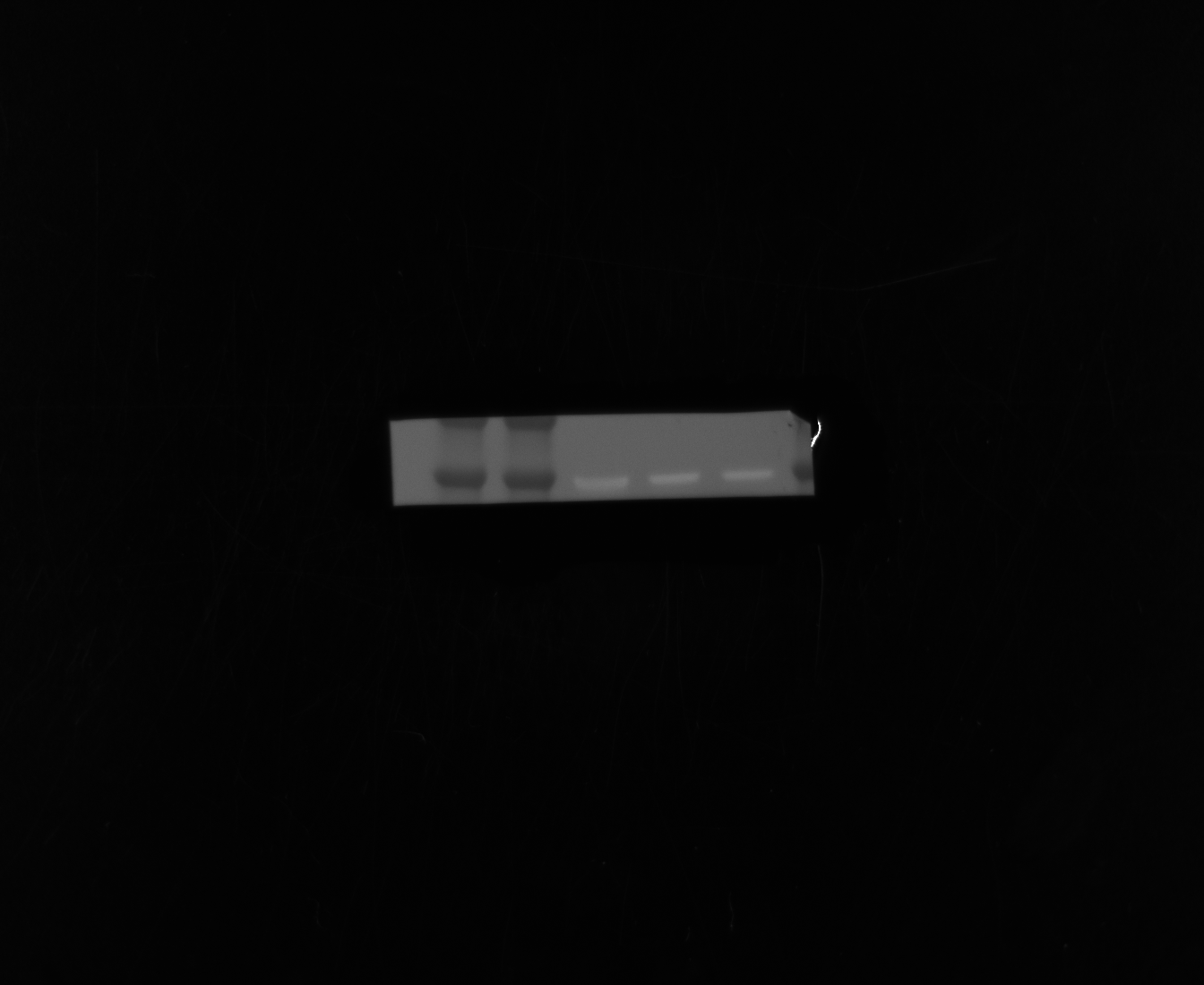

Supplement: Supplementary file 2 — Supplementary Data [file 41420_2026_3089_MOESM2_ESM.zip › Original Data File/Figure8-B Original Data/85-tub-oe_marker.tif]

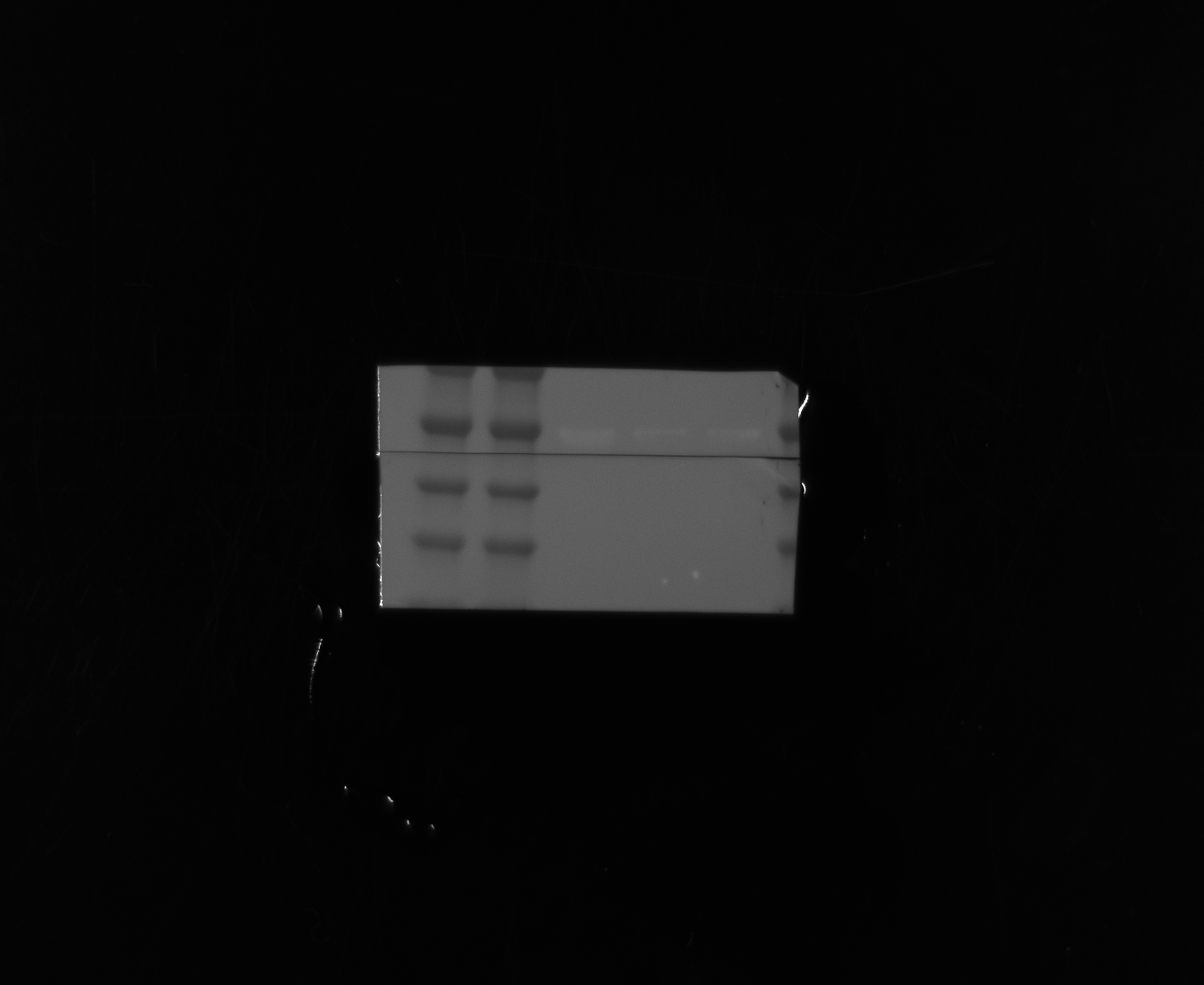

Supplement: Supplementary file 2 — Supplementary Data [file 41420_2026_3089_MOESM2_ESM.zip › Original Data File/Figure8-B Original Data/85-all-2_marker.tif]

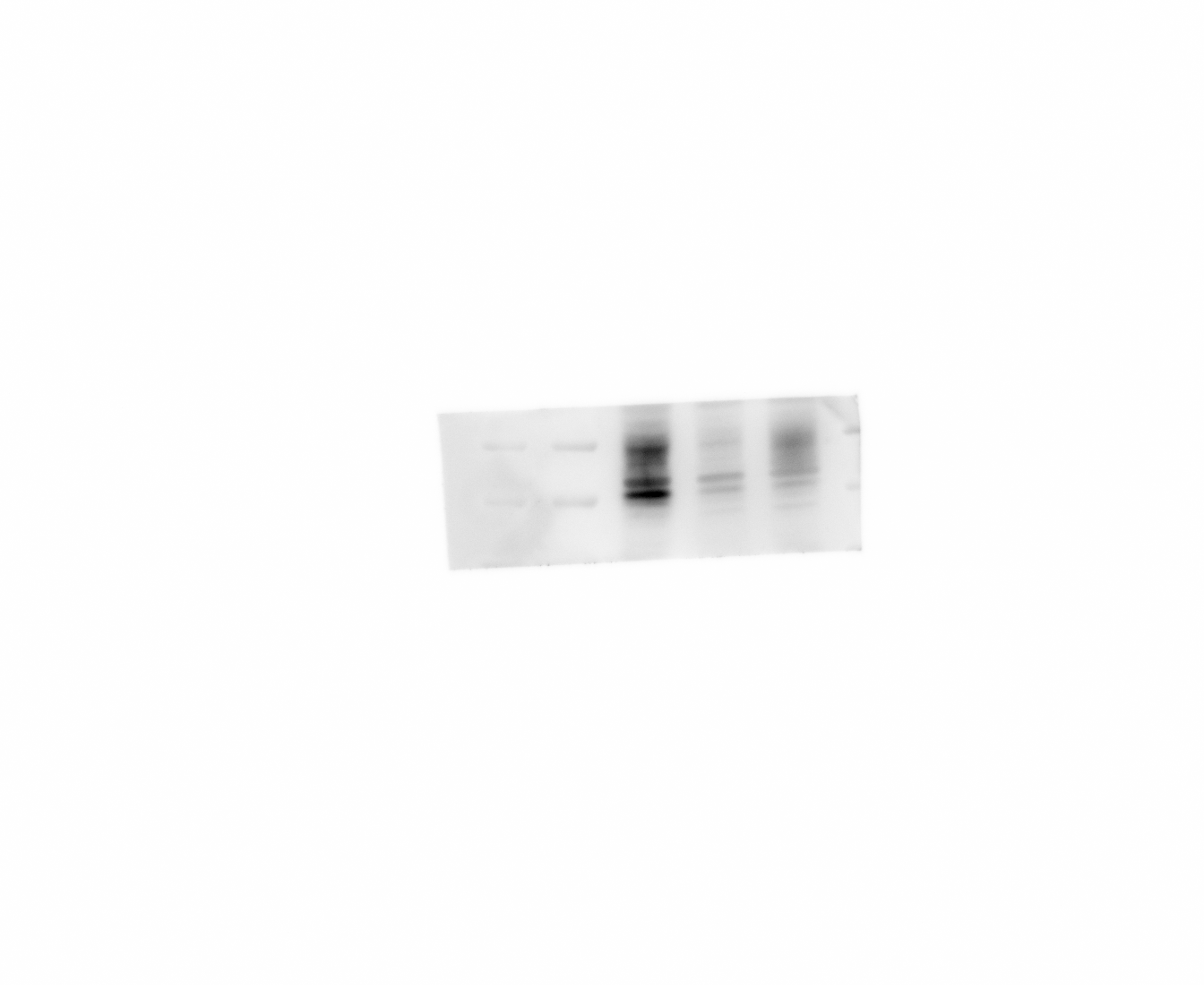

Supplement: Supplementary file 2 — Supplementary Data [file 41420_2026_3089_MOESM2_ESM.zip › Original Data File/Figure8-B Original Data/85-oe_chemi.tif]

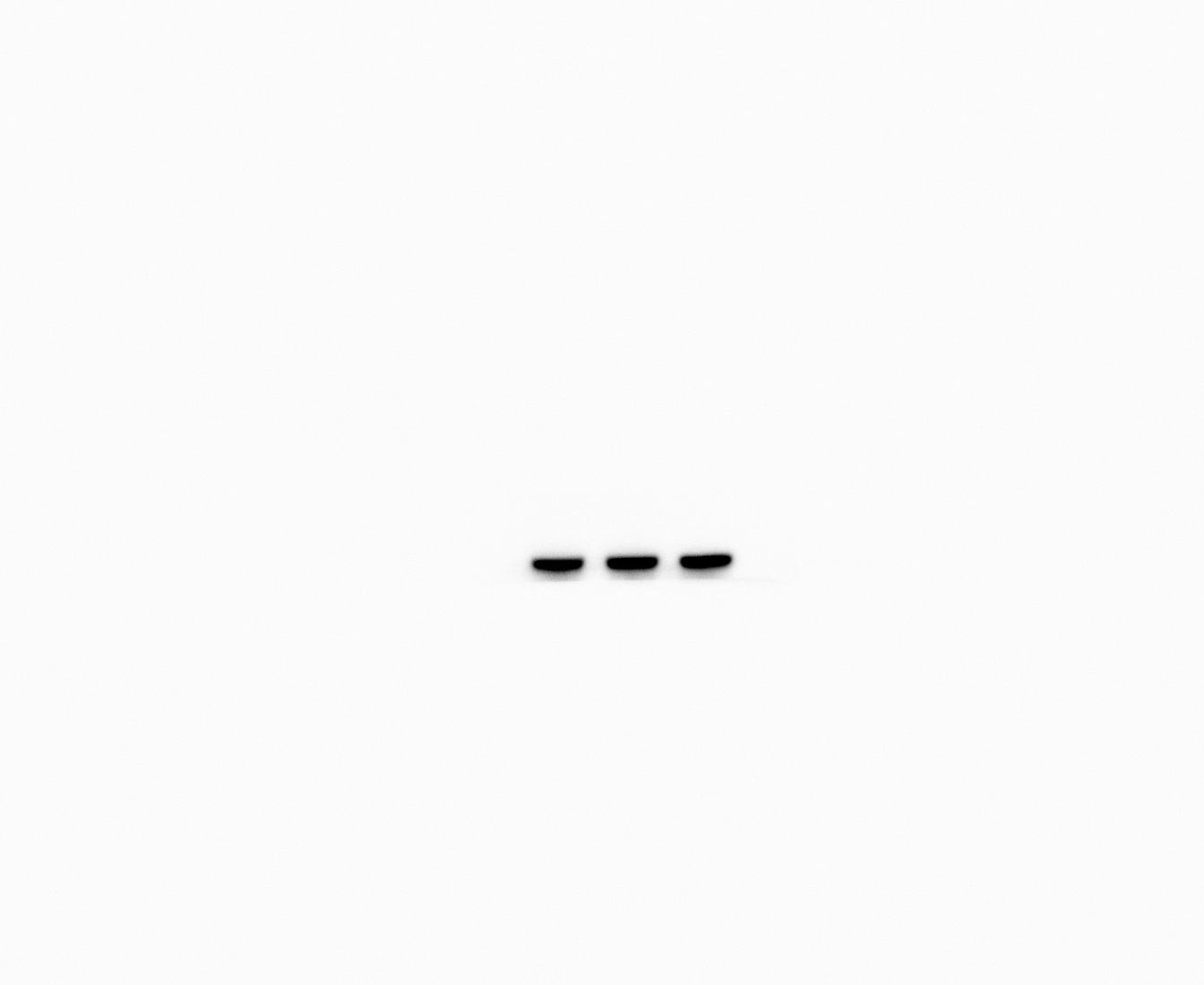

Supplement: Supplementary file 2 — Supplementary Data [file 41420_2026_3089_MOESM2_ESM.zip › Original Data File/Figure8-B Original Data/CAL62-TUB_chemi.tif]

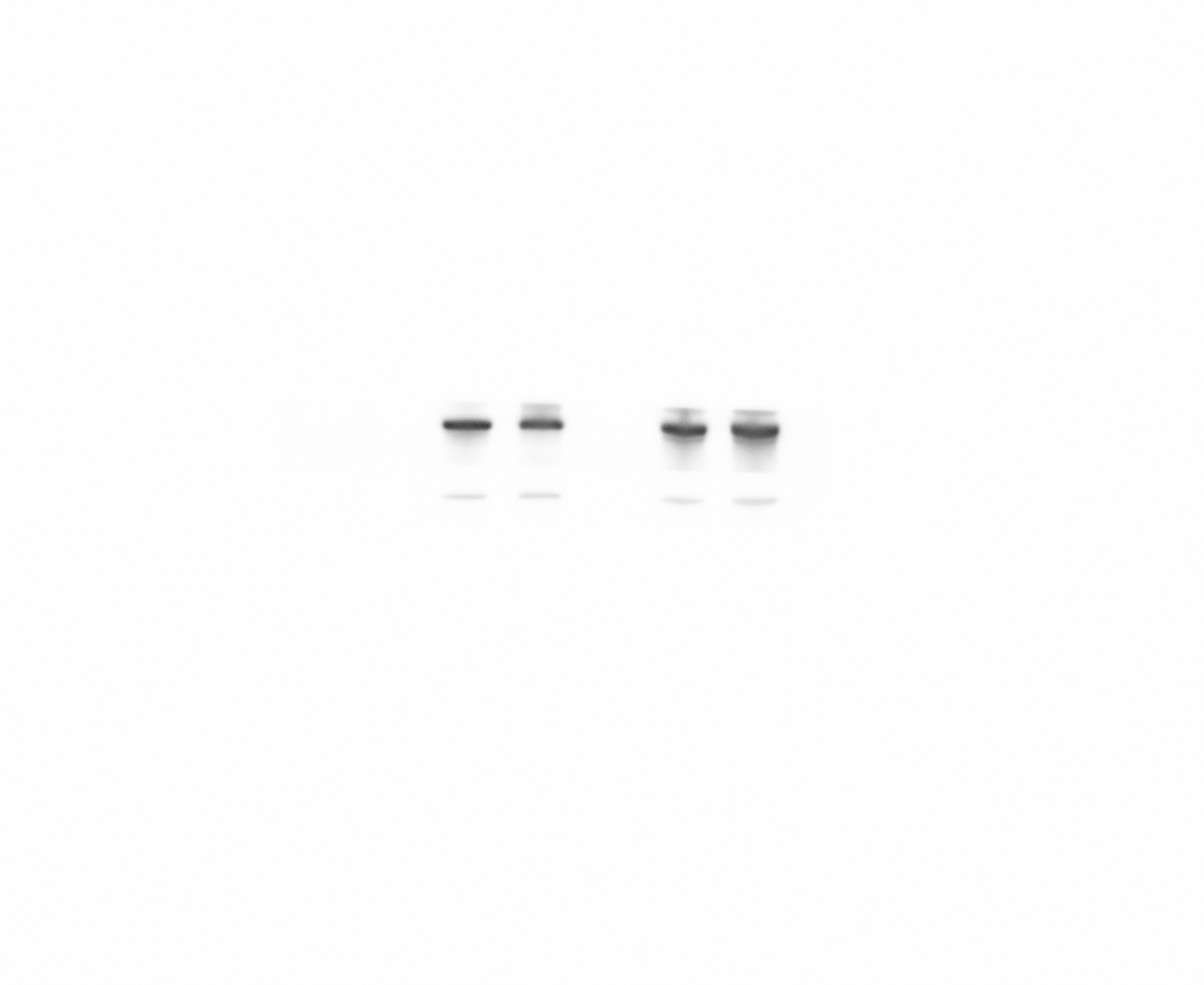

Supplement: Supplementary file 2 — Supplementary Data [file 41420_2026_3089_MOESM2_ESM.zip › Original Data File/Figure7-F Original Data/completeness_chemi.tif]

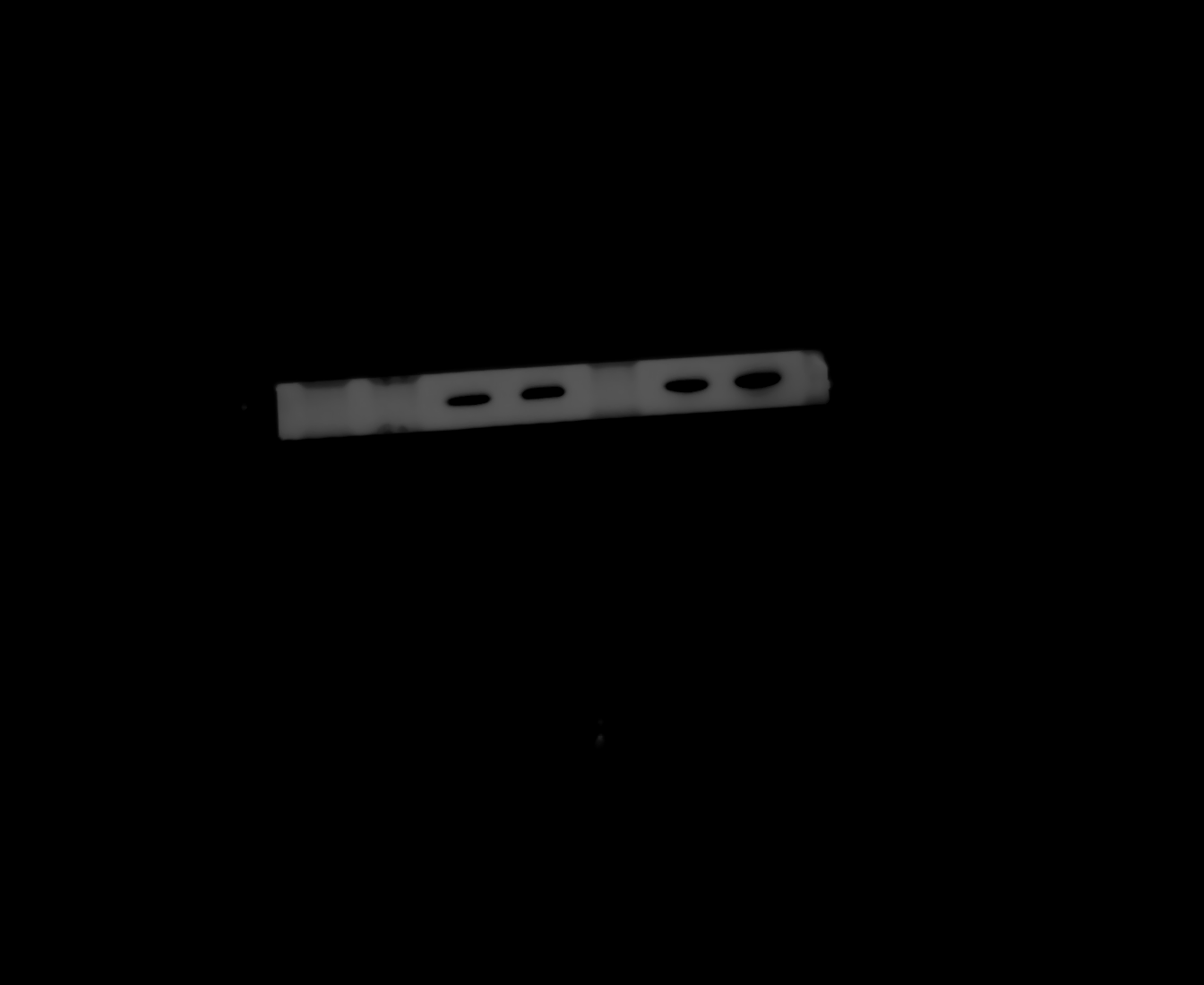

Supplement: Supplementary file 2 — Supplementary Data [file 41420_2026_3089_MOESM2_ESM.zip › Original Data File/Figure7-F Original Data/XCT.tif]

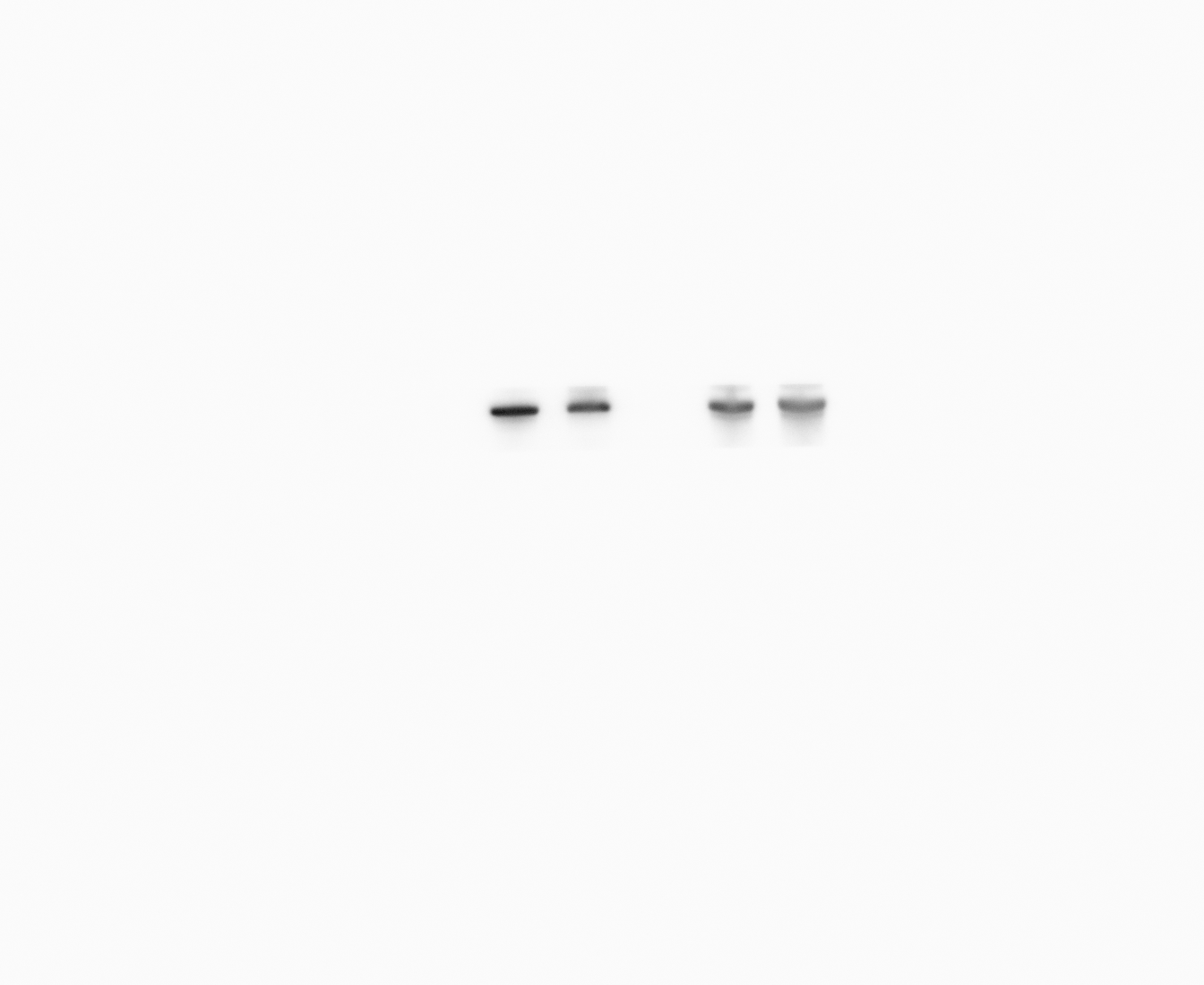

Supplement: Supplementary file 2 — Supplementary Data [file 41420_2026_3089_MOESM2_ESM.zip › Original Data File/Figure7-F Original Data/HSP60_chemi.tif]

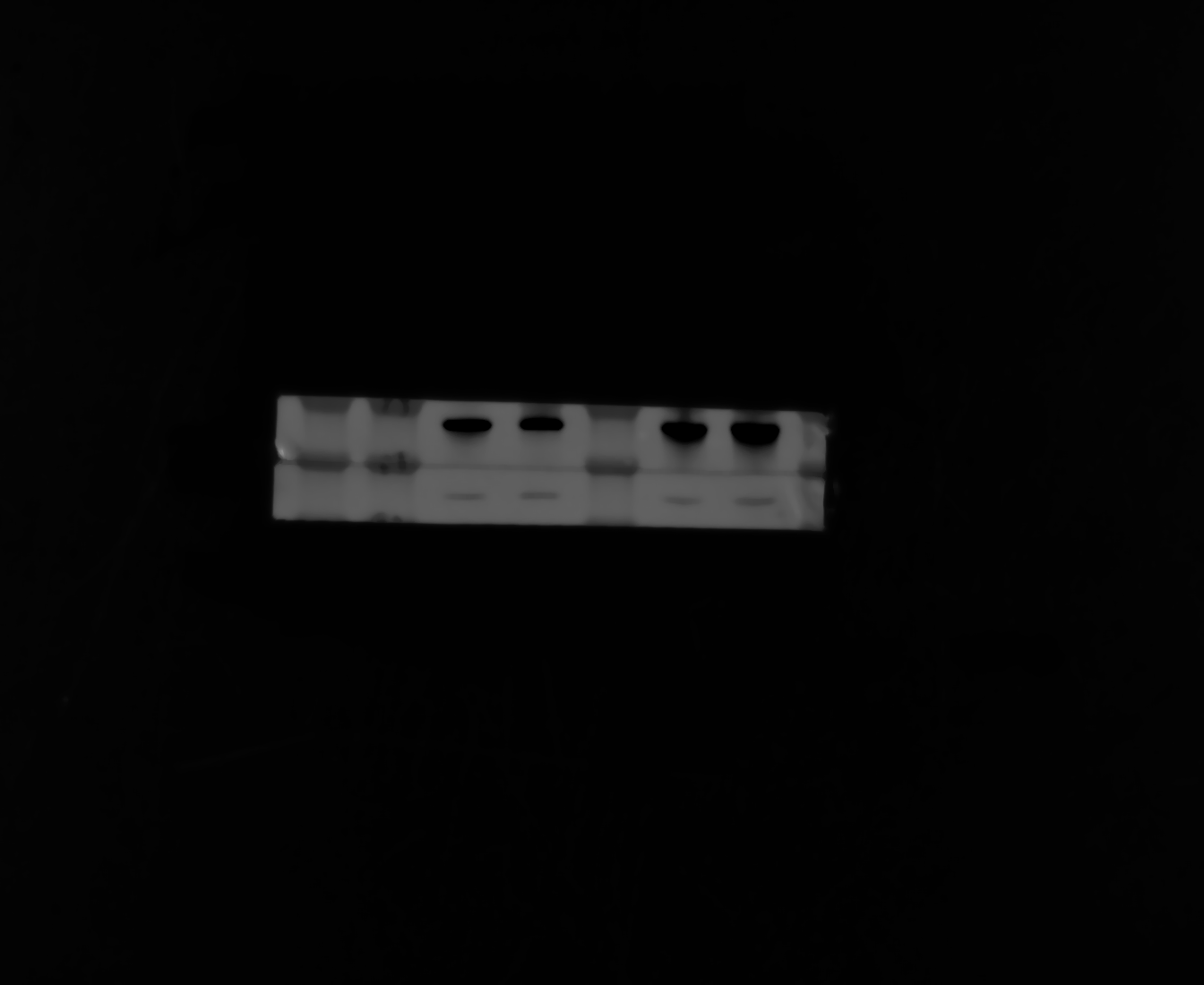

Supplement: Supplementary file 2 — Supplementary Data [file 41420_2026_3089_MOESM2_ESM.zip › Original Data File/Figure7-F Original Data/completeness.tif]

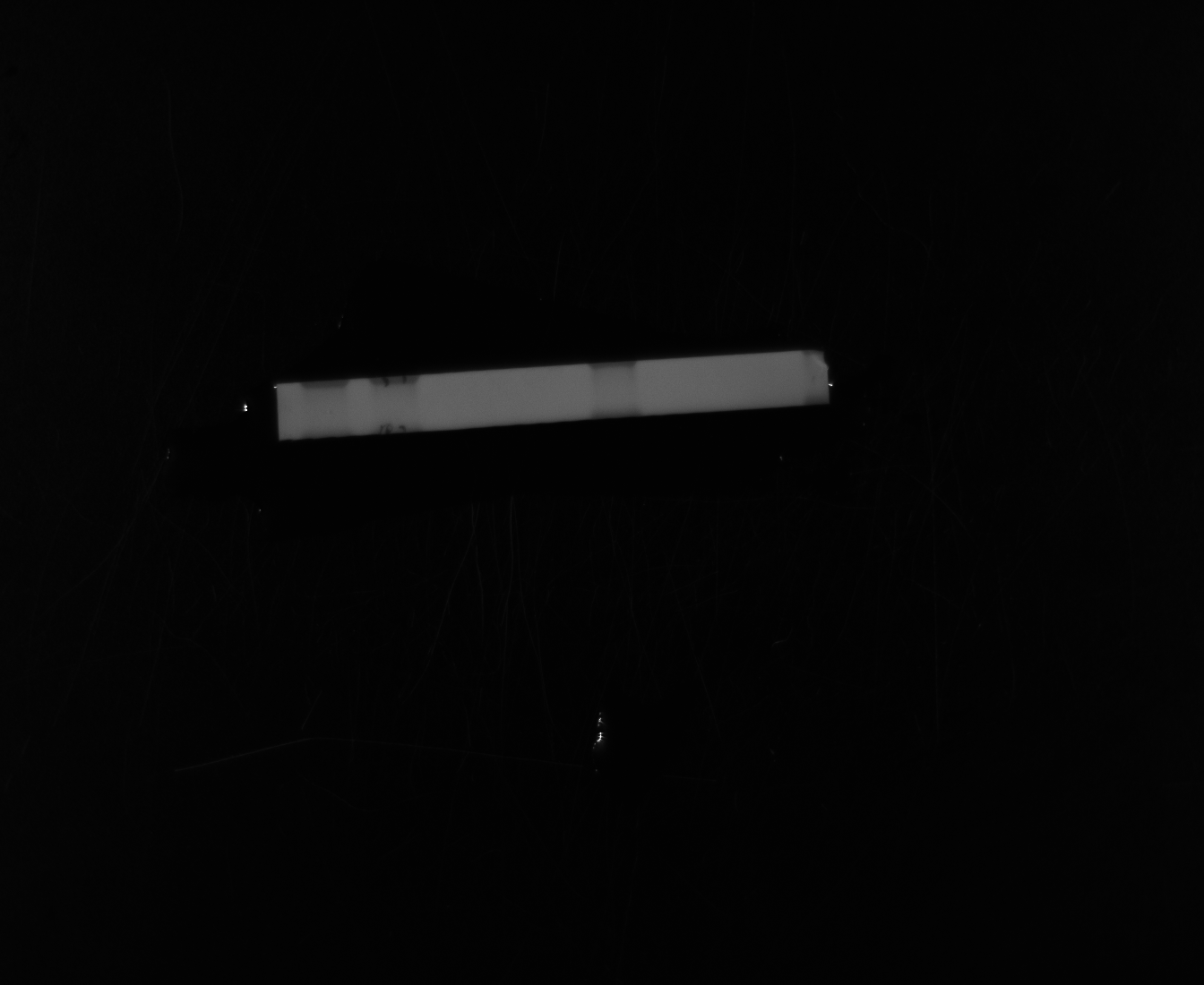

Supplement: Supplementary file 2 — Supplementary Data [file 41420_2026_3089_MOESM2_ESM.zip › Original Data File/Figure7-F Original Data/XCT_marker.tif]

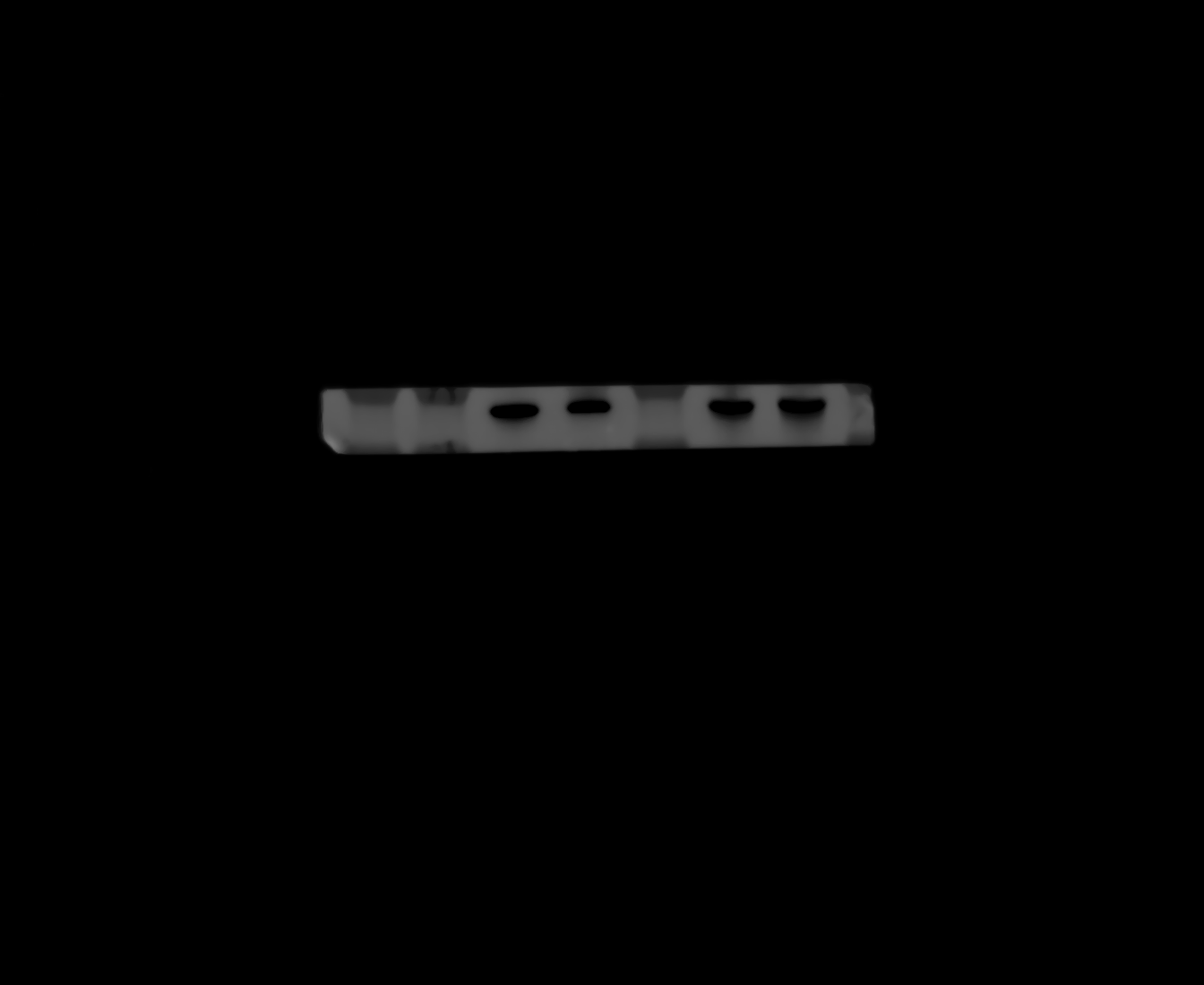

Supplement: Supplementary file 2 — Supplementary Data [file 41420_2026_3089_MOESM2_ESM.zip › Original Data File/Figure7-F Original Data/HSP60.tif]

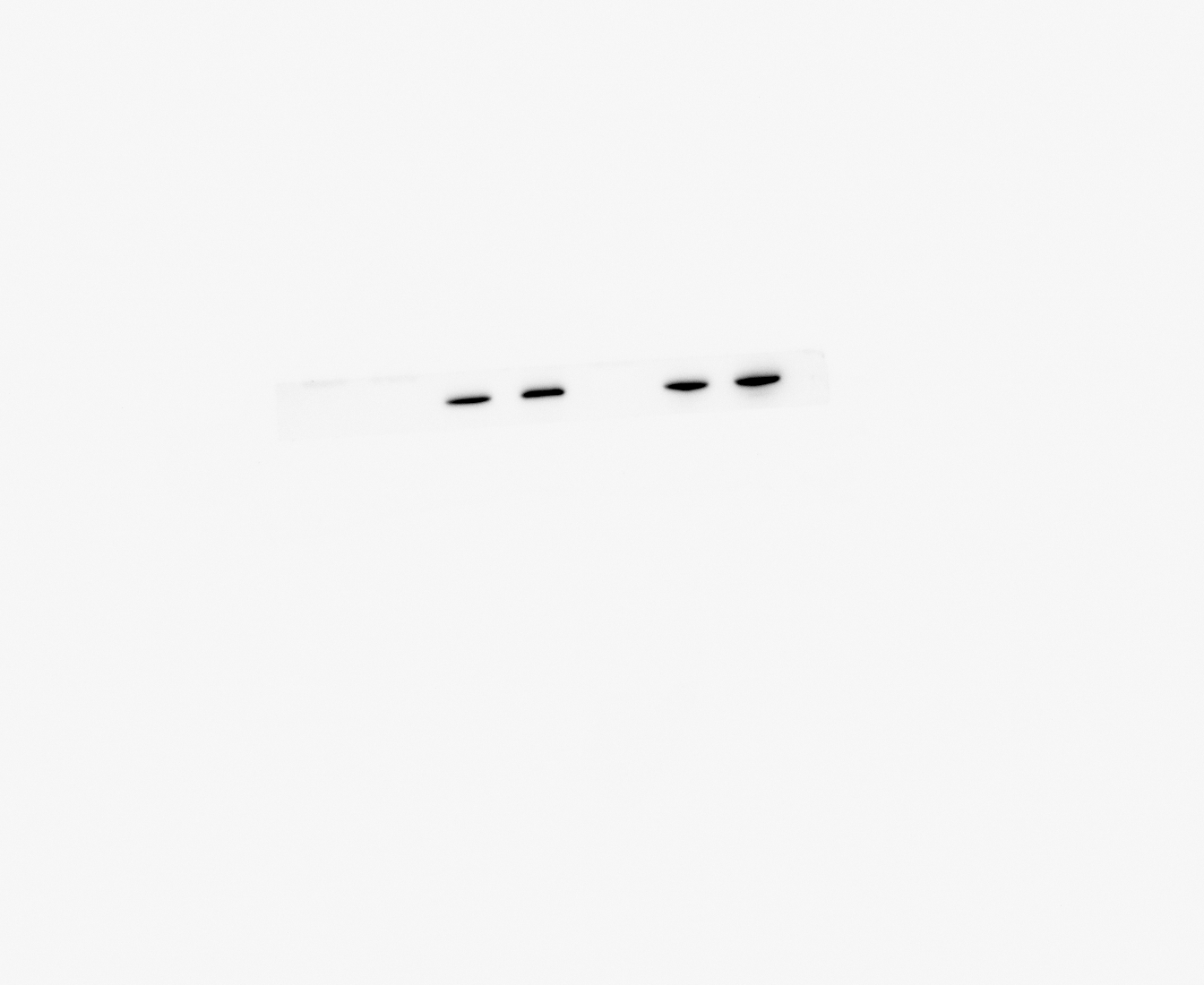

Supplement: Supplementary file 2 — Supplementary Data [file 41420_2026_3089_MOESM2_ESM.zip › Original Data File/Figure7-F Original Data/XCT_chemi.tif]

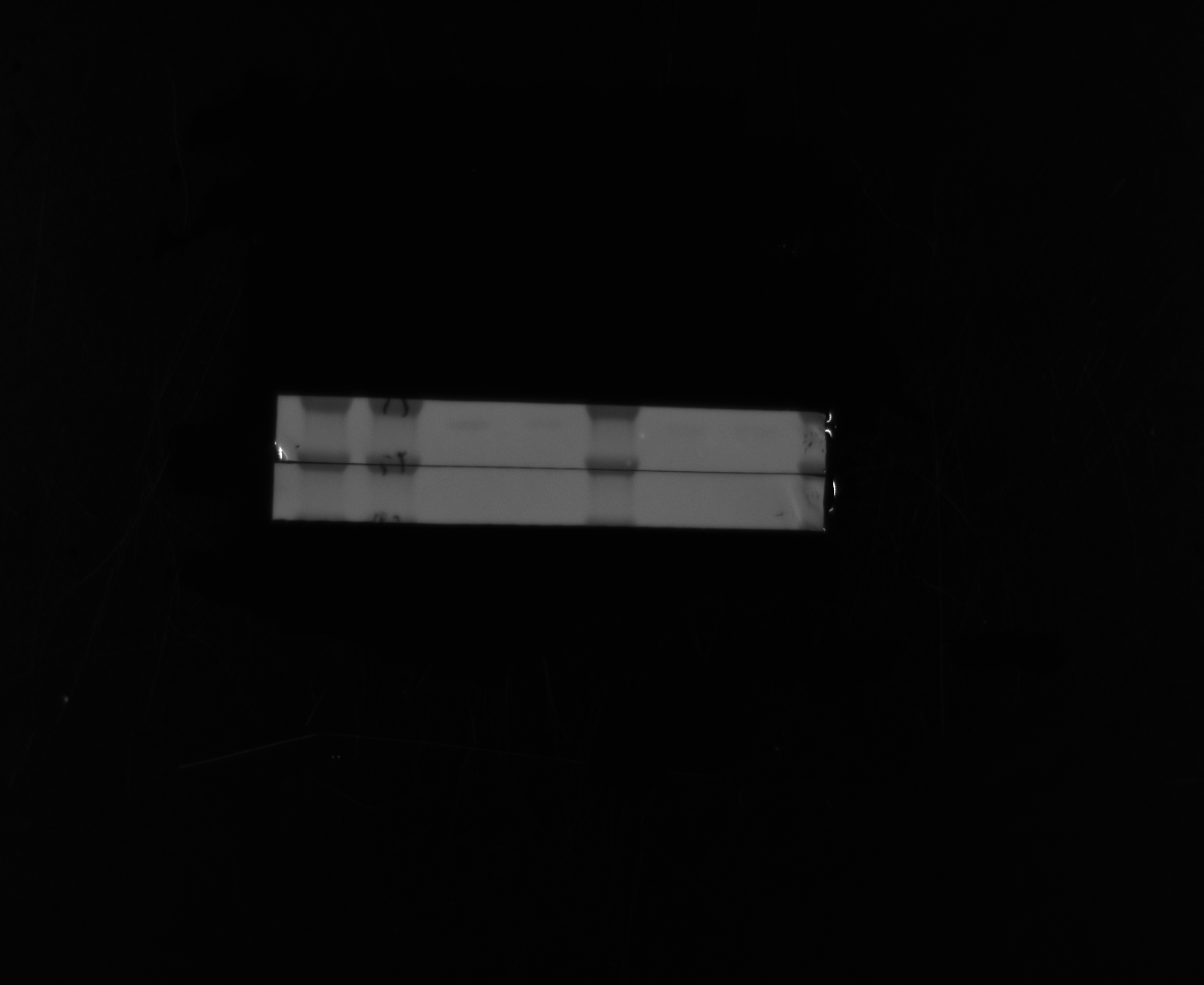

Supplement: Supplementary file 2 — Supplementary Data [file 41420_2026_3089_MOESM2_ESM.zip › Original Data File/Figure7-F Original Data/completeness_marker.tif]

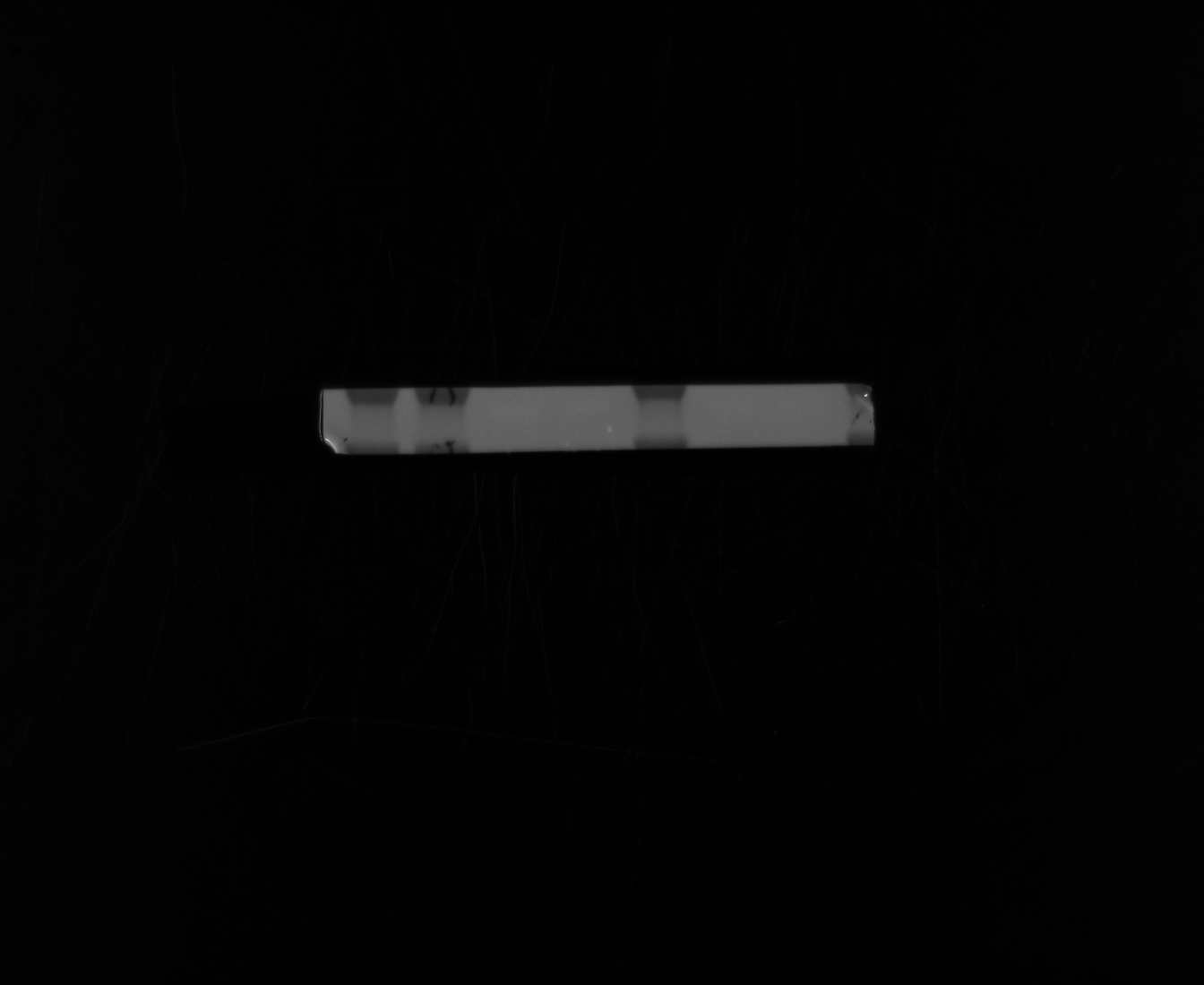

Supplement: Supplementary file 2 — Supplementary Data [file 41420_2026_3089_MOESM2_ESM.zip › Original Data File/Figure7-F Original Data/HSP60_marker.tif]
