# Supplementary material for: Identification and molecular typing of disulfidptosis-related biomarkers in anaplastic thyroid carcinoma
Source: Cell Death Discov. 2026 Apr 22;12:268. doi: 10.1038/s41420-026-03089-9 (PMC13237300; doi:10.1038/s41420-026-03089-9)
Supplement: Supplementary file 1 — Supplementary Date [file 41420_2026_3089_MOESM1_ESM.docx]

***Supplementary Date**

Table S1.16 disulfidptosis-related genes

| gene | geng |
| --- | --- |
| SLC7A11 | IQGAP1 |
| NADPH | FLNA |
| INF2 | FLNB |
| CD2AP | TLN1 |
| PDLIM1 | MYL6 |
| ACTN4 | ACTB |
| MYH9 | DSTN |
| MYH10 | CAPZB |

**Table S1**.16 disulfidptosis-related genes

Table S2.

| TFF3 | UST | PLAUR | YWHAG |
| --- | --- | --- | --- |
| PLEKHH1 | CKB | PBK | PRC1 |
| PCMTD1 | EDN3 | FN1 | TWIST1 |
| CCL28 | ABAT | LGALS1 | MAP4 |
| LRP2 | ANK3 | FAM83D | CLIC4 |
| LRP1B | CITED2 | RND3 | PFDN2 |
| BEX1 | HSD17B6 | CEP55 | KDELR3 |
| TMEM30B | HGD | KIF2C | CD68 |
| SH3BGRL2 | ANGPTL1 | ADAM12 | COL1A2 |
| ESRRG | FGFR2 | CENPE | EIF4EBP1 |
| CLN5 | IFT57 | CENPA | RAI14 |
| PRKCQ | BBS1 | COL5A2 | HPSE |
| BMP7 | DIO1 | SERPINE1 | GMPS |
| MATN2 | AP1M2 | ENO1 | CKAP4 |
| PEBP1 | PREPL | ADM | LDHA |
| GPM6A | MBIP | UCK2 | ATP1B3 |
| MAL2 | SLITRK5 | FRMD6 | TYMS |
| PLCH1 | CROT | CTSA | COL5A1 |
| ELMO1 | RRAGD | P4HA1 | GNAI3 |
| LIPG | TBX3 | ANLN | GGH |
| RYR2 | RNF38 | KIF14 | TPX2 |
| NEBL | PPP1R14C | TMSB10 | FOXM1 |
| FCGBP | PDCD4 | SLC1A5 | CHSY1 |
| PRSS8 | IFT140 | CD86 | RRM2 |
| MYO5C | GPD1L | STEAP1 | ZWINT |
| TXNL1 | SULT1C2 | ARPC2 | SRM |
| PPP1R13B | LMBRD1 | ARL4C | SLC20A1 |
| PCP4 | TMEM158 | NME1 | WDHD1 |
|  |  |  | CCL4 |

**Table S2.**There are 113 common crossover genes in the GSE 65144 and GSE 9115 datasets

**Supplementary Figure Legend**

**Supplementary Figure 1.** Differential expression analysis and WGCNA. **A.** Heat map of differentially expressed genes in the GSE9115 dataset. **B.** Heat map of differentially expressed genes in the GSE65144 dataset. **C.** Dendrogram of discrete clustering of samples. **D.** Graph of module merging. **E.** Dendrogram of gene modules.

**Supplementary Figure 2. The acquisition and functional analysis of candidate genes.**

A. Venn diagram of "common DEGs" and "key module genes". **B.** Chromosomal localization of candidate genes. Each dot in the figure represented one gene, with red indicating up-regulation and black indicating down-regulation. **C.** Protein-protein interaction network of candidate genes. **D.** Results of GO enrichment analysis for candidate genes. **E.** Results of KEGG enrichment analysis for candidate genes.

**Supplementary Figure 3. GSEA (GO and KEGG) enrichment results of biomarkers.**

**A.** GO enrichment of ADAM12. **B.** KEGG enrichment results of ADAM12. **C-D.** ATP1B3 gene. **E-F.** COL1A2 gene. **G-H.** LGALS1 gene. **I-J.** TFF3 gene. The top five line graphs showed the Enrichment Score of gene sets. The ordinate represented the Running Enrichment Score, with a peak in the line graph indicating the Enrichment Score of the gene set. Genes before the peak were core genes within the gene set. The abscissa represented each gene in the gene set, corresponding to the bar-code like vertical lines in the second part, where each line represented a gene in the gene set. The third part showed the distribution of rank values for all genes, with the ordinate being the ranked list metric (i.e., the value of the gene's ranking metric).
